# Lubiprostone improves intestinal permeability in humans, a novel therapy for the leaky gut: A prospective randomized pilot study in healthy volunteers

**DOI:** 10.1371/journal.pone.0175626

**Published:** 2017-04-14

**Authors:** Takayuki Kato, Yasushi Honda, Yusuke Kurita, Akito Iwasaki, Takamitsu Sato, Takaomi Kessoku, Shiori Uchiyama, Yuji Ogawa, Hidenori Ohkubo, Takuma Higurashi, Takeharu Yamanaka, Haruki Usuda, Koichiro Wada, Atsushi Nakajima

**Affiliations:** 1 Department of Gastroenterology and Hepatology, Yokohama City University School of Medicine, Yokohama, Kanagawa, Japan; 2 Department of Biostatistics, Yokohama City University Graduate School of Medicine, Yokoahama, Kanagawa, Japan; 3 Department of Pharmacology, Shimane University School of Medicine, Izumo, Shimane, Japan; University Hospital Llandough, UNITED KINGDOM

## Abstract

**Background and aims:**

The barrier function of the small intestinal mucosa prevents the introduction of undesired pathogens into the body. Breakdown of this barrier function increases intestinal permeability. This has been proposed to induce not only gastrointestinal diseases, including inflammatory bowel disease and irritable bowel syndrome, but also various other diseases, including allergies, diabetes mellitus, liver diseases, and collagen diseases, which are associated with this so called “leaky gut syndrome.” As such, a method to prevent leaky gut syndrome would have substantial clinical value. However, no drugs have been demonstrated to improve disturbed intestinal permeability in humans to date. Therefore, we investigated whether a drug used to treat chronic constipation, lubiprostone, was effective for this purpose.

**Methods:**

Healthy male volunteers were treated with lubiprostone (24 μg/day) for 28 days. Intestinal permeability was evaluated by measuring the lactulose-mannitol ratio (LMR) after administration of diclofenac and compared with an untreated group. The examination was conducted three times in total, i.e., at baseline before diclofenac administration and after 14 and 28 days of lubiprostone treatment. Blood endotoxin activity was also evaluated at the same time points.

**Results:**

The final analysis was conducted on 28 subjects (14 in the lubiprostone group and 14 in the untreated group). The LMR after 28 days of treatment was significantly lower in the lubiprostone group than that in the untreated group (0.017 vs. 0.028, respectively; 95% confidence interval, −0.022–−0.0001; p = 0.049). Blood endotoxin activity exhibited almost no change over time in the lubiprostone and untreated groups and displayed no significant differences at any time point of examination.

**Conclusions:**

This study is the first to report an improvement in leaky gut using an available drug in humans. The result suggests that lubiprostone may prevent and ameliorate “leaky gut syndrome”. However, a pivotal trial is needed to confirm our finding.

## Introduction

Epithelial cells of the intestinal tract are involved in nutrient absorption, a function essential for life. However, they also play a central role in barrier function by preventing pathogen intrusion into the body. These cells form not only tight junctions (TJs), which serve as a physical barrier preventing intrusion of foreign matter from the paracellular space, but also thick mucus layers, thereby playing an active role in preventing pathogen entry into the host. TJs selectively enable permeation of intestinal contents, while permeability may be physiologically increased in response to nutrients in the intestinal tract. Moreover, barrier function may be injured and destroyed by immune cells and cytokines in the mucosa, or even by pathogens, which results in “leaky gut.” Abnormal barrier function has been shown to be involved in not only intestinal diseases, including inflammatory bowel disease and irritable bowel syndrome [1–3], but also various systemic diseases, including food allergies [4], diabetes mellitus [5], metabolic diseases, such as nonalcoholic fatty liver/nonalcoholic steatohepatitis [6], and collagen diseases [7]. These disorders are therefore considered to represent “leaky gut syndromes.” Thus, the pathology of leaky gut has attracted significant attention among the general public. Leaky gut is expected to induce conditions in which harmful substances in the intestinal tract, such as microorganisms containing endotoxins, enter the body and are systemically circulated. Even if the amount of such substances is small, thrombotic events, such as myocardial infarction and cerebral infarction, may be induced if such a condition persists chronically [8].

As described above, leaky gut may be involved in the pathogenesis of not only classical gastrointestinal diseases but also diseases affecting various other organs, and a measure to improve this situation is urgently needed. Nonsteroidal anti-inflammatory drugs (NSAIDs), which are commonly used in daily practice, have been demonstrated to increase intestinal permeability [9] in both humans and animal models [10–12]. These results indicate that there may be more patients with underlying leaky gut than expected. While these patients may not suffer from clinical symptoms, intestinal permeability control would nevertheless be of clinical importance to prevent them from developing severe complications. Therefore, the discovery of a drug that prevents leaky gut may become key to the prevention of various diseases and complications and would provide a great clinical impact. However, there are no available medications that are effective at treating leaky gut to date.

Lubiprostone (brand name: Amitiza^®^ Capsule 24 μg, marketed in Japan by Mylan EPD) is a ClC-2 chloride channel activator approved in November 2012 in Japan for the treatment of chronic constipation. The potential of the drug to prevent small intestinal injury related to NSAIDs has been reported in animal experiments [12]. Thus, we evaluated lubiprostone as a candidate for the first drug ever to improve the state of leaky gut.

Accordingly, we first treated healthy volunteers with NSAIDs and then lubiprostone, with the aim of evaluating whether the drug was effective in preventing increased intestinal permeability. Additionally, we assessed the extent to which the drug affected endotoxin concentrations in the blood.

## Materials and methods

This study was approved by the Medical Research Ethics Committee of Yokohama City University, and informed consent was obtained from all subjects in writing. This study has been registered in UMIN-CTR (Registration No. UMIN000017342).

### Subjects

**Fig 1:**
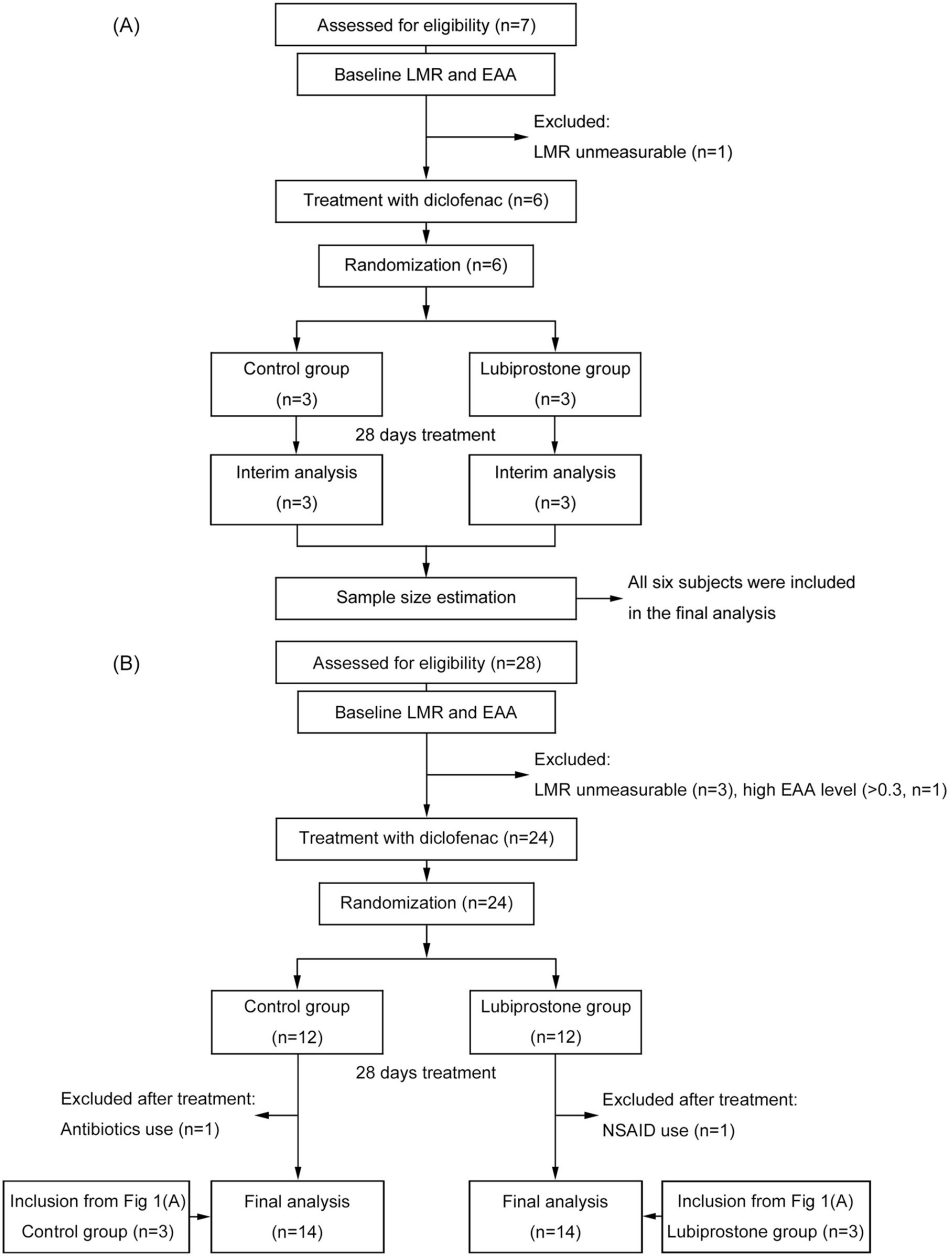
Flow diagram for entry of subjects and implementation of the study. (A) Study protocol for the first six subjects, (B) Study protocol for the remaining 28 subjects.

The inclusion criteria of the subjects in this study were as follows: (1) male; (2) age 20 to 60 years; (3) good health condition; and (4) informed consent provided in writing and ability to comply with the study protocol. The exclusion criteria were as follows: (1) administration of NSAIDs within 3 months before informed consent (except for topical application of external preparations); (2) a history of suspected drug allergy; (3) current or previous serious cardiovascular, respiratory, or liver/kidney/gastrointestinal system disease or a neuropsychiatric disorder; (4) proton pump inhibitor, antibiotic (except for topical application), and/or probiotic use; (5) participation in another clinical study within 1 month before the start of this study and receipt of a study drug; and (6) ineligibility to be included as subjects of this study, as deemed by the principal investigator. The target number of subjects to be recruited was 35 in total. Volunteer enrollment began on Aug. 1, 2015, and ended when the target sample size was reached on Feb. 16, 2016. Follow-up of the subjects ended on Mar. 15, 2016. The flow diagram of the progress of participants through the trial is displayed in Fig 1.

### Study design

**Fig 2:**
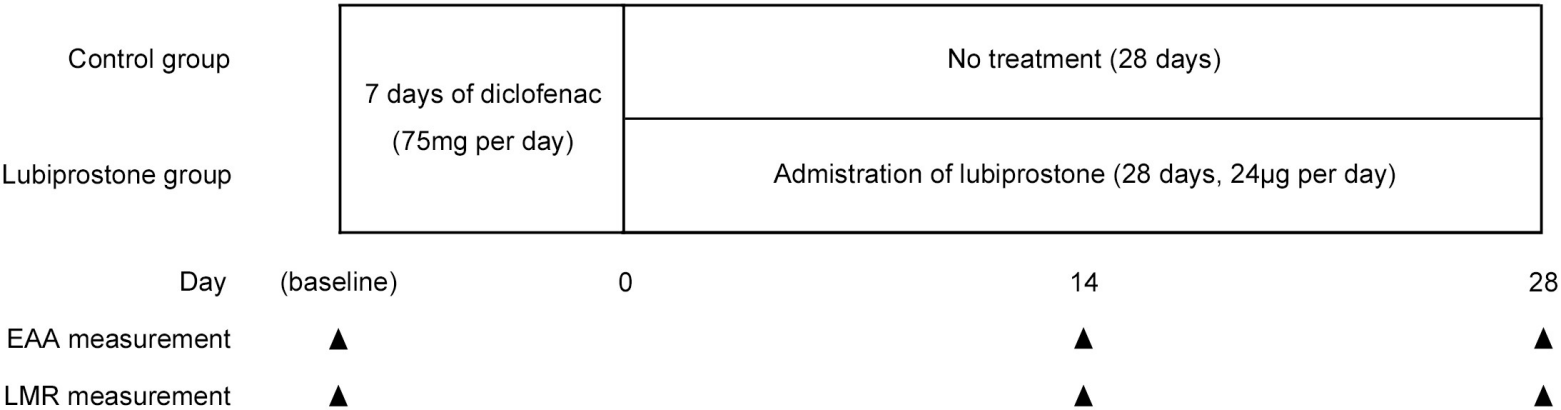
Study protocol.

The study was designed as an open-label, randomized, parallel-group comparison study. The subjects in the study drug group (lubiprostone group) were treated with 75 mg/day diclofenac, which is the standard therapeutic dose in Japan, for the first 7 days, followed by 24 μg/day lubiprostone for 28 days. The subjects in the untreated group (control group) were treated with 75 mg/day diclofenac for the first 7 days only. The dose of lubiprostone chosen is the lowest administration dose possible in Japan (where only 24 μg capsules are approved). This dose was selected, because adverse events associated with lubiprostone are reported to occur dose-dependently, while the clinical efficacy of lubiprostone in irritable bowel syndrome did not differ substantially between different lubiprostone doses above 16 μg/day [13]. The 14- and 28-day treatment assessment periods were selected based on the treatment periods assessed in previous studies using lubiprostone for chronic idiopathic constipation treatment in Japan [14, 15]. The study protocol is summarized in Fig 2.

### Randomization

Registration, randomized allocation, and data collection were performed at Yokohama City University School of Medicine. Block randomization with a block size of four (size of six for the first six subjects to determine the study period) based on a random number list provided by a computer program was used for sorting.

### Endpoints

The primary endpoint was the evaluation of intestinal permeability using the lactulose-mannitol ratio (LMR) in urine. The secondary endpoint was the evaluation of blood endotoxin levels using the blood endotoxin activity assay (EAA).

### Evaluation methods

The tests were conducted three times in total: before diclofenac administration (baseline); following 7 days of diclofenac administration and 14 days of treatment with or without lubiprostone administration (day 14); and after 28 days of lubiprostone treatment (day 28). Based on a prior report [16], LMR was determined using 4-h pooled urine collected from subjects who had fasted for at least 12 h, emptied their bladder, and had orally consumed a sugar solution prepared by dissolving 10 g of lactulose and 5 g of mannitol in 400 mL of water. During the fasting period, subjects were allowed to drink sugar-free water. They drank 200 mL of additional water 2 h after oral intake of the sugar solution. Collected urine was preserved in a freezer for liquid chromatography-mass spectrometry (LC/MS) analysis. For the EAA, blood specimens were collected after oral intake of the above sugar solution and were incubated at room temperature for 1 h. Endotoxin levels were then determined with a measuring device (Toray, Japan). The EAA quantifies endotoxin levels via a relative scale from 0 to 1.0 based on an internal endotoxin standard that is added to each sample. Endotoxin activity is measured by stimulation of the neutrophil respiratory burst with zymosan and emission of light via reaction of oxidants (primarily HOCl) with luminol. Activity is then calculated by an algorithm based on light emission measured in a luminometer sensitive to light in the 450-nm range (Patient sample with antibody − Patient sample without antibody)/(Patient sample with maximal endotoxin − Patient sample without antibody) [17, 18]. This method is mainly used in the critical care field, and EAA levels are reported to increase in patients in the intensive care unit and decrease after treatment [19, 20].

### Urine sample preparation for LC/MS analysis

Urine samples were diluted 1:10 with 80% acetonitrile aqueous solution (dilution solution) and centrifuged at 4000×*g* for 5 min. The supernatant was then diluted 1:5, and the centrifugation step was repeated. Obtained samples were additionally diluted 1:100 (for lactulose quantitation) and 1:500 (for mannitol quantitation). From these samples, 120 μL were transferred to 96-well plates, and the plates were transferred to the autosampler (Model: SIL-HTc, Shimadzu Co., Tokyo, Japan), which was connected to a high-performance liquid chromatography pump (Model: LC-10AD VP Shimadzu Co., Tokyo, Japan).

### Quantitation of lactulose and mannitol by LC/MS/MS analysis

Samples were separated by high-performance liquid chromatography using a 150×2 mm, Luna 5 μm NH2 100 Å column (Phenomenex, CA, USA) at 50°C. Lactulose and mannitol were eluted with 80% acetonitrile in water for the first 0.5 min. The acetonitrile concentration was then changed to a linear gradient from 80% to 50% for 1 min, 50% to 80% for another 1 min, and the 80% elution was then maintained for 4.5 min. The flow rate was 350 μL per min for all separation steps. Lactulose and mannitol were quantified using a triple quadrupole/ion trap mass spectrometer (Model: LCMS-8030, Shimadzu Co., Tokyo, Japan) with negative-ion mode. The Q1 and Q3 quadrupoles were tuned for a unit mass resolution instrument. Data were acquired and processed with LabSolutions software (Shimadzu Co., Tokyo, Japan).

### Statistical analysis and sample size determination

EZR software on R commander for biostatistics was used for statistical analysis [21]. The Wilcoxon signed-rank test was used for comparison of time-course changes within the same group, and analysis of covariance was used to compare the two groups. Welch test was used for baseline comparison of the two groups, and Fisher’s exact test was used for the safety comparison.

Since there were no similar previous reports for sample size estimation, we estimated the sample size to be approximately 35, including dropouts, based on a study reporting that a novel compound had affected LMRs in healthy volunteers [22] as a reference. We also performed a sample size estimation and determined the study period after analysis of the first six subjects (three subjects in each group). Thus, we first performed the block randomization with a block size of six. From the results of the first six subjects, the mean LMR difference on day 28 between the two groups was 0.012. The LMR standard deviation on day 28 was 0.0032 in the lubiprostone group and 0.011 in the control group. From these results, the sample sizes were calculated to range from 2 to 17 per group. We estimated that with this sample size, the study would have 90% power to detect a statistical difference in the LMR on day 28 between the two groups, with a two-sided type I error of 0.05. We calculated the sample size to match the same conditions for LMR on day 14. However, the estimated sample sizes ranged from 272 to 646 per group, which were too large for a pilot study. Based on these results, we decided to use a 28-day treatment period and a sample size of 28 additional subjects to match the largest estimated sample size and account for some dropouts.

## Results

Among the 35 subjects who fulfilled the inclusion criteria, baseline LMRs could not be determined in four subjects, and the EAA value was higher than the assay cutoff (>0.3) in one subject. Therefore, 30 subjects were enrolled and randomly assigned to groups of 15 subjects each. One subject in the lubiprostone group was excluded from the final analysis because of oral intake of an NSAID during the lubiprostone treatment period. Additionally, one subject in the control group was excluded because of oral intake of antibiotics during the study period. Both subjects were identified during the medical interview performed on day 28. There were no dropouts because of adverse drug reactions to the investigational product (Fig 1).

### Baseline characteristics

There were no significant differences between the two groups in age, BMI, or smoking habits (Table 1).

**Table 1:** Baseline characteristics of the two groups.

	Control group (n = 14)	Lubiprostone group (n = 14)	95% CI differences between the two groups	p value
Age (years old)	23.7±2.29	22.9±2.95	-2.9–1.2	0.40
BMI (kg/m^2^)	22.6±2.73	22.7±1.73	-1.7–1.9	0.94
Smoker	1/14 (7%)	1/14 (7%)	N/A	N/A

Data are expressed as mean ± standard deviation. Welch test was used for statistical analysis.

Abbreviations: BMI, body mass index; 95% CI, 95% confidence interval

### Urinary LMR

**Fig 3:**
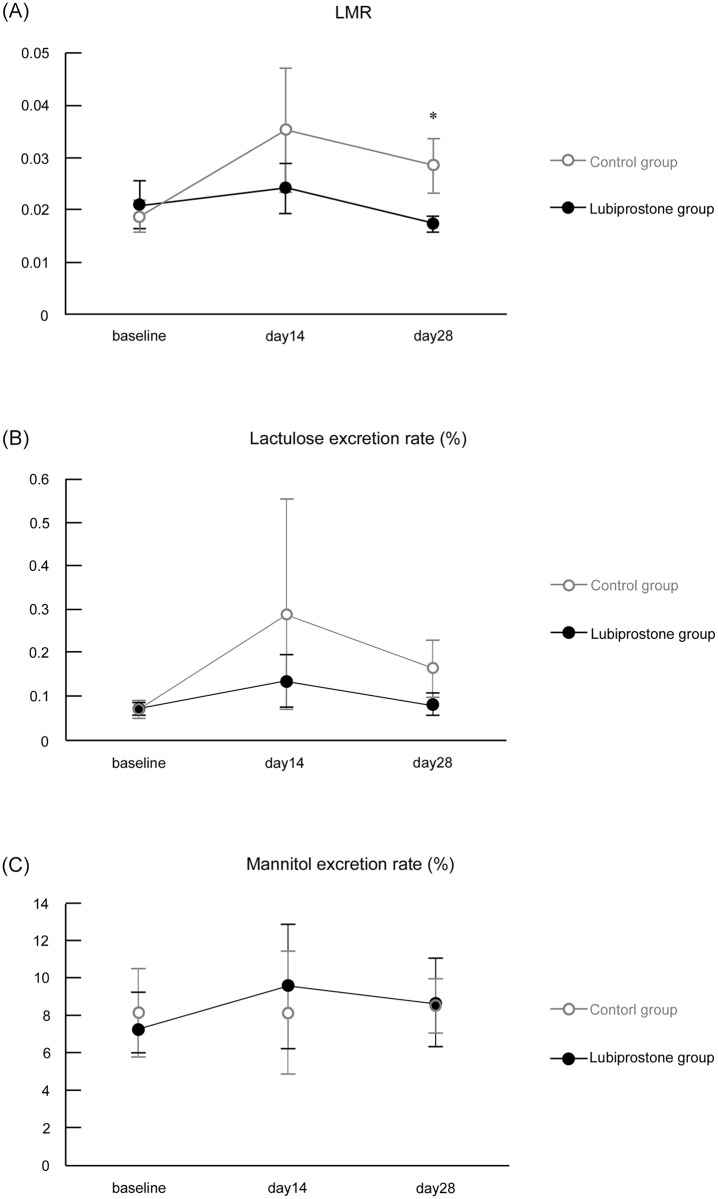
Urinary LMRs and lactulose and mannitol excretion rates. (A) LMRs, (B) lactulose excretion rate, and (C) mannitol excretion rate. Data are expressed as mean ± standard error. Analysis of covariance (*p<0.05) or Welch test (baseline, or due to significant interaction between grouping variable and covariate; day 14 and day 28 of lactulose excretion rate and day 28 of mannitol excretion rate) were used for statistical analysis. Abbreviations: LMR, lactulose-mannitol ratio.

No significant differences were observed in the mean LMRs at baseline between the two groups. The ratio on day 14 was lower in the lubiprostone group than that in the untreated group, but this difference was not significant. On day 28, the LMR in the lubiprostone group was significantly lower than that in the untreated group (95% confidence interval, −0.022 –−0.0001; p = 0.049). There were no significant differences between baseline, day 14, and day 28 within each treatment arm. Lactulose and mannitol excretion rates were also examined, but no significant differences were observed at the time points analyzed in the two groups. (Table 2, Fig 3)

**Table 2:** Urinary LMRs and lactulose and mannitol excretion rates.

	Control group (n = 14)	Lubiprostone group (n = 14)	95% CI differences between the two groups	p value
LMR				
baseline	0.019±0.003	0.021±0.005	-0.009–0.014	0.70
day 14	0.035±0.012	0.024±0.005	-0.035–0.024	0.40
day 28	0.028±0.005	0.017±0.002	-0.022 –-0.0001	0.049*
Lactulose excretion rate (%)				
baseline	0.069±0.020	0.070±0.015	-0.070–0.069	0.95
day 14	0.288±0.219	0.134±0.060	-0.639–0.331	0.51†
day 28	0.164±0.048	0.082±0.026	-0.231–0.068	0.27†
Mannitol excretion rate (%)				
baseline	8.162±2.339	7.634±1.626	-6.418–5.362	0.86
day 14	8.172±3.274	9.591±3.298	-8.133–10.972	0.52
day 28	8.531±1.434	8.726±2.369	-5.559–5.947	0.95†

Data are expressed as mean ± standard error. Analysis of covariance (*p<0.05) or Welch test (baseline, or †due to significant interaction between grouping variable and covariate) were used for statistical analysis.

Abbreviations: LMR, lactulose-mannitol ratio; 95% CI, 95% confidence interval

### Blood EAA

**Fig 4:**
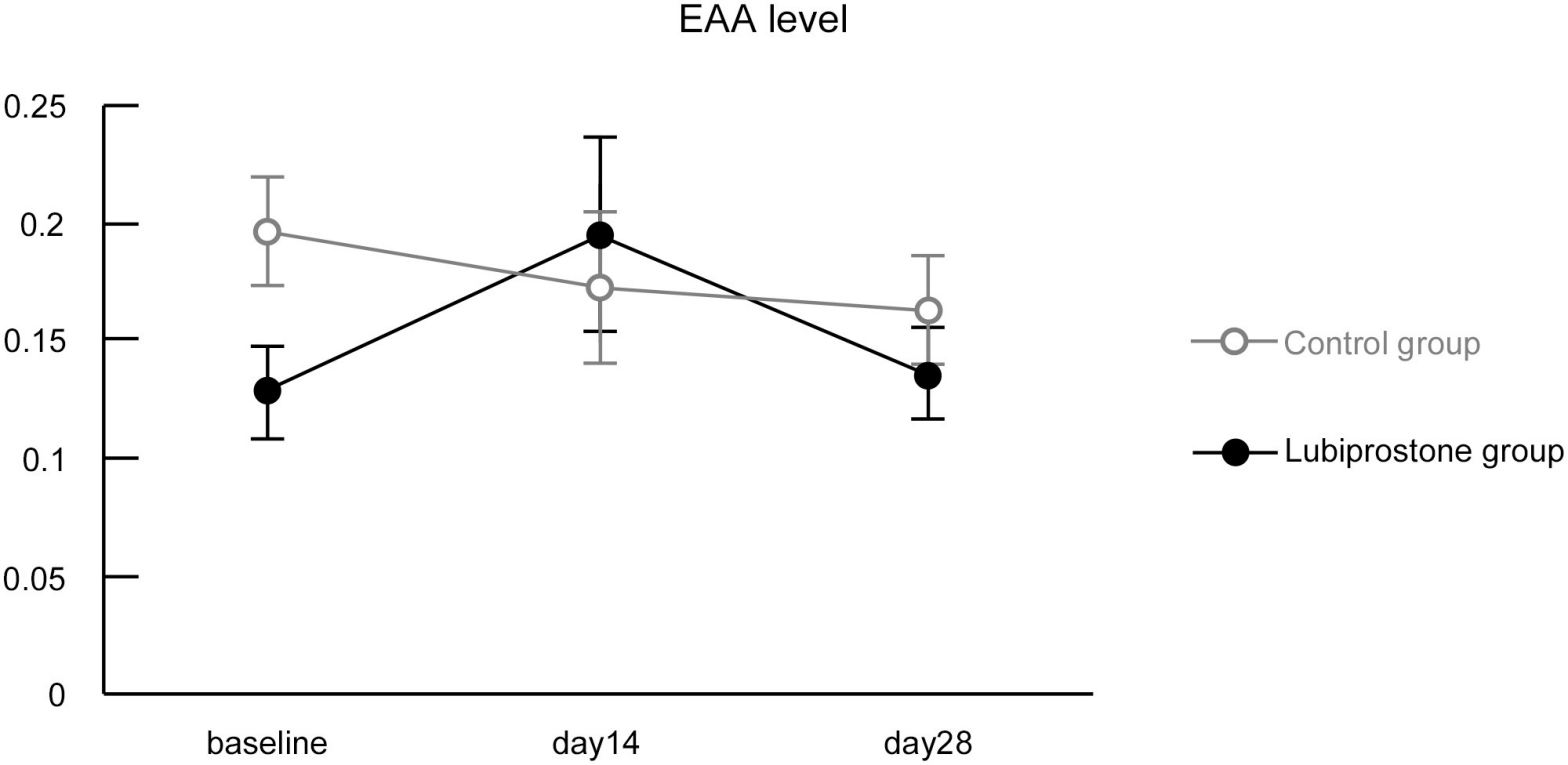
Blood EAA levels. Data are expressed as mean ± standard error. Analysis of covariance or Welch test (baseline) were used for statistical analysis. Abbreviations: EAA, endotoxin activity assay.

Changes in blood EAA levels over time were very small in the lubiprostone and untreated groups, and no significant differences were observed at the time points examined (Table 3, Fig 4).

**Table 3:** Blood EAA levels.

EAA levels	Control group (n = 14)	Lubiprostone group (n = 14)	95% CI differences between the two groups	p value
Baseline	0.20±0.03	0.13±0.02	-0.15–0.02	0.13
day 14	0.17±0.03	0.21±0.05	-0.07–0.16	0.091
day 28	0.16±0.03	0.14±0.02	-0.11–0.06	0.75

Data are expressed as mean ± standard error. Analysis of covariance or Welch test (baseline) were used for statistical analysis.

Abbreviations: EAA, endotoxin activity assay; 95% CI, 95% confidence interval

### Drug safety

No adverse events were observed in either group during the diclofenac treatment period. During the experimental treatment period, no adverse events were observed in the untreated group, but adverse events were reported in 26.7% (4/15, p = 0.10) of subjects in the lubiprostone group. The intensity of these adverse events was mild in all four subjects. Two had diarrhea (13.3%), one had nausea (6.7%), and one had a brief sense of dyspnea (6.7%).

## Discussion

### Evaluation of intestinal permeability

Urinary LMR analysis revealed that 28 days of lubiprostone treatment inhibited intestinal permeability acceleration. The LMR was compared, because the mannitol excretion rate is proportional to the small intestinal mucosal area, in which individual differences in normal absorption are observed (for example, an abnormally low value is observed in celiac disease). In contrast, determination of the excretion rate of lactulose, which has a molecular weight that is not usually absorbed in the small intestine, reveals the extent of damage to the normal mucosa with absorption of mannitol [23]. Therefore, the absence of significant differences in lactulose and mannitol excretion rates between the groups suggests that the state of the small intestinal mucosa at that specific time and the speed of passage of sugar water were different. Our results revealed that lubiprostone inhibited the LMR increase caused by NSAIDs. This indicates that lubiprostone may be able to treat at least mild leaky gut conditions, a finding that has never before been described for any other medication readily available in a clinical study.

### Permeability and endotoxin levels

We initially anticipated that blood endotoxin concentrations would increase in association with accelerated intestinal permeability. However, no significant differences were observed in blood EAA values between the groups, and no correlation was observed between the endotoxin concentration and intestinal permeability acceleration in this study. Although the method of the endotoxin measurement and the drug used (aspirin) were different, there was a previous report comparing LMRs and levels of lipopolysaccharide (LPS), an endotoxin, with a similar result [24]. A possible explanation is that the molecular weight of lactulose is 342 Da, while that of LPS is approximately 10,000 Da, which is a large difference. Therefore, it is likely that even if NSAID treatment accelerated permeability to the extent that substances with a molecular weight similar to that of lactulose leak out of the extraintestinal space, the permeability damage following NSAID treatment for 1 week may not be sufficient for LPS to leak. Another possible reason is that since endotoxin levels were determined in peripheral blood, this was following a first pass through the liver. It would be extremely difficult to collect samples of peripheral blood prior to this first pass, and as such, we did not perform this in this study. Establishment of a permeability test using substances with larger molecular weights, or examination of the correlation between LMRs and EAA values in subjects with decreased liver function is necessary to verify the above possible causes.

### Novelty of the study

In previous studies, treatments for increased intestinal permeability have included a novel complex [22], probiotics *in vitro* [25], or treatments of pre-existing disorders [26, 27]. Additionally, one study used a supplementation [28], but intestinal permeability in this report was evaluated by an indirect parameter using stool concentrations of a TJ component. Our study is the first to report efficacy of an available drug against intestinal permeability acceleration, evaluated by LMRs, which are the gold standard for testing intestinal permeability in humans.

### Limitations

Since this study was conducted in healthy volunteers treated with an NSAID (diclofenac), lubiprostone efficacy against intestinal permeability in patients with other disorders remains unknown, particularly regarding intestinal permeability acceleration caused by mechanisms other than cyclooxygenase inhibition. It has been previously reported that lubiprostone may protect the mucosal barrier by increasing prostaglandin E2 via an E-type Prostanoid (EP)-dependent mechanism [12, 29], but this may only be relevant for leaky gut related to cyclooxygenase inhibition. However, lubiprostone has been reported to activate the EP1 and EP4 receptors [29], while a previous study reported that an EP2 receptor agonist inhibited diclofenac-induced cytokine stimulation in nasal polyp cells [30]. In addition, it was reported in a basic study that the chloride channel ClC-2 modulates the mucosal barrier by trafficking occludin, a tight junction protein [31]. This is likely the primary mechanism of lubiprostone, as the compound is a specific and selective ClC-2 activator. Thus, lubiprostone may be effective in other clinical situations in addition to NSAID-induced enteropathy. Further investigation in such patients is required for verification.

The difference in the primary endpoint (LMR) between the lubiprostone and control groups was only marginally statistically significant (p value = 0.049), and we did not apply any multiple comparison methods. Since this was a pilot study, the reproducibility of this result remains unconfirmed. Additionally, the clinical significance requires further analysis, because there have been no comparisons between LMR changes and clinical outcomes reported to date.

## Conclusions

The present study was the first to report the inhibition of intestinal permeability acceleration using an available drug in humans. Healthy volunteers were treated with lubiprostone for 28 days, and significant inhibition of acceleration was observed following NSAID administration. This result suggests that lubiprostone could become a drug that prevents and improves various diseases related to intestinal permeability. Our pilot study was focused on generating a new hypothesis, and a pivotal trial is needed to confirm our finding.

## Supporting information

S1 Protocol Study protocol written in English. (DOCX)

S2 Protocol Study protocol written in Japanese (original language). (DOCX)

S1 File Log file of the sample estimation analysis. (PDF)

S2 File Log file of the statistical analysis. (PDF)

S3 File Log file of the 95% CI calculation. (PDF)

S1 Dataset Primary data of the study. (CSV)

S1 Checklist CONSERT 2010 checklist for this manuscript. (DOC)
