# Supplementary material for: Lubiprostone improves intestinal permeability in humans, a novel therapy for the leaky gut: A prospective randomized pilot study in healthy volunteers
Source: PLoS One. 2017 Apr 14;12(4):e0175626. doi: 10.1371/journal.pone.0175626 (PMC5391961; doi:10.1371/journal.pone.0175626)
Supplement: S1 Protocol — (DOCX) [file pone.0175626.s001.docx]

Pilot clinical study of the effect of lubiprostone on intestinal permeability in healthy volunteers

Study Protocol

Department of Gastroenterology and Hepatology Yokohama City University

Atsushi Nakajima, Professor

Protocol No.: LUBIYCU15001

Ver. 0.1:Prepared on March 31, 2015

Summary of Study Protocol

| Study title | Pilot clinical study of the effect of lubiprostone on intestinal permeability in healthy volunteers |
| --- | --- |
| Objectives | To verify how much lubiprostone can suppress intestinal permeability when intestinal permeability is increased in healthy adult volunteers.  [Primary objective]  To evaluate permeability of the small intestine at the administration of lubiprostone using the lactulose/mannitol test.  [Secondary objective]  To evaluate endotoxin activity in blood and changes in the intestinal flora at the time of administration of lubiprostone. |
| Study design | Open-label, randomized, parallel-group comparison study |
| Target sample size | 35 volunteers |
| Subjects | Healthy adults who meet all of the following “inclusion criteria” and do not correspond to any of the following “exclusion criteria” will be targeted in this study.  [Inclusion criteria]   1. Men at age ≥20 years but <60 years when the consent was obtained 2. Persons who were determined to be healthy adults by the investigator/sub-investigator (hereinafter referred to as investigator, etc.) during medical interview at screening 3. Persons from whom the written consent was obtained and who can abide by the compliance rules while participating in this study, receive medical examination and tests prescribed in this protocol, and report symptoms, etc.   [Exclusion criteria]   1. Persons who have received NSAIDs within 3 months before obtaining the consent (excluding topical administration of NSAIDs) 2. Persons with history of food or drug allergy (diclofenac sodium, lubiprostone, etc.) or who are suspected of such allergy 3. Persons with current or history of serious cardiovascular/vascular, hematological, respiratory, liver, kidney, gastrointestinal, or neuropsychiatric disease 4. Persons who are taking proton pump inhibitors (PPIs), antibiotics, or intestinal drugs (excluding topical administration of antibiotics) 5. Persons who have participated in other clinical studies and received the study drug, etc. within 1 month prior to the start of this study (calculated from the administration date of the study drug) 6. In the case of students at this university, those who are currently taking part in the studies conducted the Department of Gastroenterology and Hepatology 7. Persons who were judged by the investigator, etc. to be inappropriate as a subject for this study |
| Study drugs | Voltaren^®^ Tablets 25 mg (containing 25 mg of diclofenac sodium in 1 tablet)  Amitiza^®^ Capsules 24 µg (containing 24 µg of lubiprostone in 1 capsule) |
| Study procedures | [Acquisition of consent]   1. The investigator, etc. will explain the contents of this study using the information document and obtain the written consent. 2. After obtaining the consent, the investigator, etc. will perform medical interview, body measurements, etc. to judge the appropriateness of the subject’s participation. 3. If the subject was judged to be eligible for participation, the subject will be registered. Also, the lactulose/mannitol test, measurement of endotoxin activity in blood, and intestinal flora test will be performed.   [Increase of intestinal permeability]   1. Administer diclofenac sodium (75 mg/day) three times a day after every meal for 7 days. 2. After administering diclofenac sodium for 7 days, the investigator, etc. will perform medical examination and allocate the subjects who can move to the study period to either a study drug treatment group or a control group. 3. The lactulose/mannitol test, measurement of endotoxin activity in blood, and intestinal flora test will be performed.   Regarding the study period, for the first 6 subjects (3 subjects in each group) after the start of the study, the study treatment period or non-treatment period will be 28 days in this study. For the 7th subject and after that, the study treatment period or non-treatment period will be determined to be either 14 days or 28 days based on the results of the 6 subjects. If there is no difference in the 14-day treatment between the two groups but it is judged that there may be a tendency of difference in the 28-day treatment between the two groups, the study treatment period will be determined to be 28 days for the 7th subject and after that. In other cases, the study treatment period will be determined to be 14 days for the 7th subject and after that.  [Up to the first 6 subjects]  [Study period]  (In the case of the study treatment group)   1. Administer 1 capsule of the study drug once a day after lunch for 14 days. The subjects are instructed to comply with the “Rules to be followed by subjects”. 2. After administering the study drug for 14 days, the investigator, etc. will perform medical examination, as well as lactulose/mannitol test, measurement of endotoxin activity in blood, and intestinal flora test. 3. Administer 1 capsule of the study drug once a day after lunch for 14 days. 4. After administering the study drug for 14 days, the investigator, etc. will perform medical examination, as well as lactulose/mannitol test, measurement of endotoxin activity in blood, and intestinal flora test.   (In the case of the control group)   1. The study drug will not be administered (for 14 days). The subjects are instructed to comply with the “Rules to be followed by subjects”. 2. After 14 days, the investigator, etc. will perform medical examination, as well as lactulose/mannitol test, measurement of endotoxin activity in blood, and intestinal flora test. 3. The study drug will not be administered (for 14 days). 4. After 14 days, the investigator, etc. will perform medical examination, as well as lactulose/mannitol test, measurement of endotoxin activity in blood, and intestinal flora test.   [After the 7th subject]  [Study period]  (In the case of the study treatment group)   1. Administer 1 capsule of the study drug once a day after lunch for 14 days or 28 days. The subjects are instructed to comply with the “Rules to be followed by subjects”. 2. After administering the study drug for 14 days or 28 days, the investigator, etc. will perform medical examination, as well as lactulose/mannitol test, measurement of endotoxin activity in blood, and intestinal flora test.   (In the case of the control group)   1. The study drug will not be administered (for 14 days or 28 days). The subjects are instructed to comply with the “Rules to be followed by subjects”. 2. After administering the study drug for 14 days or 28 days, the investigator, etc. will perform medical examination, as well as lactulose/mannitol test, measurement of endotoxin activity in blood, and intestinal flora test. |
| Discontinuation criteria for each subject | The study will be discontinued for the subject who meets any of the following criteria.   1. The subject himself offered to withdraw from the study participation 2. The subject who could not comply with the prescribed administration of diclofenac sodium and study drug 3. The subject for whom the prescribed test was not performed 4. The subject who was judged impossible to move to the study period by the investigator, etc. 5. The subject who turned out that he did not meet the inclusion criteria or violated the exclusion criteria after the start of the study 6. The subject for whom the investigator, etc. judged that the study should be discontinued |
| Endpoints | [Efficacy endpoints]  Primary endpoint: Permeability of the small intestine using the lactulose/mannitol test  Secondary endpoints: endotoxin activity in blood, changes in intestinal flora  [Safety endpoints]  Adverse events, adverse reactions |
| Scheduled study period | May 2015 - March 2016 |

1. Background of this study

The results of the basic experiments suggest the correlation between small-intestinal mucosal injury and increase in the intestinal permeability and also the relationship with another disease recognized as Leaky Gut Syndrome.

Lubiprostone (brand name: Amitiza^®^ Capsules 24 µg (hereinafter referred to as the study drug) is a drug approved in November 2012 with “chronic constipation” as an indication. Since it is a prostaglandin derivative, it is reported from the basic experiments that the drug may prevent the onset of small-intestinal mucosal injury and increase in the intestinal permeability.

In this study, the effect of this study drug to contribute to the suppression of intestinal permeability will be examined by comparing the subjects in the study drug treatment group with those in the non-treatment group.

1. Objectives
   1. Primary objective

To evaluate permeability of the small intestine using the lactulose/mannitol test.

- 1. Secondary objectives

To evaluate endotoxin activity in blood.

To evaluate changes in the intestinal flora.

1. Subjects

The healthy adults who meet all of the following “3.1 Inclusion criteria” and do not correspond to any of the following “3.2 Exclusion criteria” will be the subjects in this study.

- 1. Inclusion criteria
     - 1. Men at age ≥20 years but <60 years when the consent was obtained
       2. Persons who were determined to be healthy adults by the investigator/sub-investigator (hereinafter referred to as investigator, etc.) during medical interview at screening
       3. Persons from whom the written consent was obtained and who can abide by the compliance rules while participating in this study, receive medical examination and tests prescribed in this protocol, and report symptoms, etc.
  2. Exclusion criteria
     - 1. Persons who have received NSAIDs within 3 months before obtaining the consent (excluding topical administration of NSAIDs)
       2. Persons with history of food or drug allergy (diclofenac sodium, lubiprostone, etc.) or who are suspected of such allergy
       3. Persons with current or history of serious cardiovascular/vascular, hematological, respiratory, liver, kidney, gastrointestinal, or neuropsychiatric disease
       4. Persons who are taking proton pump inhibitors (PPIs), antibiotics, or intestinal drugs (excluding topical administration of antibiotics)
       5. Persons who have participated in other clinical studies and received the study drug, etc. within 1 month prior to the start of this study (calculated from the administration date of the study drug)
       6. In the case of students at this university, those who are currently taking part in the studies conducted the Department of Gastroenterology and Hepatology
       7. Persons who were judged by the investigator, etc. to be inappropriate as a subject for this study

1. Explanation to subjects and acquisition of consent
   1. Preparation of the informed consent form

The informed consent form which is used to obtain the consent to participate in this study from a subject will be prepared by the principal investigator and must be approved by the Ethics Committee of the clinical study site.

- 1. Revision of the informed consent form

The principal investigator will promptly revise the informed consent form if he or she determines it necessary to revise the informed consent form such as the case when new important information which may be related to the subject’s consent is obtained and obtain the approval of the Ethics Committee.

- 1. Timing and method of obtaining the consent

4.3.1　At the time of registration

The written consent should be obtained from the subject prior to his or her participation in this study (prior to the administration of the study drug) according to the following procedures.

- - - 1. The investigator, etc. should provide adequate explanation about this study to the subject who may be considered possible to participate in this study by using the informed consent form.
      2. The investigator, etc. should give the subject an opportunity to ask questions and enough time to make a judgment on his or her participation in this study before obtaining the consent.
      3. The investigator, etc. should give satisfactory answers to all questions raised by the subject.
      4. If the subject agrees to participate in this study, the investigator, etc. who provided the explanation should ask the subject to sign the informed consent form or affix his or her printed name and seal on the form and enter the date.
      5. The investigator, etc. should give the subject a copy of the informed consent form and the information document before he or she participates in the study. Also, the investigator, etc. should retain the original informed consent form at the clinical study site.

4.3.2　　If any information that may affect the subject’s intention is obtained

If any information that may affect the subject’s intention to continue to participate in the study (e.g., safety information) is obtained, the investigator, etc. should confirm the subject’s intention to continue to participate in the study or not and record it in writing along with the date that confirmed that effect.

4.3.3　　At revision of the informed consent form

If the informed consent form is revised, the investigator, etc. should obtain the subject’s consent again.

1. Study drugs

Voltaren^®^ Tablets 25 mg (containing 25 mg of diclofenac sodium in 1 tablet)

Amitiza^®^ Capsules 24 µg (containing 24 µg of lubiprostone in 1 capsule)

1. Study design
   1. Study design

Open-label, randomized, parallel-group comparison study

For the first 6 subjects (3 subjects in each group) after the start of the study, the study treatment period or non-treatment period will be 28 days in this study. For the 7th subject and after that, the study treatment period or non-treatment period will be determined to be either 14 days or 28 days based on the results of the 6 subjects. If there is no difference in the 14-day treatment between the two groups but it is judged that there may be a tendency of difference in the 28-day treatment between the two groups, the study treatment period will be determined to be 28 days for the 7th subject and after that. In other cases, the study treatment period will be determined to be 14 days for the 7th subject and after that.

- 1. Dosage and administration of the study drug

Subjects will orally receive one capsule of Amitiza once daily after lunch for 14 days or 28 days.

- 1. Study registration

**This study will be registered to the UMIN Clinical Trials Registry and the information on the study will be made public. The clinical study registration will be done before registration of the first subject.**

- 1. Target sample size

35 subjects

- 1. Flow chart

*1: Lactulose/mannitol test, measurement of endotoxin activity in blood, intestinal flora test

*2: For the 7th subject and after that, the study treatment period (non-treatment period) will be determined to be either 14 days or 28 days based on the results of the first 6 subjects (3 subjects in each group).

Visit (1)

Visit (2)

Visit (3)

Study drug treatment group

Control group

Explain and obtain the consent

Medical examination, physical findings, etc.

Case registration

Administer diclofenac

Allocation

Tests for intestinal permeability^*1^

Tests for intestinal permeability^*1^

Administer the study drug

Not administer

←14 or 28 days*^2^→

7 days

←14 or 28 days*^2^→

Tests for intestinal permeability^*1^

Up to the first 6 subjects

Visit (1)

Visit (2)

Visit (3)

Visit (4)

Study drug treatment group

Control group

Explain and obtain the consent

Medical examination, physical findings, etc.

Case registration

Tests for intestinal permeability^*1^

Administer diclofenac

Allocation

Tests for intestinal permeability^*1^

Administer the study drug

Not administer

Tests for intestinal permeability^*1^

Tests for intestinal permeability^*1^

Administer the study drug

Not administer

14 days

14 days

14 days

14 days

7 days

*1: Lactulose/mannitol test, measurement of endotoxin activity in blood, intestinal flora test

After 7th subject

- 1. Method of allocation

Until the 6th subject, block randomization is conducted using a block of block size 6. For the 7th subject and after that, block randomization is conducted using a block of block size 4.

- 1. Prohibited concomitant medications and therapies

After obtaining the consent until the completion of the study period, the use of proton pump inhibitors (PPIs), antibiotics, and intestinal drugs will be prohibited. However, the use of antibiotics will be allowed only for a local therapeutic purpose.

If the prohibited medication is used for inevitable reasons such as the treatment of adverse events, the investigator, etc. should describe the drug name, duration of use, intended use, etc. in the case report form.

1. Study implementation period

May 2015 - March 2016

1. Study method

This study will be implemented according to the following procedures and schedules.

- 1. Recruitment of subjects

The outline of this study will be posted on a bulletin board, etc. in the university to recruit subjects.

- 1. Study procedures

For the first 6 subjects (3 subjects in each group), the study treatment period or non-treatment period will be 28 days in this study. For the 7th subject and after that, the study treatment period or non-treatment period will be determined to be either 14 days or 28 days based on the results of the 6 subjects.

[Acquisition of consent]

- - - 1. The investigator, etc. will explain the contents of this study using the information document and obtain the written consent.
      2. After obtaining the consent, the investigator, etc. will perform medical interview, body measurements, etc. to judge the appropriateness of the subject’s participation.
      3. If the subject was judged to be eligible for participation, the subject will be registered. Also, the lactulose/mannitol test, measurement of endotoxin activity in blood, and intestinal flora test will be performed.

[Increase of intestinal permeability]

- - - 1. Administer diclofenac sodium (75 mg/day) three times a day after every meal for 7 days.
      2. After administering diclofenac sodium for 7 days, the investigator, etc. will perform medical examination and allocate the subjects who can move to the study period to either a study drug treatment group or a control group.
      3. The lactulose/mannitol test, measurement of endotoxin activity in blood, and intestinal flora test will be performed.

For the first 6 subjects (3 subjects in each group) after the start of the study, the study treatment period or non-treatment period will be 28 days. For the 7th subject and after that, the study treatment period or non-treatment period will be determined to be either 14 days or 28 days based on the results of the 6 subjects.

[Up to the first 6 subjects]

[Study period]

(In the case of the study treatment group)

- - - 1. Administer 1 capsule of the study drug once a day after lunch for 14 days. The subjects are instructed to comply with the “Rules to be followed by subjects”.
      2. After administering the study drug for 14 days, the investigator, etc. will perform medical examination, as well as lactulose/mannitol test, measurement of endotoxin activity in blood, and intestinal flora test.
      3. Administer 1 capsule of the study drug once a day after lunch for 14 days.
      4. After administering the study drug for 14 days, the investigator, etc. will perform medical examination, as well as lactulose/mannitol test, measurement of endotoxin activity in blood, and intestinal flora test.

(In the case of the control group)

- - - 1. The study drug will not be administered (for 14 days). The subjects are instructed to comply with the “Rules to be followed by subjects”.
      2. After 14 days, the investigator, etc. will perform medical examination, as well as lactulose/mannitol test, measurement of endotoxin activity in blood, and intestinal flora test.
      3. The study drug will not be administered (for 14 days).
      4. After 14 days, the investigator, etc. will perform medical examination, as well as lactulose/mannitol test, measurement of endotoxin activity in blood, and intestinal flora test.

[After the 7th subject]

[Study period]

(In the case of the study treatment group)

- - - 1. Administer 1 capsule of the study drug once a day after lunch for 14 days or 28 days. The subjects are instructed to comply with the “Rules to be followed by subjects”.
      2. After administering the study drug for 14 days or 28 days, the investigator, etc. will perform medical examination, as well as lactulose/mannitol test, measurement of endotoxin activity in blood, and intestinal flora test.

(In the case of the control group)

- - - 1. The study drug will not be administered (for 14 days or 28 days). The subjects are instructed to comply with the “Rules to be followed by subjects”.
      2. After administering the study drug for 14 days or 28 days, the investigator, etc. will perform medical examination, as well as lactulose/mannitol test, measurement of endotoxin activity in blood, and intestinal flora test.
  1. Study schedule

Screening period

Period for treatment with diclofenac

Period for treatment with study drug

At the time of discon-
tinuation

Informed consent

Medical examinations

Physical measurements (height/weight)

Vital signs

Enrollment

Allocation

Administer diclofenac

Confirmation of concomitant medications

Identification of adverse events

Administer the study drug

Lactulose/mannitol test

Serum endotoxin test

Intestinal flora test

Lactulose/mannitol test

Serum endotoxin test

Intestinal flora test

Not administer

All subjects

Study drug treatment group

Control group

⭘ Essential ◇ To be performed as far as possible

After 7th subject

Screening period

Period for treatment with diclofenac

Period for treatment with study drug

At the time of discontinuation

Informed consent

Medical examinations

Physical measurements (height/weight)

Vital signs

Enrollment

Allocation

Administer diclofenac

Confirmation of concomitant medications

Identification of adverse events

Administer the study drug

Lactulose/mannitol test

Serum endotoxin test

Intestinal flora test

Lactulose/mannitol test

Serum endotoxin test

Intestinal flora test

Not administer

All subjects

Study drug treatment group

Control group

Day (in the case of 14 days)^1)^

Day (in the case of 28 days)^1)^

⭘ Essential  ◇ To be performed as far as possible

1) Based on the results observed up to the 6th subject, a 14 day or 28 day treatment period of study drug (or non-treatment period) would be selected for the 7th subject and after that.

Up to the first 6 subjects

- 1. Matters to be followed by subjects

During the study period, the investigator, etc. or provides subjects with the following instructions. This study will be implemented according to the following procedures and schedules.

- - - 1. Subjects take diclofenac sodium and study drug according to the instructions given by the investigator, etc.
      2. If a subject missed (could not) to take study drug after lunch, he/she takes it after supper. If he/she missed (could not) to take study drug after supper, he/she takes no study drug on that day.
      3. During the study period, combined treatments [medications (including supplements) and therapies] are prohibited, unless the investigator, etc. considers that use of combined treatment is necessary for treating adverse event(s) that occurred in a subject(s) during the course of the study.
      4. If a subject experienced any abnormality during the study period, he/she must inform the investigator, etc. of it.
      5. Any subject must not drink a large of alcohol during the study period.
      6. It is not allowed to take any food or drink from 21:00 onward on the day before and the day when lactulose/mannitol test is performed. However, a small amount of drink without sugar content is allowed.

1. Items and time points for investigations/examinations
   1. Patient characteristics

Investigation items: date of birth, gender, height, weight, complication, past medical history, history of allergy, concomitant drug, frequency of bowel movement, stool condition, with or without use of purgative

Target groups: study drug group, control group

Time points of investigation: Screening period

- 1. Vital signs

Investigation items: blood pressure, pulse rate

Target groups: study drug group, control group

Time points of investigation: screening period, at the time when an adverse event occurred, at the time of discontinuation

- 1. Physical findings

Investigation items: subjective or objective findings, adverse events, nausea, frequency of bowel movement, stool conditions

Target groups: study drug group, control group
Time points of investigation: screening period, period for treatment with diclofenac, period for treatment with study drug, at the time of discontinuation

- 1. Compliance to diclofenac

Investigation items: treatment period, daily dose, cessation/discontinuation of medication, reason of cessation/discontinuation of medication, reason for change

Target groups: study drug group, control group

Time points of investigation: Period for treatment with diclofenac

- 1. Compliance to study drug

Investigation items: treatment period, daily dose, cessation/discontinuation of medication, reason of cessation/discontinuation of medication, reason for change

Target groups: Study drug treatment group

Time points of investigation: Period for treatment with study drug

- 1. Lactulose/mannitol test

Target groups: study drug group, control group

Time points of investigation: screening period, period for treatment with diclofenac, period for treatment with study drug, at the time of discontinuation

- 1. Endotoxin activity in blood

Target groups: study drug group, control group

Time points of investigation: screening period, period for treatment with diclofenac, period for treatment with study drug, at the time of discontinuation

Volume of blood to be collected: 2 mL in each time

- 1. Intestinal flora

Target groups: study drug group, control group

Time points of investigation: screening period, period for treatment with diclofenac, period for treatment with study drug, at the time of discontinuation

1. Endpoints
   1. Efficacy endpoints

Primary endpoint: Permeability of the small intestine using the lactulose/mannitol test

Secondary endpoints: endotoxin activity in blood, changes in intestinal flora

- 1. Safety endpoints

Adverse events, adverse reactions

1. Adverse events
   1. Adverse events

An adverse event is defined as any untoward medical occurrence (including abnormal laboratory findings) in a subject and which does not necessarily have to have a causal relationship with study drug. In this study, adverse events will be collected during the period from the start of treatment with diclofenac sodium to the end of treatment with study drug.

Adverse events will be assessed based on the clinical symptoms observed in subjects.

All adverse events will be documented by the investigator, etc. on the case report form to record the dates of its occurrence and resolution, its seriousness and severity, whether the subject was treated or not, its outcomes and the results of evaluation of causal relationship between the event and study drug.

- 1. Serious adverse events

A serious adverse event includes those described below.

- - - 1. Death
      2. Any adverse event which may lead to death
      3. Any adverse event which requires admission to a hospital or clinic, or prolongation of existing hospitalization (excluding hospitalization for routine tests)
      4. Disability
      5. Any adverse event which may result in disability
      6. Any adverse event which is serious pursuant to 1)-5)
      7. Any congenital disease or anomaly in later generations.
  1. Reporting and handling of adverse events

When the investigator, etc. learned occurrence of any adverse event in a subject, he or she would take adequate medical actions to ensure safety of the subject, above everything else, within the treatment covered by health insurance (e.g., endoscopic examination, treatment with medication). In addition, the investigator, etc. will inform MR of Abbott Japan Co., Ltd. who is in charge of the study site on all the adverse events (serious and non-serious) caused by lubiprostone, immediately after those events occurred. Furthermore, if any serious adverse event occurred during the course of this study, the investigator, etc. will inform the head of the study site also on the event.

- 1. Causal relationship between an adverse event and study drug

Assessment results of causal relationship between an adverse event and study drug would be classified as follows:

- - - 1. Related
         Temporal relationship is observed clearly and causes other than study drug can be excluded
      2. Unrelated
         No temporal relationship is observed or the event can be explained by other causes.
  1. Intensity (severity) of adverse event

The intensity of the adverse event would be judged based on the following:

- - - 1. Mild
         The adverse event causes no interference with usual activities of the subject. He does not require treatment for any condition.
      2. Moderate
         The adverse event causes some interference with usual activities of the subject. Treatment for symptoms associated with the event or premature discontinuation of the study (except for premature discontinuation due to his request) is required.
      3. Severe
         The adverse event causes inability to perform usual activities of the subject. Premature discontinuation of the study (except for premature discontinuation due to his request) and treatment of the subject are required.
  1. Definitions of adverse reactions

When causal relationship between an adverse event and study drug is not deniable (1. definitely related, 2. probably related, 3. possibly related), the event is considered as an adverse reaction.
An unexpected adverse reaction is that which is not listed in the Interview Form of study drug (2014) or that which is listed in the Interview Form but its nature or severity is different from that described in the Interview Form.

- 1. Expected adverse reactions of study drug

Expected adverse reactions of Voltaren include the following events [based on the Interview Form of study drug (2015)].

Among the 1,474 subjects investigated until the approval, 239 adverse reactions were observed in 160 subjects (10.85%). Gastrointestinal symptoms such as gastric distress occurred in 139 subjects (9.43%) as major symptoms. Other symptoms included edema observed in 14 subjects (0.95%) and skin symptoms such as rash in 23 subjects (1.56%).

In the post-marketing drug-use result survey, 4,545 adverse reactions were observed in 2,749 (7.71%) out of the 35,653 subjects. As symptoms, gastrointestinal symptoms were observed in 2,365 subjects (6.63%) followed by general symptoms such as edema in 215 subjects (0.60%) and skin symptoms in 172 subjects (0.48%).

Reports of gastric ulcer and liver disorder as other adverse reactions accounted for below 0.1%.

Expected adverse reactions of Amitiza include the following events [based on the Interview Form of study drug (2014)].

Adverse reactions including abnormal laboratory findings were found in 196 (62%) /315 subjects who received study drug at a dose of 48 μg/day (24 μg b.i.d.) in 3 Japanese clinical studies. Most commonly observed adverse reactions included diarrhea in 95 subjects (30%) and nausea in 73 subjects (23%).

1. Study termination criteria in subjects

The study will be discontinued for the subject who meets any of the following criteria.

- - - 1. The subject himself offered to withdraw from the study participation
      2. The subject who could not comply with the prescribed administration of diclofenac sodium and study drug
      3. The subject for whom the prescribed test was not performed
      4. The subject who was judged impossible to move to the study period by the investigator, etc.
      5. The subject who turned out that he did not meet the inclusion criteria or violated the exclusion criteria after the start of the study
      6. The subject who developed an adverse reaction that falls under the category of Grade 2 or higher adverse reaction according to CTCAC v4.0 (moderate or severe prescribed in Section 11.6)
      7. The subject for whom the investigator, etc. judged that the study should be discontinued

If this study is discontinued, the test after discontinuation will not be conducted as a rule unless the subject requests such test.

1. Compensation for research-related health injuries

In the event of research-related health injury, the study site must take necessary and adequate measures including medical treatments for the injury. On this occasion, if the principal investigator considers that the health injury is associated with the proper use of study drug and that there is causal relationship between the injury and study drug, the investigator is responsible for compensation. However, medical care cost and medical allowance will not be covered. If the health injury is considered attributable to the subject’s intention or serious mistake, the compensation cost may not be covered.

The principal investigator take out insurance designating the investigator and study site as persons insured, in order to take measures for liability of compensation of health-related injury associate with study drug.

1. Ethical and scientific conduct of the study
   1. Compliance with the declaration of Helsinki

The study will be conducted according to the declaration of Helsinki, ethical guidelines of clinical studies and the clinical protocol.

- 1. Ethical committee
     - 1. Prior to conducting the study, the principal investigator will be judged by ethical committee of the study site, and performs the study after approved by the committee.
       2. When provided with new information relating to protocol amendment or safety, the principal investigator requests of the ethical committee to review whether the study can be continued or not.
       3. In order to undergo continued review by ethical committee, the principal investigator reports in writing the status of implementation of the study to the head of study site once a year or according to the procedures of the study site.

1. Approval of, compliance with and amendment of study protocol
   1. Approval of study protocol

Prior to the conduct of the study, the principal investigator will obtain an approval of the contents of study protocol from the head of the study site.

- 1. Compliance with clinical protocol

The principal investigator will not make any deviation from or amendment of study protocol without prior written approval from ethical committee, unless necessary for avoiding the immediate risk of a subject. In that case, the investigator, etc. will report on the deviation or amendment to ethics committee and head of the study site.

- 1. Amendment of study protocol

The principal investigator will not make any amendment of study protocol without prior written approval from ethical committee.

1. Completion, premature or temporal discontinuation of the study
   1. Completion of the study

When the study is completed, the principal investigator should inform the head of the study site of the completion.

- 1. Premature or temporal discontinuation of the study

The principal investigator discontinues prematurely or temporarily the study in the event of the following cases.

- - - 1. Unavoidable ethical or medical situations occurred including ensuring safety of a subject.
      2. Scientific validity was lost to conduct the study.
      3. Study policy was changed.
      4. Major or continued non-compliance by the investigator, etc. or study site was found.
      5. Ethical committee made a decision to discontinue the study prematurely or temporarily during the continued review of the on-going study.
      6. The study cannot be continued for some reasons, for example, transfer of the investigator.
      7. There is no prospect for subjects who meet the inclusion criteria.
      8. The investigator made a judgment decision to discontinue the study prematurely or temporarily.

1. Preparation of case report form

Case report form will be prepared for subjects who gave informed consent.

1. Quality control of the study

The principal investigator will designate a monitor who performs quality control of the study. The monitor confirms that the study is conducted in compliance with the study protocol and ethical guideline for clinical study.

1. Quality assurance of the study

The principal investigator will designate a person in charge of audit who conducts quality assurance of the study. The person in charge of audit confirms that the study is conducted in compliance with the study protocol and ethical guideline for clinical study.

1. Protection of human rights of subjects
   1. Person in charge of personal data management

The principal investigator will designate a person in charge of personal data management pursuant to the rules of the study site.

- 1. Handling of personal data

For enrollment of a subject and preparation of his case report form, the investigator, etc. identify him by the subject identification code to retain the information as linkable anonymized data. Thus, personal data of a subject must be handled with caution.

1. Storage and destruction of records
   1. Storage of records

The investigator, etc. retains following records and documents for 5 years: case report forms, laboratory data, documents of ethical committee, documents on subject’s consent, documents on treatment with study drug and the study-related data.

- 1. Destruction of records

After completion of the storage period of records, the study-related data will be destroyed adequately, not to leak such secrets as personal data of subjects.

1. Statistical analysis

In this study, statistical analysis will be performed mainly for the following items. Details of the statistical design will be described in the statistical analysis plan.

- 1. Demographic characteristics

In addition to summary of subjects, summary statistics of demographic variables (including gender and age) of subjects will be shown.

- 1. Analysis set

Analysis set of the study will be Full analysis set (FAS) consisting of all subjects enrolled in the study, except for those described below.

• Subjects who were found to be ineligible for the study after the registration

• Subjects who took no study drug

• Subjects who had no efficacy data

- 1. Analysis methods for efficacy endpoints
     - 1. Primary endpoint

The summary statistics will be calculated for the permeability of the small intestine at baseline and after treatment respectively (mean, median).

For the primary analysis, (permeability of the small intestine after treatment - permeability of the small intestine at baseline) will be sought for each subject and the corresponding Wilcoxon test will be performed. The significance level will be 5% on both sides. For reference, the 2 groups will be compared using the Analysis of Covariance with permeability of the small intestine at baseline as a covariate.

- - - 1. Secondary endpoint

For endotoxin activities in blood and changes in the intestinal flora, too, (values after treatment - values at baseline) will be sought for each subject and the paired Wilcoxon test will be performed. The significance level will be 5% on both sides. For reference, the 2 groups will be compared using the Analysis of Covariance with baseline value as a covariate.

- 1. Analysis methods for safety endpoints

The type, severity, and incidence of adverse events and adverse reactions that occurred will be calculated and compared between the 2 groups.

- 1. Design of sample size

This study will be conducted for healthy volunteers as pilot positioning. Because the prior data do not exist, the scheduled number of registered subjects will be 30 subjects who are considered to be collectable within the specified study period.

1. Financing source and conflict of interest associated with the study

The study is sponsored by Abbott Japan Co., Ltd. and will be conducted by Yokohama City University as a contract research by Abbott Japan Co., Ltd.

Responsible person for study will declare any conflict of interest according to conflict of interest management rules of the study site.

The investigator will prepare the database to be used in this study by making it anonymous and Abbott Japan Co., Ltd. can neither access it nor perform statistical analysis using the database. Evaluation and interpretation of the results of the study will be performed by the investigator. The results of the study will not be disclosed unless the written consent is obtained according to the confidentiality rules.

1. Expenses on study participation

A research cooperation expenditure will be 50,000 yen per subject.

The investigator will purchase diclofenac sodium and study drug which will be used in the study.

1. Publication of the results

Whatever the study results may be, the principal investigator will publish the results in a scientific journal or in a scientific meeting.

Until 60 days prior to the publication of the study results, the investigator or the study site will submit the manuscript with the same contents to Abbott Japan Co., Ltd. to request their review and comments.

1. Study organization
   1. Responsible person for study (Investigator)

Department of Gastroenterology and Hepatology Yokohama City University Hospital
Professor   Atsushi Nakajima

- 1. Sub-investigator

Department of Gastroenterology and Hepatology Yokohama City University Hospital
Postgraduate     Takayuki Kato

Department of Gastroenterology and Hepatology Yokohama City University Hospital
Medical advisor   Takaomi Kessoku

Department of Gastroenterology and Hepatology Yokohama City University Hospital
Postgraduate    Yasushi Honda

Department of Biostatistics, Yokohama City University Graduate School of Medicine
Professor   Takeharu Yamanaka

- 1. Person in charge of personal data management

Department of Gastroenterology and Hepatology Yokohama City University Hospital
Associate Professor   Satoshi Saito

- 1. Responsible person for statistical analysis

Department of Biostatistics, Yokohama City University Graduate School of Medicine
Professor   Takeharu Yamanaka

- 1. Person in charge of monitoring

Department of Gastroenterology and Hepatology Yokohama City University Hospital
Associate Professor   Satoshi Saito

- 1. Person in charge of audit

Department of Gastroenterology and Hepatology Yokohama City University Hospital
Medical advisor   Shiori Uchiyama

1. References

[1] Nonsteroidal anti-inflammatory drug-induced visible and invisible small intestinal injury.
J Clin Biochem Nutr. 2013 Jul;53(1):55-9. doi: 10.3164/jcbn.12-116. Epub 2013 Apr 9.

[2] Lubiprostone Increases Spontaneous Bowel Movement Frequency and Quality of Life in Patients With Chronic Idiopathic Constipation.
[Clin Gastroenterol Hepatol.](http://www.ncbi.nlm.nih.gov/pubmed/?term=Lubiprostone+Increases+Spontaneous+Bowel+Movement+Frequency+and+Quality+of+Life+in+Patients+With+Chronic+Idiopathic+Constipation) 2014 Aug 24. pii: S1542-3565(14)01245-2

[3] Clinical trial: lubiprostone in patients with constipation associated irritable bowel syndrome
 – results of two randomized, placebo-controlled studies
Aliment Pharmacol Ther. 2009 Feb 1;29(3):329-41.
