# Supplementary material for: Lubiprostone improves intestinal permeability in humans, a novel therapy for the leaky gut: A prospective randomized pilot study in healthy volunteers
Source: PLoS One. 2017 Apr 14;12(4):e0175626. doi: 10.1371/journal.pone.0175626 (PMC5391961; doi:10.1371/journal.pone.0175626)
Supplement: S2 Protocol — (DOCX) [file pone.0175626.s002.docx]

健常ボランティアにおけるルビプロストンが

腸管透過性に与える効果のパイロット臨床試験

研究計画書

横浜市立大学　肝胆膵消化器病学

主任教授　中島　淳

研究計画書番号：A201501

0.1 版：作成日 2015年3月26日

研究計画書の要約

| 研究の名称 | 健常ボランティアにおけるルビプロストンによる腸管透過性に与える効果の検証 |
| --- | --- |
| 研究の目的 | 健康成人ボランティアにおいて腸管透過性を亢進させた際にルビプロストンがどの程度腸管透過性を抑制するかを検証する。  [主目的]  ルビプロストン投与時のラクツロース/マンニトール試験を用いた小腸腸管透過性を評価する。  [副次目的]  ルビプロストン投与時の血中エンドトキシン活性及び腸内細菌叢の変化を評価する。 |
| 研究デザイン | 非盲検無作為化並行群間比較試験 |
| 目標症例数 | 35名 |
| 対　　　象 | 以下の「選択基準」をすべて満たし、かつ「除外基準」のいずれにも該当しない健康成人を対象とする。  [選択基準]   1. 同意取得時の年齢が20歳以上60歳未満の男性 2. スクリーニング時の問診にて、研究責任／分担医師（以下、研究責任医師等）が健康成人と判断した者 3. 文書による同意が得られ、本研究参加中の遵守事項を守り、本研究計画書に定められた診察、検査を受け、症状などの申告ができる者   [除外基準]   1. 同意取得3ヶ月前以内にNSAIDを服用している者（局所投与は除く） 2. 食物や薬物（ジクロフェナクナトリウム、ルビプロストン等）アレルギーの既往又はそれが疑われる者 3. 重篤な心臓・血管系、血液系、呼吸器系、肝臓、腎臓、消化器系、神経精神疾患の現病歴及び既往歴のある者 4. プロトンポンプインヒビター、抗生物質、整腸剤を服用している者（抗生物質の局所投与は除く） 5. 本研究開始前1ヶ月以内（試験薬投与日より計算）に、他の臨床試験に参加し、試験薬等の投与を受けた者 6. 本学の学生の場合は、肝胆膵消化器病試験中である者 7. その他、研究責任医師等が本研究の被験者として不適当と判断した者 |
| 試験薬 | アミティーザ^Ⓡ^カプセル24µg（1カプセルにルビプロストン24µg含有） |
| 研究手順 | [同意取得]   1. 研究責任医師等は、説明文書を用いて本研究の内容を説明し、文書にて同意を取得する。 2. 同意取得後、研究責任医師等にて問診・身体測定等を行い、参加の適否を判断する。 3. 参加が可能と判断した場合は、被験者を登録する。また、ラクツロース/マンニトール試験、血中エンドトキシン活性測定及び腸内細菌叢検査を実施する。   ［腸管透過性の亢進］   1. ジクロフェナクナトリウム（75mg/day）を1日3回毎食後7日間投与する。 2. ジクロフェナクナトリウムを7日間投与後、診察を行い、試験期に移行可能な被験者を試験薬投与群または対照群に割り付ける。 3. ラクツロース/マンニトール試験、血中エンドトキシン活性測定及び腸内細菌叢検査を実施する。   試験期について、研究開始最初の6例（各群3例）は、試験薬投与または非投与期間を28日間とする。7例目以降は、6例までの結果に基づき、試験薬投与または非投与期間を14日間又は28日間のいずれかに決定する。  【最初の6例まで】  [試験期]  （試験薬投与群の場合）   1. 試験薬1カプセルを1日1回昼食後に14日間投与する。被験者には「被験者が遵守すべき事項」に従い生活してもらう。 2. 試験薬を14日間投与後、診察を行い、ラクツロース/マンニトール試験、血中エンドトキシン活性測定及び腸内細菌叢検査を実施する。 3. 試験薬1カプセルを1日1回昼食後に14日間投与する。 4. 試験薬を14日間投与後、診察を行い、ラクツロース/マンニトール試験、血中エンドトキシン活性測定及び腸内細菌叢検査を実施する。   （対照群の場合）   1. 試験薬は投与しない（14日間）。被験者には「被験者が遵守すべき事項」に従い生活してもらう。 2. 14日後、診察を行い、ラクツロース/マンニトール試験、血中エンドトキシン活性測定及び腸内細菌叢検査を実施する。 3. 試験薬は投与しない（14日間）。 4. 14日後、診察を行い、ラクツロース/マンニトール試験、血中エンドトキシン活性測定及び腸内細菌叢検査を実施する。   【7例目以降】  [試験期]  （試験薬投与群の場合）   1. 試験薬1カプセルを1日1回昼食後に14日間又は28日間投与する。被験者には「被験者が遵守すべき事項」に従い生活してもらう。 2. 試験薬を14日間又は28日間投与後、診察を行い、ラクツロース/マンニトール試験、血中エンドトキシン活性測定及び腸内細菌叢検査を実施する。   （対照群の場合）   1. 試験薬は投与しない（14日間又は28日間）。被験者には「被験者が遵守すべき事項」に従い生活してもらう。 2. 14日又は28日後、診察を行い、ラクツロース/マンニトール試験、血中エンドトキシン活性測定及び腸内細菌叢検査を実施する。 |

| 個々の被験者の  研究中止基準 | 次のいずれかに該当する被験者は、本研究を中止する。   1. 被験者自身が研究参加の撤回を申し出た者 2. 規定されたジクロフェナクナトリウム及び試験薬の服用ができなかった者 3. 規定された検査ができなかった者 4. 試験期への移行ができないと研究責任医師等が判断した者 5. 研究開始後に選択基準に合致していないこと又は除外基準に抵触することが判明した者 6. その他、研究責任医師等が研究を中止するべきと判断した者 |
| --- | --- |
| 評価項目 | [有効性評価項目]  　主要評価項目：ラクツロース/マンニトール試験を用いた小腸腸管透過性  　副次評価項目：血中エンドトキシン活性、腸内細菌叢の変化  [安全性評価項目]  有害事象、副作用 |
| 予定研究期間 | 2015年4月1日～2016年3月31日 |

1. 本研究の背景

基礎実験の結果から小腸粘膜傷害は腸管透過性の亢進と相関しており、他のLeaky Gut Syndromeとして認識されている疾患との関連も示唆されている。

ルビプロストン（販売名：アミティーザ^®^カプセル24µg (以下、試験薬)）は、2012年11月に「慢性便秘症」を適応として承認された薬剤であるが、プロスタグランジン誘導体であり、小腸傷害及び腸管透過性の亢進を予防する可能性は基礎実験でも報告されている。

そこで、本研究では、試験薬が腸管透過性の抑制に寄与する効果を試験薬投与群と非投与群を比較し、検討する。

1. 研究の目的
   1. 主目的

ラクツロース/マンニトール試験を用いた小腸腸管透過性を評価する。

- 1. 副次的目的

血中エンドトキシン活性を評価する。

腸内細菌叢の変化を評価する。

1. 対象

以下の「3.1.選択基準」をすべて満たし、かつ「3.2.除外基準」のいずれにも該当しない健康成人を対象とする。

- 1. 選択基準

1. 同意取得時の年齢が20歳以上60歳未満の男
2. スクリーニング時の問診にて、研究責任／分担医師（以下、研究責任医師等）が健康成人と判断した者
3. 文書による同意が得られ、本研究参加中の遵守事項を守り、本研究計画書に定められた診察、検査を受け、症状などの申告ができる者
   1. 除外基準
4. 同意取得3ヶ月前以内にNSAIDを服用している者（局所投与は除く）
5. 食物や薬物（ジクロフェナクナトリウム、ルビプロストン等）アレルギーの既往又はそれが疑われる者
6. 重篤な心臓・血管系、血液系、呼吸器系、肝臓、腎臓、消化器系、神経精神疾患の現病歴及び既往歴のある者
7. プロトンポンプインヒビター、抗生物質、整腸剤を服用している者（抗生物質の局所投与は除く）
8. 本研究開始前1ヶ月以内（試験薬投与日より計算）に、他の臨床試験に参加し、試験薬等の投与を受けた者
9. 本学の学生の場合は、肝胆膵消化器病試験中である者
10. その他、研究責任医師等が本研究の被験者として不適当と判断した者
11. 被験者に対する説明と同意取得
    1. 説明文書及び同意文書の作成

被験者から本研究への参加の同意を得るために用いる説明文書及び同意文書は、研究責任医師が作成し、臨床研究機関の倫理委員会において承認を受けたものを使用する。

- 1. 説明文書及び同意文書の改訂

研究責任医師は、被験者の同意に関連しうる新たな重要な情報を入手した場合など説明文書及び同意文書を改訂する必要があると認めた時には、すみやかに説明文書及び同意文書の改訂を行い、倫理委員会の承認を得る。

- 1. 同意取得の時期と方法
     1. 登録時

研究参加前（試験薬投与前）に下記の手順により、被験者の同意を文書により得る。

1. 研究責任医師等は、研究へ参加可能と考えられる被験者に対し、説明文書及び同意文書を用いて十分に説明を行う。
2. 研究責任医師等は、同意を得る前に被験者が質問する機会と、研究に参加するか否かを判断するのに十分な時間を与える。
3. 研究責任医師等は、被験者からのすべての質問事項に対して、被験者が満足するような回答を示す。
4. 被験者が研究に参加することを同意した場合、説明を行った研究責任医師等及び被験者は同意文書に記名捺印又は署名し、日付を記入する。
5. 研究責任医師等は、被験者が研究に参加する前に、同意文書の写し及び説明文書を被験者に手渡す。また、研究責任医師等は臨床研究機関において同意文書の原本を保存する。
   - 1. 被験者の意思に影響を与える可能性のある情報が得られた場合

研究参加の継続に関して、被験者の意思に影響を与える可能性のある情報（安全性に関する情報等）が得られた場合、研究責任医師等は、当該情報を被験者に伝え、研究に継続して参加するか否かについて、被験者の意思を確認し、その旨を確認した日付とともに文書にて記録する。

- - 1. 説明文書及び同意文書の改訂時

説明文書及び同意文書を改訂した場合、研究責任医師等は、再度、被験者の同意を取得する。

1. 試験薬

ボルタレン^Ⓡ^錠 25mg(1錠にジクロフェナクナトリウム25mg含有)

アミティーザ^Ⓡ^カプセル24µg（1カプセルにルビプロストン24µg含有）

1. 研究デザイン
   1. 研究デザイン

非盲検無作為化並行群間比較試験

なお、本試験では、研究開始最初の6例（各群3例）は、試験薬投与または非投与期間を28日間とし、その結果に基づき、7例目以降の試験薬投与または非投与期間を14日間とするか28日間とするかを決定する。14日間の投与で両群に差がなく、かつ28日間の投与では両群に差が出そうな傾向であると判断した場合は7例目以降を28日間投与とする。それ以外の場合は7例目以降は14日間の投与とする。

- 1. 試験薬の用法・用量

1日1回1カプセルを昼食後14日又は28日間内服する。

- 1. 試験登録

本研究は、UMIN臨床試験登録システムに登録し、情報公開する。臨床試験登録は、最初の症例の登録前に行う

- 1. 目標症例数

35例

- 1. フローチャート

- 1. 割付の方法

6例目まではブロックサイズ6のブロックを用いて、ブロックランダム化を実施する。7例目以降は、ブロックサイズ4のブロックを用いて、ブロックランダム化を実施する。

- 1. 併用禁止薬剤及び療法

同意取得後から試験期終了まで、プロトンポンプインヒビター、抗生物質、整腸剤の使用を禁止する。ただし、抗生物質の局所での治療目的に限り使用可能とする。

なお、有害事象の治療等の理由によりやむを得ず薬剤を使用した場合、研究責任医師等はその薬剤名、使用期間、使用目的等を症例報告書に記載する。

1. 研究実施期間

2015年4月1日～2016年3月31日

1. 研究方法

本研究は以下の手順及びスケジュールにて行う。

- 1. 被験者募集

本学内の掲示板等に本研究の概要を掲示し、被験者を募集する。

- 1. 研究手順

研究開始最初の6例（各群3例）は、試験薬投与または非投与期間を28日間とし、その結果に基づき、7例目以降の試験薬投与または非投与期間を14日間とするか28日間とするかを決定する。

[同意取得]

1. 研究責任医師等は、説明文書を用いて本研究の内容を説明し、文書にて同意を取得する。
2. 同意取得後、研究責任医師等にて問診・身体測定等を行い、参加の適否を判断する。
3. 参加が可能と判断した場合は、被験者を登録する。また、ラクツロース/マンニトール試験、血中エンドトキシン活性測定及び腸内細菌叢検査を実施する。

［腸管透過性の亢進］

1. ジクロフェナクナトリウム（75mg/day）を1日3回毎食後7日間投与する。
2. ジクロフェナクナトリウムを7日間投与後、診察を行い、試験期に移行可能な被験者を試験薬投与群または対照群に割り付ける。
3. ラクツロース/マンニトール試験、血中エンドトキシン活性測定及び腸内細菌叢検査を実施する。

試験期について、研究開始最初の6例（各群3例）は、試験薬投与または非投与期間を28日間とする。7例目以降は、6例までの結果に基づき、試験薬投与または非投与期間を14日間又は28日間のいずれかに決定する。

【最初の6例まで】

[試験期]

（試験薬投与群の場合）

1. 試験薬1カプセルを1日1回昼食後に14日間投与する。被験者には「被験者が遵守すべき事項」に従い生活してもらう。
2. 試験薬を14日間投与後、診察を行い、ラクツロース/マンニトール試験、血中エンドトキシン活性測定及び腸内細菌叢検査を実施する。
3. 試験薬1カプセルを1日1回昼食後に14日間投与する。
4. 試験薬を14日間投与後、診察を行い、ラクツロース/マンニトール試験、血中エンドトキシン活性測定及び腸内細菌叢検査を実施する。

（対照群の場合）

1. 試験薬は投与しない（14日間）。被験者には「被験者が遵守すべき事項」に従い生活してもらう。
2. 14日後、診察を行い、ラクツロース/マンニトール試験、血中エンドトキシン活性測定及び腸内細菌叢検査を実施する。
3. 試験薬は投与しない（14日間）。
4. 14日後、診察を行い、ラクツロース/マンニトール試験、血中エンドトキシン活性測定及び腸内細菌叢検査を実施する。

【7例目以降】

[試験期]

（試験薬投与群の場合）

1. 試験薬1カプセルを1日1回昼食後に14日間又は28日間投与する。被験者には「被験者が遵守すべき事項」に従い生活してもらう。
2. 試験薬を14日間又は28日間投与後、診察を行い、ラクツロース/マンニトール試験、血中エンドトキシン活性測定及び腸内細菌叢検査を実施する。

（対照群の場合）

1. 試験薬は投与しない（14日間又は28日間）。被験者には「被験者が遵守すべき事項」に従い生活してもらう。
2. 14日又は28日後、診察を行い、ラクツロース/マンニトール試験、血中エンドトキシン活性測定及び腸内細菌叢検査を実施する。
   1. 研究スケジュール

- 1. 被験者が遵守すべき事項

研究責任医師等は試験期間中、被験者に以下について指導を行う。本研究は以下の手順及びスケジュールにて行う。

1. 研究責任医師等の指示に従い、ジクロフェナクナトリウム及び試験薬を服用する。
2. 試験薬は、昼食後の服用を忘れた場合（服用できなかった場合）は夕食後に服用する。夕食後の服用を忘れた場合（服用できなかった場合）、その日の試験薬は服用しない。
3. 試験期間中は、併用治療（薬剤（サプリメントを含む）及び療法）は禁止する。ただし、研究責任医師等が本試験中に発生した有害事象に対する治療が必要であると判断した場合はこの限りではない。
4. 試験期間中に異常を感じた場合、研究責任医師等に申し出る。
5. 研究期間中、大量に飲酒しない。
6. ラクツロース/マンニトール試験の実施前日21時以降及び当日は絶飲食とする。糖分を含まない少量の飲料は可とする。
7. 調査・検査項目及び時期
   1. 被験者背景

調査項目 ：生年月日、性別、身長、体重、合併症、既往歴、アレルギー歴、併用薬、排便回数、便の性状、下剤の使用の有無

調査対象　：試験薬投与群、対照群

調査時期 ：スクリーニング期

- 1. バイタルサイン

調査項目 ：血圧、脈拍数

調査対象　：試験薬投与群、対照群

調査時期 ：スクリーニング期、有害事象発現時、中止時

- 1. **理学的所見**
     調査項目 ：自他覚所見、有害事象、嘔気、排便回数、便の性状

調査対象　：試験薬投与群、対照群
調査時期 ：スクリーニング期、ジクロフェナク投与期、試験期、中止時

- 1. ジクロフェナクの服薬状況

調査項目 ：投与期間、1日投与量、休薬・中止等の有無、休薬・中止理由、変更理由

調査対象　：試験薬投与群、対照群

調査時期 ：ジクロフェナク投与期

- 1. 試験薬の服薬状況

調査項目 ：投与期間、1日投与量、投与量変更の有無、休薬・中止等の有無、変更理由

調査対象　：試験薬投与群

調査時期 ：試験期

- 1. ラクツロース／マンニトール試験

調査対象　：試験薬投与群、対照群

調査時期 ：スクリーニング期、ジクロフェナク投与期、試験期、中止時

- 1. 血中エンドトキシン活性

調査対象　：試験薬投与群、対照群

調査時期 ：スクリーニング期、ジクロフェナク投与期、試験期、中止時

採血量 ：1回2mL

- 1. 腸内細菌叢

調査対象　：試験薬投与群、対照群

調査時期 ：スクリーニング期、ジクロフェナク投与期、試験期、中止時

1. 評価項目
   1. 有効性評価項目

主要評価項目：ラクツロース／マンニトール試験を用いた小腸腸管透過性

副次評価項目：血中エンドトキシン活性、腸内細菌叢の変化

- 1. 安全性評価項目

有害事象、副作用

1. 有害事象
   1. 有害事象

有害事象とは、被験者に生じたあらゆる好ましくないあるいは意図しない徴候（臨床検査値の異常を含む）、症状又は病気のことであり、試験薬との因果関係は問わない。本研究の有害事象の収集期間は、ジクロフェナクナトリウム投与開始から試験期終了までとする。

有害事象は臨床症状に基づいて評価する。

研究責任医師等は、全ての有害事象に対して発現日、消失日、重篤性、重症度、処置の有無、転帰、試験薬との因果関係について判定し、症例報告書に記入する。

- 1. 重篤な有害事象

重篤な有害事象とは、有害事象のうち、次のものをいう。

1. 死亡
2. 死亡につながるおそれのあるもの
3. 治療のために病院又は診療所への入院、又は入院期間の延長が必要とされるもの（検査入院は除く）
4. 障害
5. 障害につながるおそれのあるもの
6. 1)～5)に準じて重篤であるもの
7. 後世代における先天性の疾病又は異常
   1. 有害事象の連絡及び対応

研究責任医師等は、有害事象が発現したことを知った場合、被験者の安全の確保を最優先に適切な医療行為を保険診療内で講ずる(例として内視鏡検査や投薬治療等)。また、研究責任医師等は、ルビプロストンによる有害事象が発現した場合（重篤性は問わない）は、直ちにアボットジャパン株式会社当院担当MRに連絡する。さらに、重篤な有害事象が発現した場合は、その旨を直ちに臨床研究機関の長にも通知する。

- 1. 試験薬との因果関係

有害事象と試験薬との因果関係の判定の分類は、次のとおりとする。

1. 関連あり

時間的に明白な相関関係があり、試験薬以外の要因が除外される場合。

1. 関連なし

時間的に相関関係がない場合、あるいは他の要因によると明確に説明できる場合。

- 1. 有害事象の程度（重症度）

有害事象の程度の判定基準は、次のとおりとする。

1. 軽度

被験者の日常生活に影響がない程度であり、事象に対する処置を必要としない程度。

1. 中等度
   被験者の日常生活にその事象が原因で多少の支障がある程度であり、事象に対する処置あるいは研究の中止（被験者の希望による中止を除く）を必要とする程度。
2. 高度

被験者の日常生活がその事象が原因でできない程度であり、研究を中止し（被験者の希望による中止を除く）、さらに何らかの処置をせざるを得ない程度。

- 1. 副作用の定義

有害事象のうち、試験薬との因果関係が否定できないもの（「1)明らかに関連あり」、「2)多分関連あり」、「3)関連なしとはいえない」）を副作用として取り扱う。
予測できない副作用とは、副作用のうち、試験薬の医薬品インタビューフォーム(2014年)に記載されていないもの、あるいは記載されていてもその性質や重症度が記載内容とー致しないものをいう。

- 1. 試験薬の予想される副作用

ボルタレンの予想される副作用は、次のとおりである（試験薬の医薬品インタビューフォーム（2015年）に基づく）。

承認時までの調査例数1,474例中、160例（10.85%）に239件の副作用が認められた。症状としては胃部不快感等の消化器症状139例（9.43%）が主なものであり、他に、浮腫14例（0.95%）、発疹等の皮膚症状23例（1.56%）等がみられている。

なお、市販後の使用成績調査では、35,653例中2,749例（7.71%）に4,545件の副作用が認められた。症状としては、消化器症状2,365例（6.63%）、ついで浮腫などの全身症状215例（0.60%）、皮膚症状172例（0.48%）などがみられている。

その他の副作用としての消化性潰瘍と肝障害に関しての報告は0.1%未満である。

アミティーザの予想される副作用は、次のとおりである（試験薬の医薬品インタビューフォーム（2014年）に基づく）。

国内で実施された臨床試験3試験において、試験薬1日48µg（24µgの1日2回投与）が投与された315例中196例（62％）に臨床検査値異常変動を含む副作用が認められた。主な副作用は、下痢95例（30％）、悪心73例（23％）等であった。

1. 被験者の中止基準

次のいずれかに該当する被験者は、本研究を中止する。

1. 被験者自身が研究参加の撤回を申し出た者
2. 規定されたジクロフェナクナトリウム及び試験薬の服用ができなかった者
3. 規定された検査ができなかった者
4. 試験期への移行ができないと研究責任医師等が判断した者
5. 研究開始後に選択基準に合致していないこと又は除外基準に抵触することが判明した者
6. CTCAC v4.0のGrade 2以上(11.6項で定めた中等症以上)に該当する副作用が生じた者
7. その他、研究責任医師等が研究を中止するべきと判断した者

本研究を中止した場合は中止後の検査は被験者からの要望がない限り、原則として行わない。

1. 健康被害補償

被験者に健康被害が生じた場合、臨床研究機関はその治療に関する医療の提供など必要かつ適切な処置を行う。その際、当該健康被害が試験薬の適正使用により生じ、試験薬との因果関係があると研究責任医師が判断した場合、研究責任医師はその補償費用を負担する。ただし、医療費、医療手当は支払われない。なお、健康被害が被験者の故意又は重大な過失によるものと判断した場合には、補償の対象外となる場合がある。

本研究に起因する健康被害による賠償責任が生じた場合の履行措置として、研究責任医師は、研究責任医師等及び臨床研究機関が被保険者となる保険に加入する。

1. 研究の倫理的及び科学的実施
   1. ヘルシンキ宣言等の遵守

本研究はヘルシンキ宣言、臨床研究に関する倫理指針及び本研究計画書を遵守して実施する。

- 1. 倫理委員会

1. 研究責任医師は、研究の実施に先立ち、臨床研究機関における倫理委員会にて審査を受け、承認を得た後に実施する。
2. 研究責任医師は、研究計画書の変更又は安全性に関わる新たなる情報が得られた場合、倫理委員会に本研究継続の可否の検討を依頼する。
3. 研究責任医師は、倫理委員会の継続審査を受けるために、年に1回又は臨床研究機関の手順に従い、臨床研究機関の長に研究の実施状況を文書で報告する。
4. 研究計画書の承認・遵守・変更
   1. 研究計画書の承認

研究責任医師は、研究開始に先立ち、研究計画書の内容について臨床研究機関の長の承認を得る。

- 1. 研究計画書の遵守

研究責任医師等は、倫理委員会の事前の審査に基づく文書による承認を得ることなく、研究計画書からの逸脱又は変更を行わない。ただし、被験者の緊急の危険を回避するためのものである等医療上やむを得ないものである場合は、この限りではない。臨床研究機関の手順に基づき、倫理委員会及び臨床研究機関の長に報告する。

- 1. 研究計画書の変更

研究責任医師は、倫理委員会の事前の審査に基づく文書による承認を得ることなく、研究計画書の変更を行わない。

1. 研究の終了又は中止及び中断
   1. 研究の終了

本研究が終了した場合は、研究責任医師は、臨床研究機関の長に本研究が終了した旨を報告する。

- 1. 研究の中止及び中断

研究責任医師は、以下のいずれかの項目に該当する場合、本研究を中止又は中断する。

1. 被験者の安全性確保など、倫理上あるいは医療上やむを得ない事情が発生した場合
2. 本研究を実施する科学的妥当性が失われた場合
3. 本研究の方針に変更があった場合
4. 研究責任医師等又は臨床研究機関による重大又は継続した不遵守が認められた場合
5. 倫理委員会が実施中の研究の継続審査等において、研究の中止又は中断の決定を下した場合
6. 研究責任医師の異動等により、研究の継続が不可能な場合
7. 選択基準に適合する被験者が見込めなくなった場合
8. 研究責任医師が研究を中止・中断を判断した場合
9. 症例報告書の作成
   同意を取得した被験者において症例報告書を作成する。
10. 研究の品質管理

本研究の品質管理として、研究責任医師は、モニターを指名する。モニターは、本研究が研究計画書及び臨床研究に関する倫理指針に沿って実施されている事を確認する。

1. 研究の品質保証

本研究の品質保証として、研究責任医師は、監査担当者を指名する。監査担当者は、本研究が研究計画書及び臨床研究に関する倫理指針に沿って実施されている事を確認する。

1. 被験者の人権保護
   1. 個人情報管理の担当者
      研究責任医師は、臨床研究機関の規定に準じて、個人情報管理の担当者を指名する。
   2. 個人情報の取り扱い
      研究責任医師等は被験者登録及び症例報告書の作成にあたり、被験者を被験者識別コードで特定し、連結可能匿名化の上、保存し、被験者の個人情報の取扱いは慎重に行う。
2. 記録の保存及び破棄
   1. 記録の保存
      研究責任医師等は、症例報告書、検査データ、倫理委員会の記録、被験者の同意に関する記録、投薬記録等、本研究に関連するデータを、研究終了後5年間保管する。
   2. 記録の破棄
      本研究に関する記録は、保管期限終了後、被験者の個人情報等の秘密を侵害しないよう適切に処分するものとする。
3. 統計解析

本研究における主な統計的事項は、以下のとおりである。詳細は、統計解析計画書に別途定めることとする。

- 1. 人口統計学的特性
     被験者の概要を示すとともに、人口統計学的変数（性別、年齢など）について要約統計量を示す。
  2. 解析対象集団
     本研究における解析対象集団は、本研究に登録された被験者のうち次に該当する被験者を除いた最大の解析対象集団（FAS：Full Analysis Set）とする。

・登録後に不適格であることが判明した被験者

・試験薬が1回も投与されなかった被験者

・有効性に関する評価が全くない被験者

- 1. 有効性評価項目の解析方法
     1. 主要評価項目

小腸腸管透過性については、ベースラインと治療後のそれぞれについて、要約統計量を算出する（平均値、中央値）。

主たる解析については、各被験者に対して（治療後の小腸腸管透過性－ベースラインの小腸腸管透過性）を求め、対応のあるWilcoxon検定を実施する。有意水準は両側5%とする。参考までに、ベースライン小腸腸管透過性を共変量とした共分散分析（Analysis of Covariance）により、2群間を比較する。

- - 1. 副次評価項目

血中エンドトキシン活性、腸内細菌叢の変化についても、各被験者に対して（治療後の値－ベースラインの値）を求め、対応のあるWilcoxon検定を実施する。有意水準は両側5%とする。参考までに、ベースライン値を共変量とした共分散分析（Analysis of Covariance）により、2群間を比較する。

- 1. 安全性評価項目の解析方法

発現した有害事象及び副作用の種類、程度、発現率を算出し、2群間で比較する。

22.5 症例数の設計

本試験は、パイロット的な位置づけとして、健常人を対象に行われる。先行データが存在しないため、定められた研究期間内で集積可能と考えられる30例を予定登録数とする。

1. 本研究に係る資金源及び利益相反

本研究は、横浜市立大学によるアボットジャパン株式会社からの委託研究として実施し、本研究の実施に必要な資金はアボットジャパン株式会社が負担する。

研究責任者は、臨床研究機関の利益相反の管理に関する規定に従い、利益相反について申告する。

なお、本研究で使用するデータベースは研究責任医師が匿名化の上で作成し、アボットジャパン株式会社はアクセスできないものとし、また、統計解析業務も同様とする。本研究の結果の評価及び解釈は、研究責任医師が行うものとする。開示に関しては、秘密保持規約に従って文書での同意が得られない限りはされない。

1. 研究参加に係る費用

研究協力費の支払いについては、1症例あたり5万円とする。

本研究で使用するジクロフェナクナトリウム及び試験薬は、研究責任医師が購入する。

1. 結果の公表

研究責任医師は、本研究の結果の如何にかかわらず、文献あるいは学会等にて結果を公表する。

なお、研究責任医師または臨床研究機関は、結果公表内容を提出する60日前までに、同内容の原稿をアボットジャパン株式会社に提出し、アボットジャパン株式会社のレビューを受け、意見を求めるものとする。

1. 研究組織
   1. 研究責任者（研究責任医師）

横浜市立大学附属病院　肝胆膵消化器病学 主任教授 中島淳

- 1. 研究分担者

横浜市立大学附属病院　肝胆膵消化器病学 非常勤 加藤孝征

横浜市立大学附属病院　肝胆膵消化器病学 指導診療医 結束貴臣

横浜市立大学附属病院　肝胆膵消化器病学 非常勤 本多靖

　　　　　　横浜市立大学大学院医学研究科　臨床統計学　　　　　　 教授　　　　　　　　　山中竹春

- 1. 個人情報管理者
     横浜市立大学附属病院　肝胆膵消化器病学 准教授 斉藤　聡
  2. 統計解析責任者

横浜市立大学大学院医学研究科　臨床統計学　 教授　　　　　　　　　山中竹春

- 1. モニタリング担当者

横浜市立大学附属病院　肝胆膵消化器病学　　　　　　　　准教授　　　　　　　斉藤　聡

- 1. 監査担当者

横浜市立大学附属病院　肝胆膵消化器病学　　　　　　　　指導診療医　　　　　内山詩織

1. 文献

[1] Nonsteroidal anti-inflammatory drug-induced visible and invisible small intestinal injury.

J Clin Biochem Nutr. 2013 Jul;53(1):55-9. doi: 10.3164/jcbn.12-116. Epub 2013 Apr 9.

[2] Lubiprostone Increases Spontaneous Bowel Movement Frequency and Quality of Life in Patients With Chronic Idiopathic Constipation.

[Clin Gastroenterol Hepatol.](http://www.ncbi.nlm.nih.gov/pubmed/?term=Lubiprostone+Increases+Spontaneous+Bowel+Movement+Frequency+and+Quality+of+Life+in+Patients+With+Chronic+Idiopathic+Constipation) 2014 Aug 24. pii: S1542-3565(14)01245-2

[3] Clinical trial: lubiprostone in patients with constipation associated irritable bowel syndrome

　 – results of two randomized, placebo-controlled studies

Aliment Pharmacol Ther. 2009 Feb 1;29(3):329-41.
