# Supplementary material for: Lubiprostone improves intestinal permeability in humans, a novel therapy for the leaky gut: A prospective randomized pilot study in healthy volunteers
Source: PLoS One. 2017 Apr 14;12(4):e0175626. doi: 10.1371/journal.pone.0175626 (PMC5391961; doi:10.1371/journal.pone.0175626)
Supplement: S1 File — (PDF) [file pone.0175626.s003.pdf]

Replace with Main Title

Your Name

2017-02-28

```
> #####Calculate sample size for comparison between two means#####
> quartz(width=7, height=7); par(lwd=1, las=1, family="sans", cex=1, mgp=
> SampleMean(0.01205, 0.00323, 0.05, 0.80, 2, 1)
```

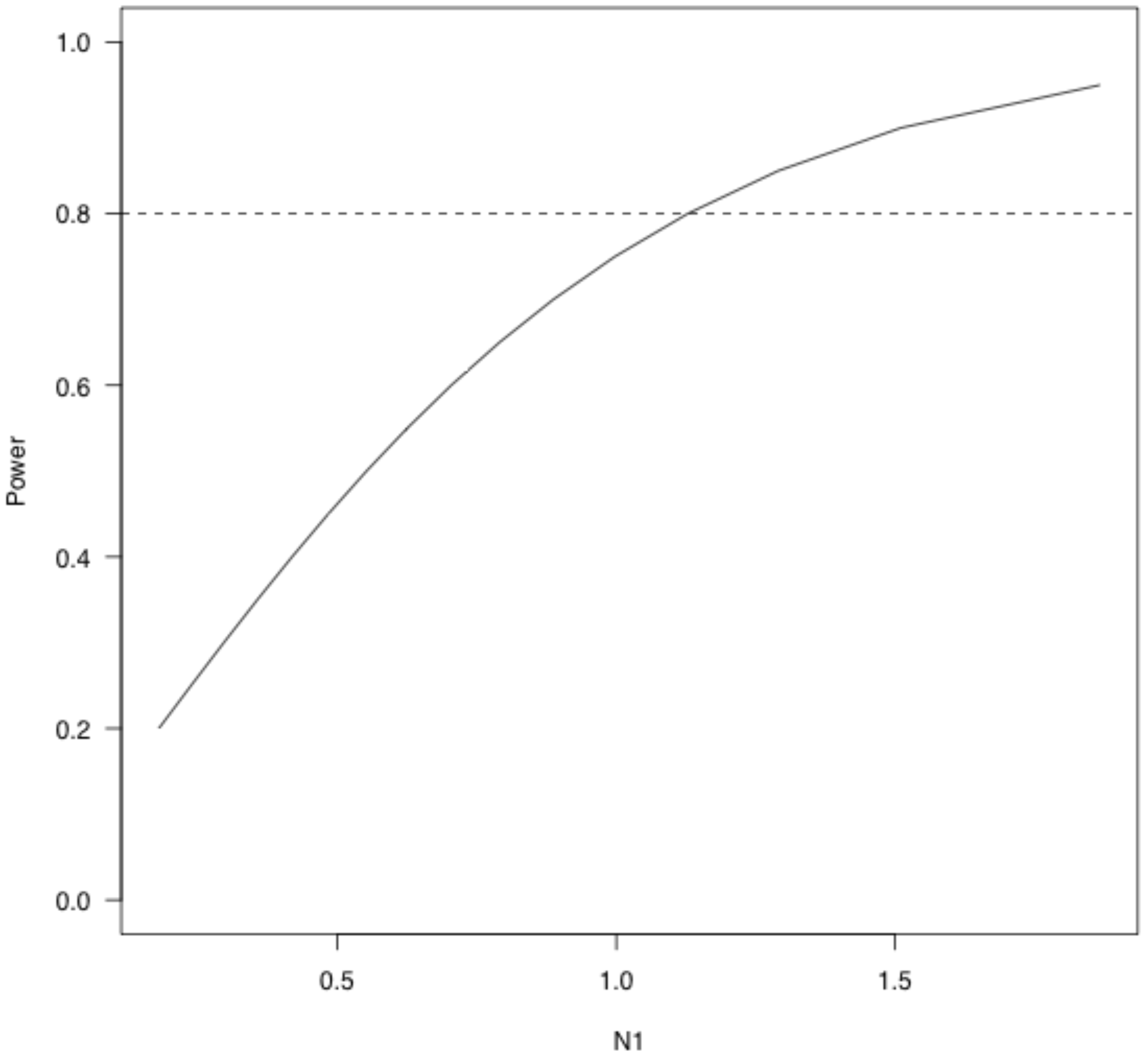

```
                     Assumptions
Difference in means      0.01205
Standard deviation       0.00323
Alpha                       0.05
                       two-sided
Power                        0.8
N2/N1                          1

Required sample size   Estimated
N1                             2
N2                             2
```

```
> #####Calculate sample size for comparison between two means#####
> quartz(width=7, height=7); par(lwd=1, las=1, family="sans", cex=1, mgp=
> SampleMean(0.01205, 0.01074, 0.05, 0.90, 2, 1)
```

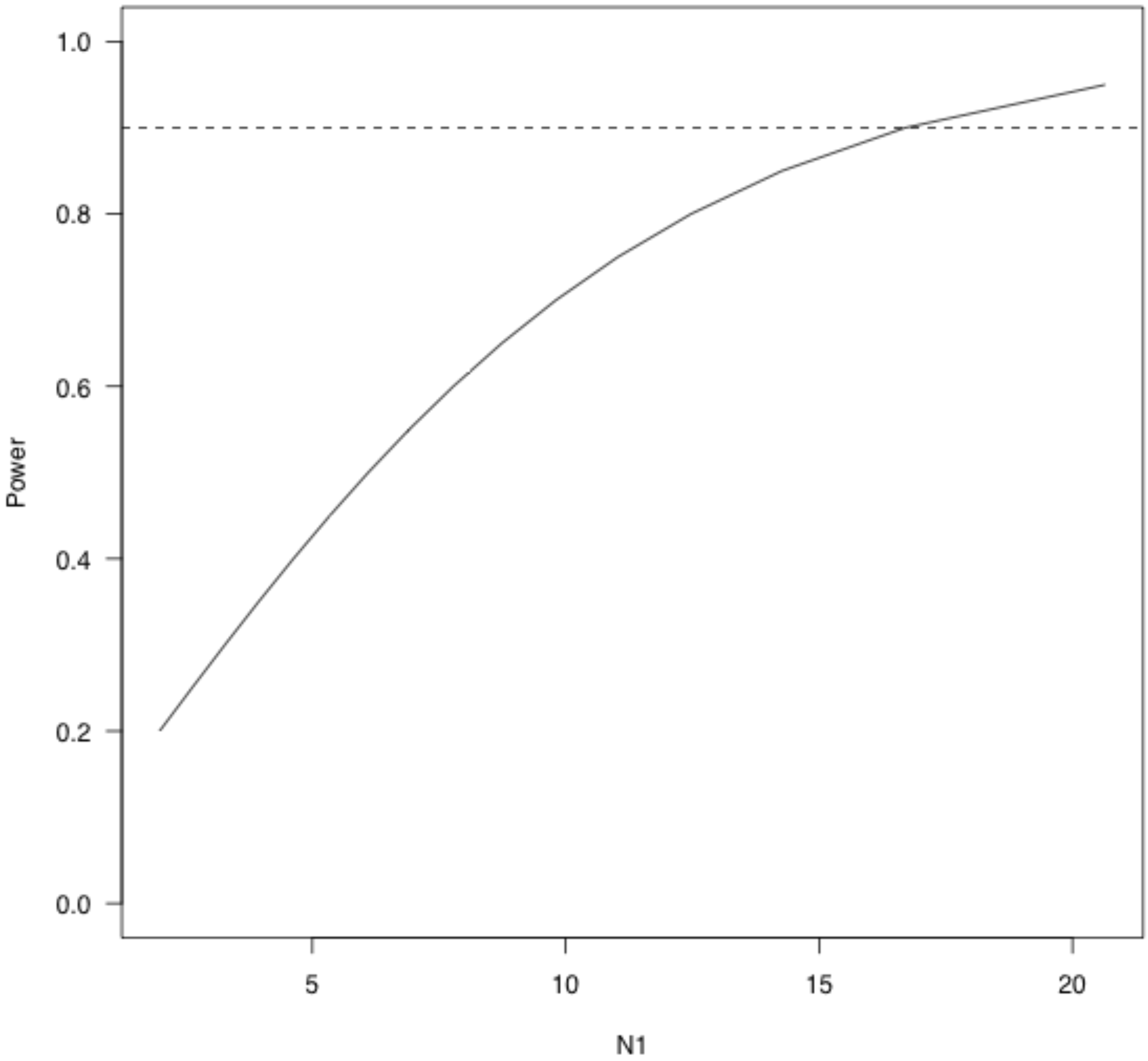

	Assumptions
Difference in means	0.01205
Standard deviation	0.01074
Alpha	0.05
	two-sided
Power	0.9
N2/N1	1
Required sample size	Estimated
N1	17
N2	17

```
> #####Calculate sample size for comparison between two means#####
> quartz(width=7, height=7); par(lwd=1, las=1, family="sans", cex=1, mgp=
> SampleMean(0.00298, 0.01652, 0.05, 0.90, 2, 1)
```

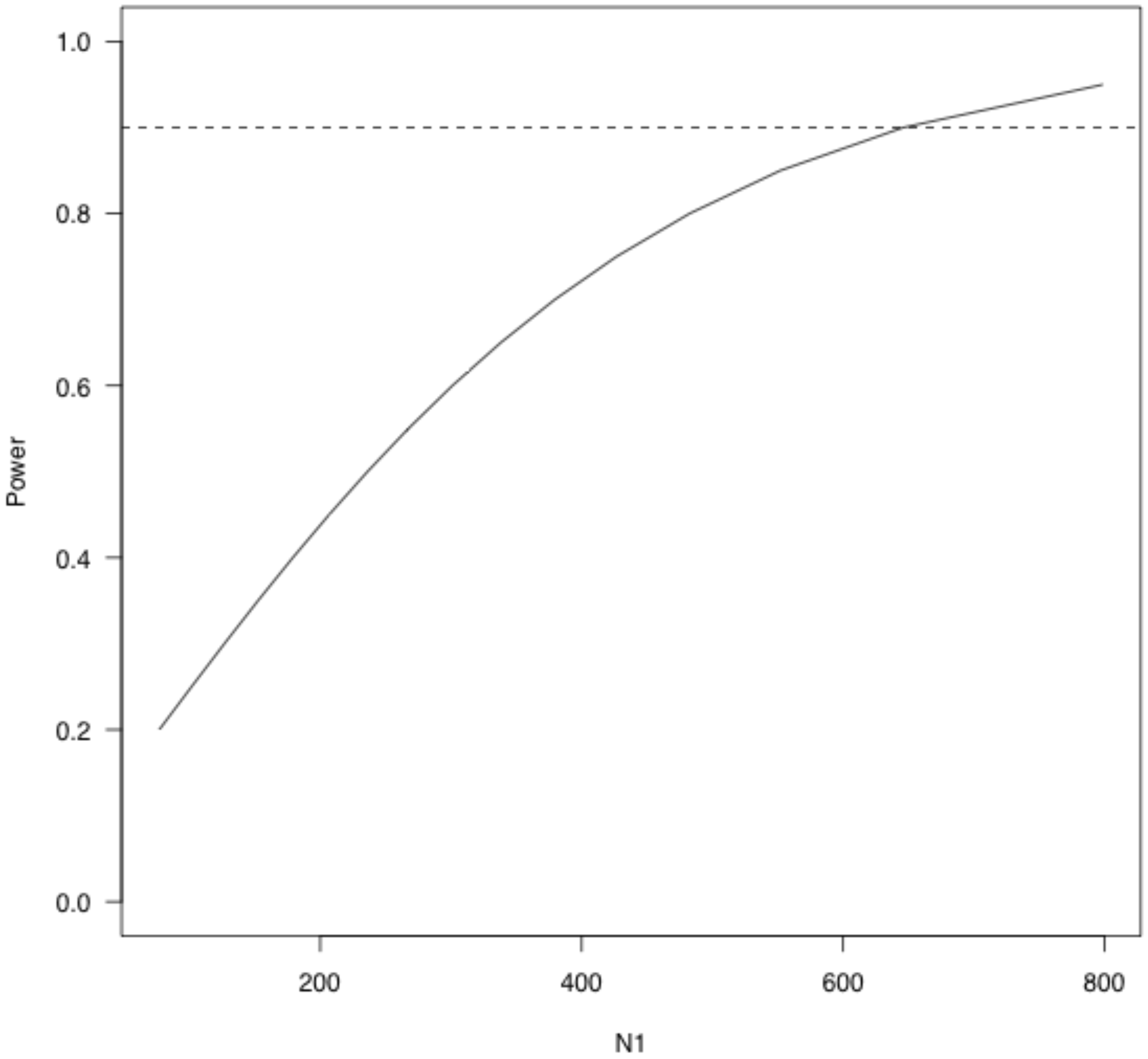

```
                     Assumptions
Difference in means      0.00298
Standard deviation       0.01652
Alpha                       0.05
                       two-sided
Power                        0.9
N2/N1                          1

Required sample size   Estimated
N1                           646
N2                           646
```

```
> #####Calculate sample size for comparison between two means#####
> quartz(width=7, height=7); par(lwd=1, las=1, family="sans", cex=1, mgp=
> SampleMean(0.00298, 0.01240, 0.05, 0.80, 2, 1)
```

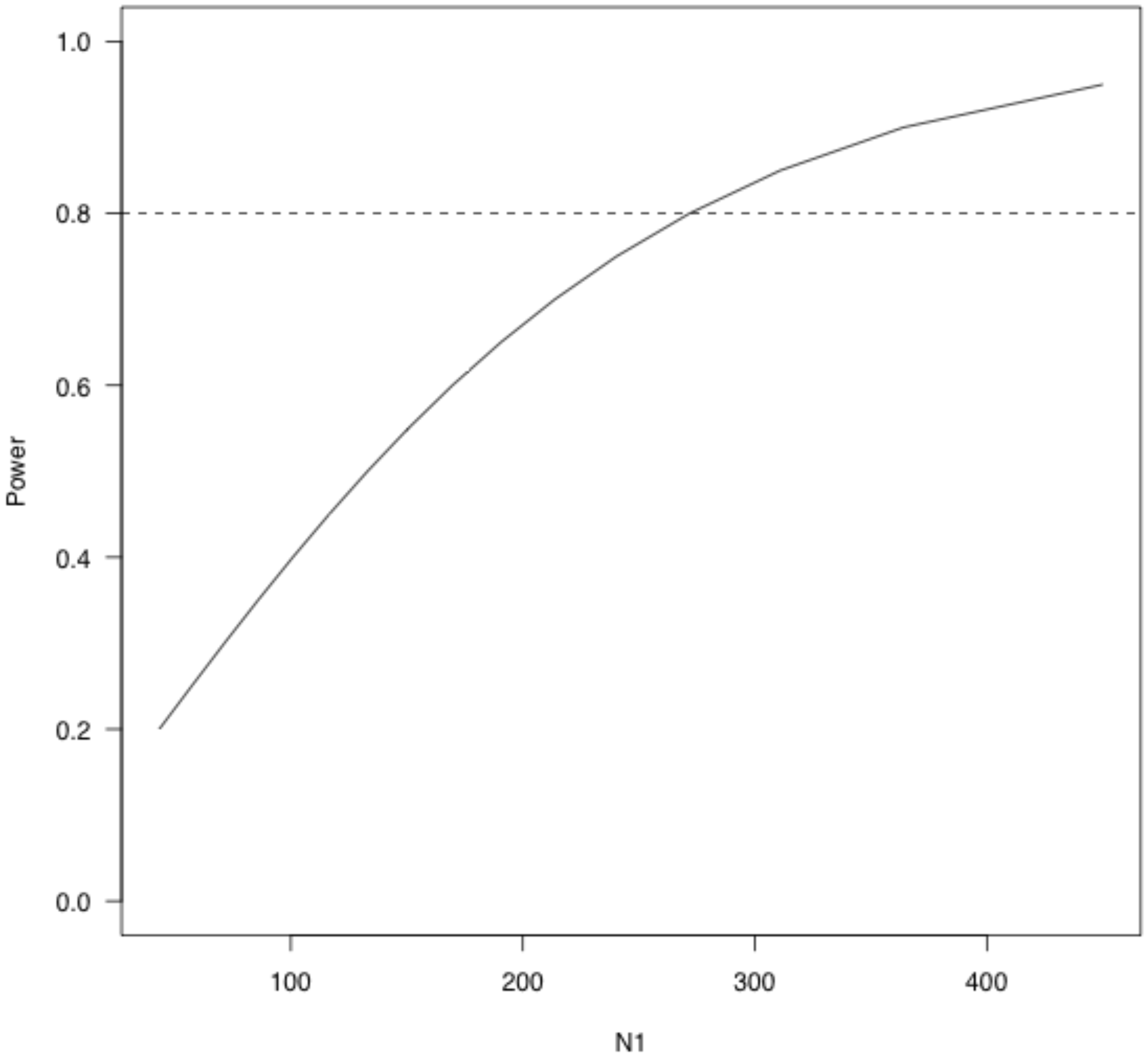

	Assumptions
Difference in means	0.00298
Standard deviation	0.0124
Alpha	0.05
	two-sided
Power	0.8
N2/N1	1
Required sample size	Estimated
N1	272
N2	272
