# Supplementary material for: Lubiprostone improves intestinal permeability in humans, a novel therapy for the leaky gut: A prospective randomized pilot study in healthy volunteers
Source: PLoS One. 2017 Apr 14;12(4):e0175626. doi: 10.1371/journal.pone.0175626 (PMC5391961; doi:10.1371/journal.pone.0175626)
Supplement: S2 File — (PDF) [file pone.0175626.s004.pdf]

Replace with Main Title

Your Name

2017-02-23

```
> #####Read Data From Text File#####
> primary_data <- read.table("/Users/takakato222/Desktop/primary data.csv
+   header=TRUE, sep=",", na.strings=c("", "NA"), dec=".", fill=TRUE,
+   quote="\"", strip.white=TRUE)
> primary_data <- read.table("/Users/takakato222/Desktop/primary data.csv
+   na.strings=c("", "NA"), dec=".", fill=TRUE, quote="\"", strip.white=T
```

```
> #####Two-sample t-test#####
> group.names <- NULL
> group.means <- NULL
> group.sds <- NULL
> group.p <- NULL
> res <- NULL
> (res <- t.test(age~factor(Group), alternative='two.sided', conf.level=0
+   data=primary_data))
```

```


    Welch Two Sample t-test

data:  age by factor(Group)
t = -0.85883, df = 24.518, p-value = 0.3988
alternative hypothesis: true difference in means is not equal to 0
95 percent confidence interval:
 -2.914687  1.200401
sample estimates:
mean in group A mean in group B 
       22.92857        23.78571 
```

```
> quartz(width=7, height=7); par(lwd=1, las=1, family="sans", cex=1, mgp=
> boxplot(age~ factor(Group), ylab="age", xlab="Group", data=primary_data
```

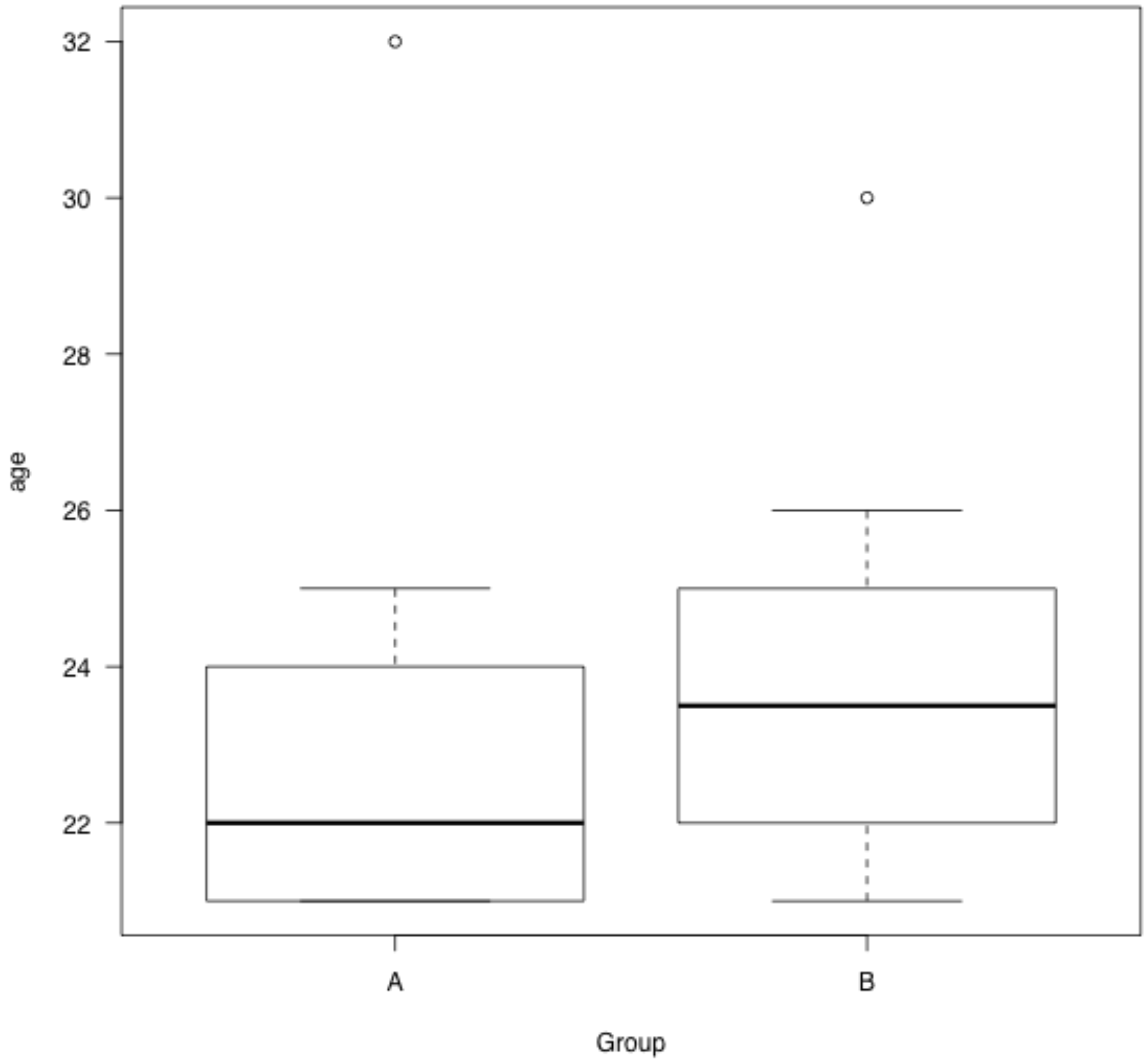

32
30
28
26
24
22
age
A
B
Group

```
> bar.means <- tapply(primary_data$age, factor(primary_data$Group), mean,
> bar.sds <- tapply(primary_data$age, factor(primary_data$Group), sd, na.
> group.names <- c(group.names, "Group=A")
> group.means <- c(group.means, bar.means[1])
> group.sds <- c(group.sds, bar.sds[1])
> group.p <- c(group.p, signif(res$p.value,digits=3))
> group.names <- c(group.names, "Group=B")
> group.means <- c(group.means, bar.means[2])
> group.sds <- c(group.sds, bar.sds[2])
> group.p <- c(group.p, "")
> summary.ttest <- NULL
> summary.ttest <- data.frame(mean=group.means, sd=group.sds, p.value=gro
> rownames(summary.ttest) <- group.names
> colnames(summary.ttest) <- gettext(domain="R-RcmdrPlugin.EZR",colnames(
> summary.ttest
```

```
            mean       sd p.value
Group=A 22.92857 2.947340   0.399
Group=B 23.78571 2.293086
```

```
> #####Two-sample t-test#####
> group.names <- NULL
> group.means <- NULL
> group.sds <- NULL
> group.p <- NULL
> res <- NULL
> (res <- t.test(BMI~factor(Group), alternative='two.sided', conf.level=0
+   data=primary_data))
```

```


    Welch Two Sample t-test

data:  BMI by factor(Group)
t = -0.082608, df = 21.961, p-value = 0.9349
alternative hypothesis: true difference in means is not equal to 0
95 percent confidence interval:
 -1.864819  1.721962
sample estimates:
mean in group A mean in group B 
       22.64286        22.71429 
```

```
> quartz(width=7, height=7); par(lwd=1, las=1, family="sans", cex=1, mgp=
> boxplot(BMI~ factor(Group), ylab="BMI", xlab="Group", data=primary_data
```

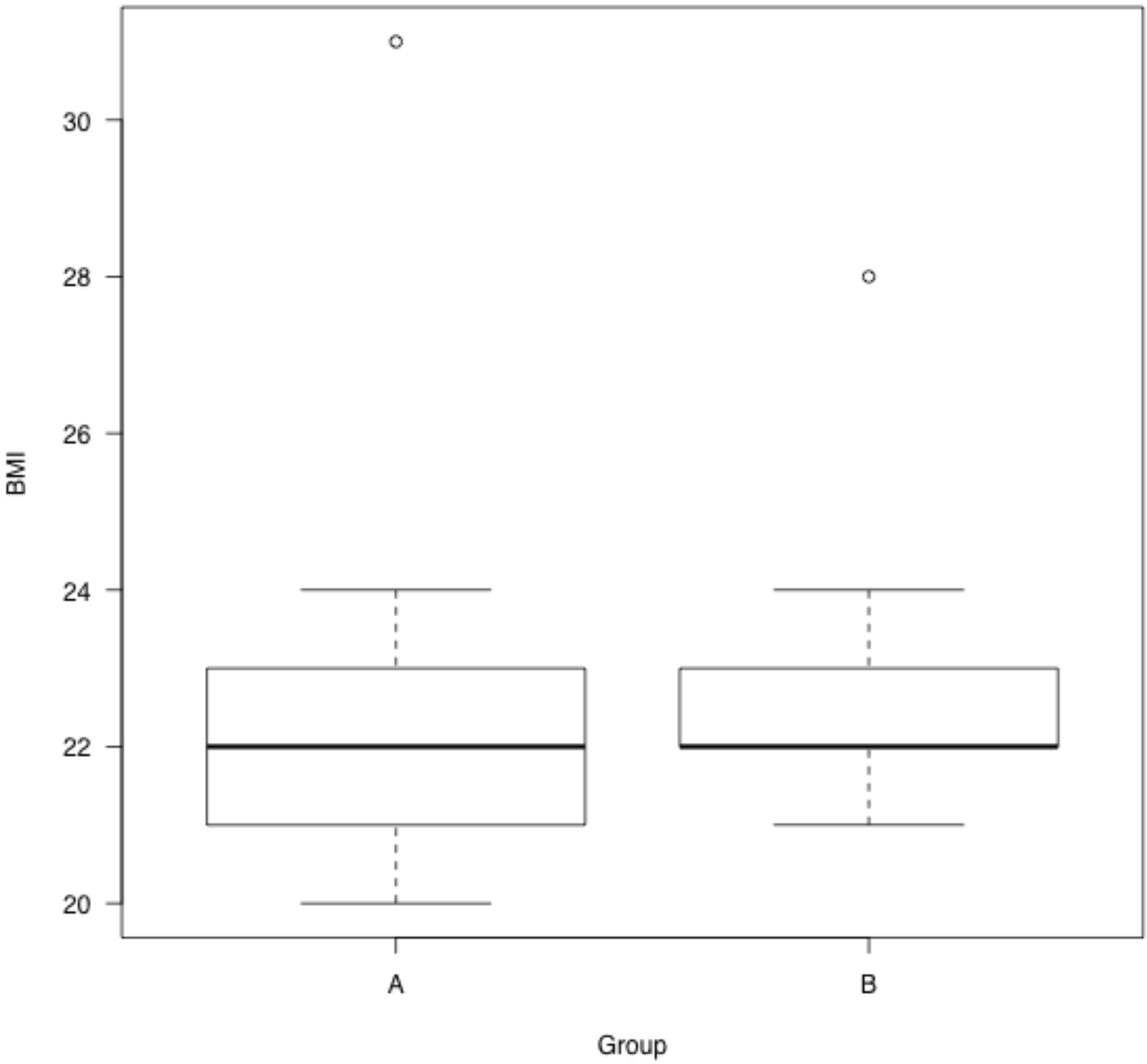
30
28
26
24
22
20
BMI
A
B
Group

```
> bar.means <- tapply(primary_data$BMI, factor(primary_data$Group), mean,
> bar.sds <- tapply(primary_data$BMI, factor(primary_data$Group), sd, na.
> group.names <- c(group.names, "Group=A")
> group.means <- c(group.means, bar.means[1])
> group.sds <- c(group.sds, bar.sds[1])
> group.p <- c(group.p, signif(res$p.value,digits=3))
> group.names <- c(group.names, "Group=B")
> group.means <- c(group.means, bar.means[2])
> group.sds <- c(group.sds, bar.sds[2])
> group.p <- c(group.p, "")
> summary.ttest <- NULL
> summary.ttest <- data.frame(mean=group.means, sd=group.sds, p.value=gro
> rownames(summary.ttest) <- group.names
> colnames(summary.ttest) <- gettext(domain="R-RcmdrPlugin.EZR",colnames(
> summary.ttest
```

```
            mean       sd p.value
Group=A 22.64286 2.734597   0.935
Group=B 22.71429 1.728876
```

```
> #####Two-sample t-test#####
> group.names <- NULL
> group.means <- NULL
> group.sds <- NULL
> group.p <- NULL
> res <- NULL
> (res <- t.test(LMR1~factor(Group), alternative='two.sided', conf.level=
+    data=primary_data))
```

```


    Welch Two Sample t-test

data:  LMR1 by factor(Group)
t = 0.39457, df = 22.995, p-value = 0.6968
alternative hypothesis: true difference in means is not equal to 0
95 percent confidence interval:
 -0.009225657  0.013574431
sample estimates:
mean in group A mean in group B 
     0.02079417      0.01861978 
```

```
> quartz(width=7, height=7); par(lwd=1, las=1, family="sans", cex=1, mgp=
> boxplot(LMR1~ factor(Group), ylab="LMR1", xlab="Group", data=primary_da
```

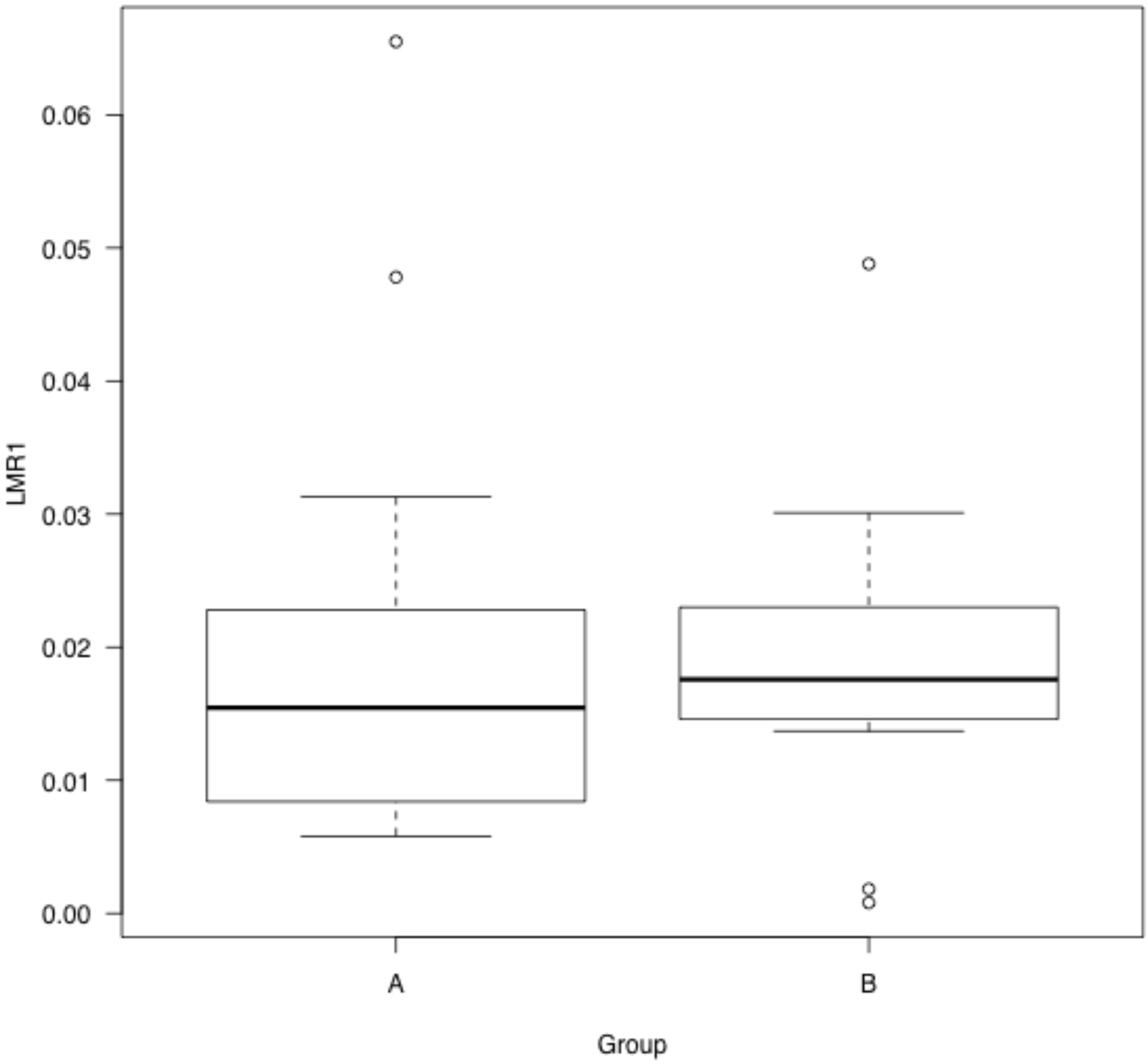
0.06
0.05
0.04
0.03
0.02
0.01
0.00
LMR1
A
B
Group

```
> bar.means <- tapply(primary_data$LMR1, factor(primary_data$Group), mean
> bar.sds <- tapply(primary_data$LMR1, factor(primary_data$Group), sd, na
> group.names <- c(group.names, "Group=A")
> group.means <- c(group.means, bar.means[1])
> group.sds <- c(group.sds, bar.sds[1])
> group.p <- c(group.p, signif(res$p.value,digits=3))
> group.names <- c(group.names, "Group=B")
> group.means <- c(group.means, bar.means[2])
> group.sds <- c(group.sds, bar.sds[2])
> group.p <- c(group.p, "")
> summary.ttest <- NULL
> summary.ttest <- data.frame(mean=group.means, sd=group.sds, p.value=gro
> rownames(summary.ttest) <- group.names
> colnames(summary.ttest) <- gettext(domain="R-RcmdrPlugin.EZR",colnames(
> summary.ttest
```

```
              mean         sd p.value
Group=A 0.02079417 0.01701260   0.697
Group=B 0.01861978 0.01165042
```

```
> #####ANCOVA#####
> TempDF <-  primary_data
> quartz(width=7, height=7); par(lwd=1, las=1, family="sans", cex=1, mgp=
> scatterplot(LMR2 ~ LMR1 | factor(Group), reg.line=lm, smooth=FALSE, spr
+   by.groups=TRUE, data=TempDF)
```

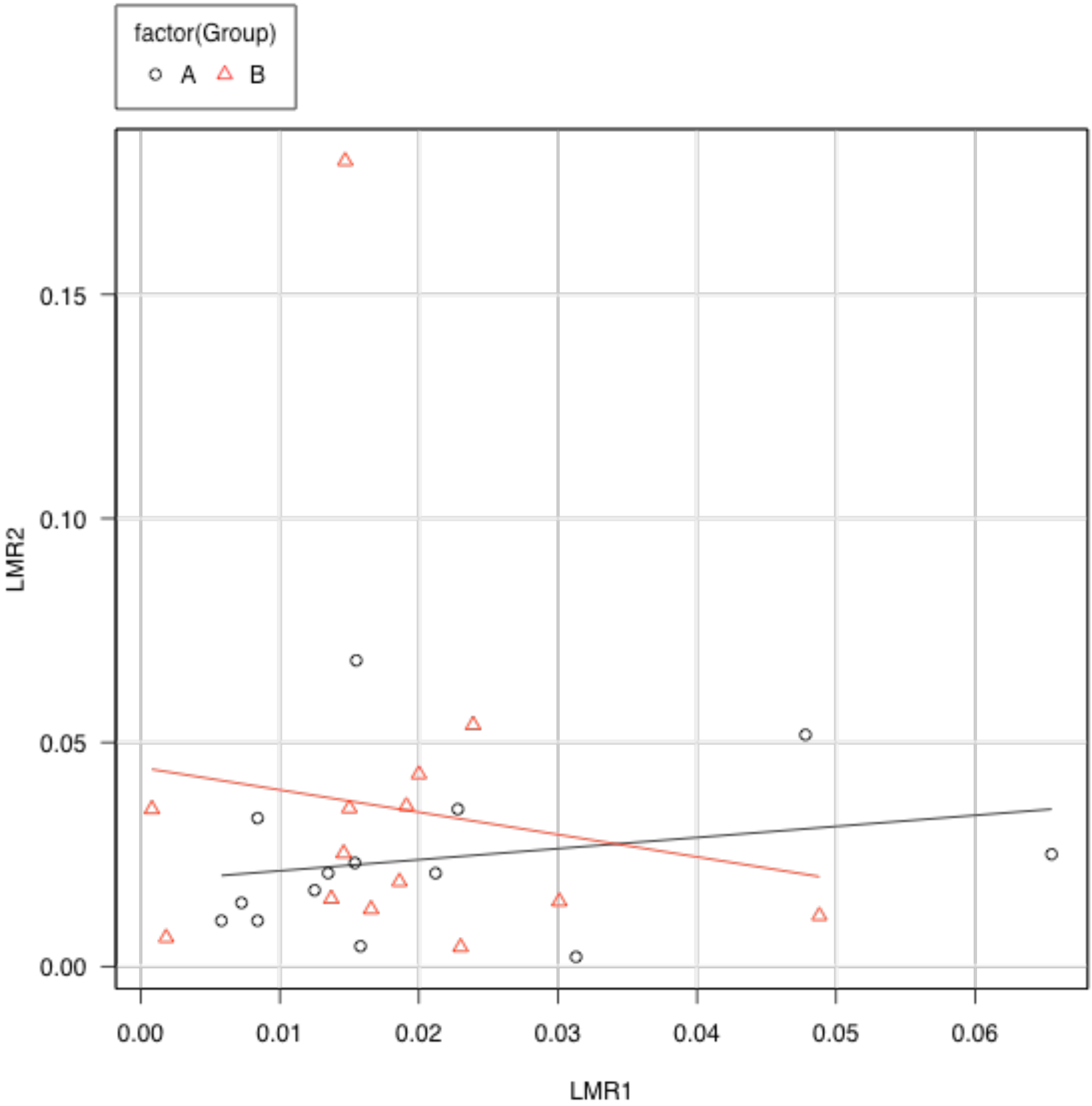


```
> cat(gettext(domain="R-RcmdrPlugin.EZR",
+   "P value for interaction between grouping variable and covariate is")
+ ")
```

```
P value for interaction between grouping variable and covariate is 0.464
```

```
> AnovaModel.1 <- AnovaModel.1 <- lm(LMR2 ~ 1 + factor(Group) + LMR1, dat
+   na.action=na.omit)
> Anova(AnovaModel.1, type="III")
```

```
Anova Table (Type III tests)

Response: LMR2
                 Sum Sq Df F value  Pr(>F)
(Intercept)   0.0037894  1  3.1877 0.08633 .
factor(Group) 0.0008577  1  0.7215 0.40372
LMR1          0.0000005  1  0.0004 0.98402
Residuals     0.0297189 25
---
Signif. codes:  0 '***' 0.001 '**' 0.01 '*' 0.05 '.' 0.1 ' ' 1
```

```
> #####ANCOVA#####
> TempDF <-  primary_data
> quartz(width=7, height=7); par(lwd=1, las=1, family="sans", cex=1, mgp=
> scatterplot(LMR3 ~ LMR1 | factor(Group), reg.line=lm, smooth=FALSE, spr
+   by.groups=TRUE, data=TempDF)
```

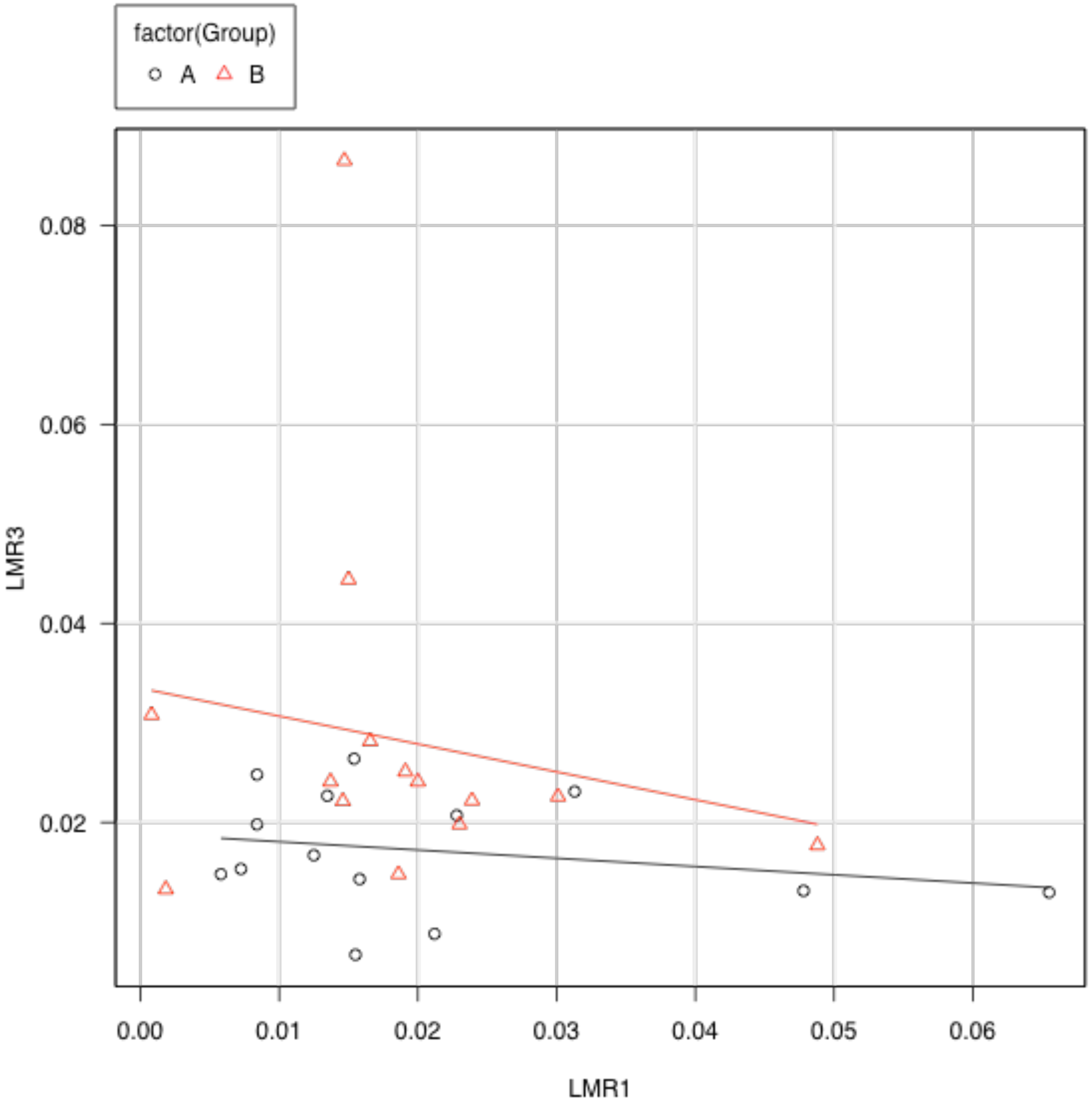


```
> cat(gettext(domain="R-RcmdrPlugin.EZR",
+   "P value for interaction between grouping variable and covariate is")
+ ")
```

```
P value for interaction between grouping variable and covariate is 0.63
```

```
> AnovaModel.2 <- AnovaModel.2 <- lm(LMR3 ~ 1 + factor(Group) + LMR1, dat
+   na.action=na.omit)
> Anova(AnovaModel.2, type="III")
```

```
Anova Table (Type III tests)

Response: LMR3
                 Sum Sq Df F value    Pr(>F)
(Intercept)   0.0027262  1 14.3803 0.0008433 ***
factor(Group) 0.0008098  1  4.2714 0.0492630 *
LMR1          0.0001184  1  0.6243 0.4368736
Residuals     0.0047395 25
---
Signif. codes:  0 '***' 0.001 '**' 0.01 '*' 0.05 '.' 0.1 ' ' 1
```

```
> #####Two-sample t-test#####
> group.names <- NULL
> group.means <- NULL
> group.sds <- NULL
> group.p <- NULL
> res <- NULL
> (res <- t.test(Lac.1~factor(Group), alternative='two.sided', conf.level
+   var.equal=FALSE, data=primary_data))
```

```


    Welch Two Sample t-test

data:  Lac.1 by factor(Group)
t = 0.069737, df = 24.149, p-value = 0.945
alternative hypothesis: true difference in means is not equal to 0
95 percent confidence interval:
 -0.04908311  0.05251720
sample estimates:
mean in group A mean in group B
     0.07040250      0.06868545
```

```
> quartz(width=7, height=7); par(lwd=1, las=1, family="sans", cex=1, mgp=
> boxplot(Lac.1~ factor(Group), ylab="Lac.1", xlab="Group", data=primary_
```

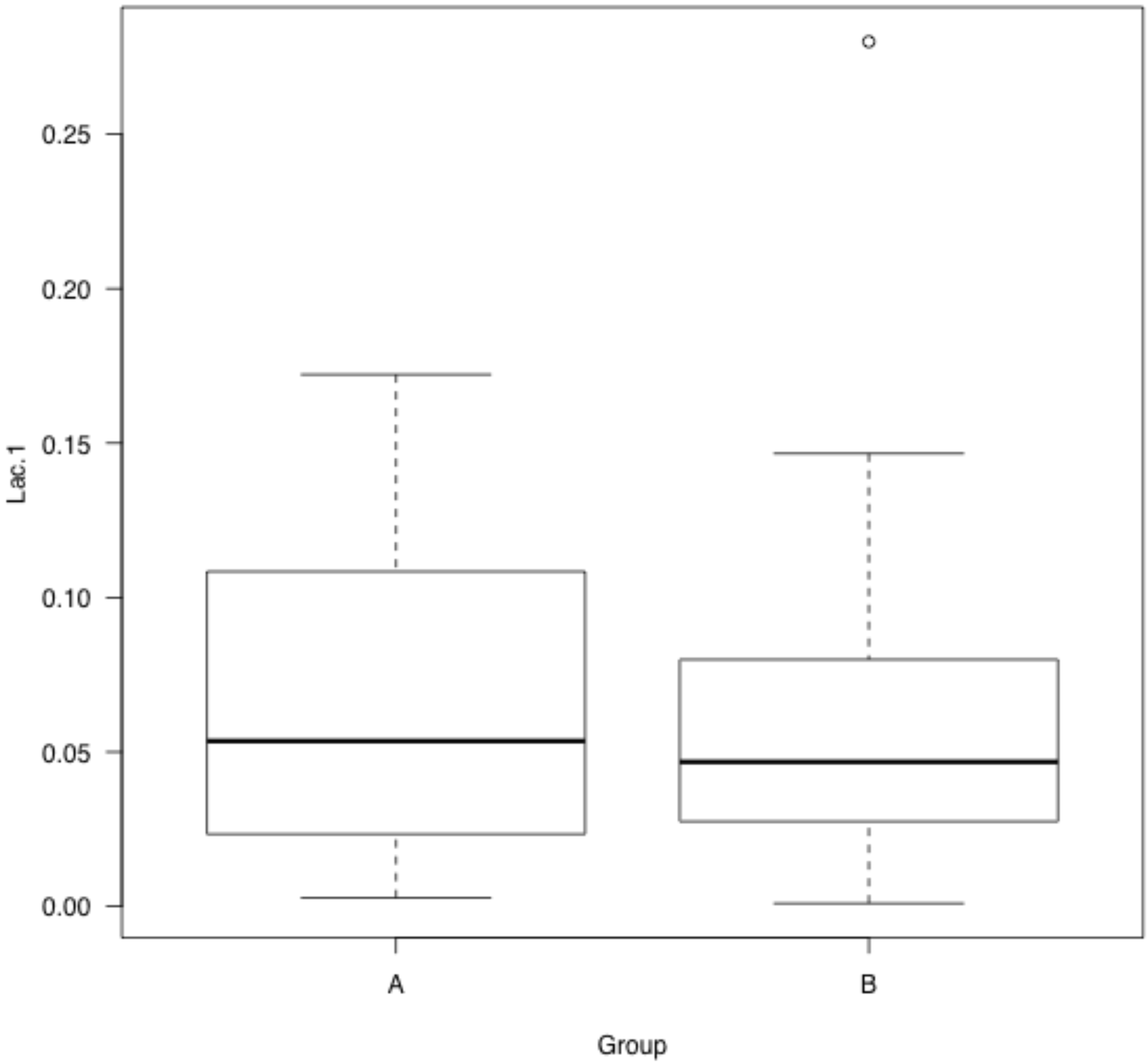
0.25
0.20
0.15
0.10
0.05
0.00
Lac.1
A
B
Group

```
> bar.means <- tapply(primary_data$Lac.1, factor(primary_data$Group), mea
> bar.sds <- tapply(primary_data$Lac.1, factor(primary_data$Group), sd, n
> group.names <- c(group.names, "Group=A")
> group.means <- c(group.means, bar.means[1])
> group.sds <- c(group.sds, bar.sds[1])
> group.p <- c(group.p, signif(res$p.value,digits=3))
> group.names <- c(group.names, "Group=B")
> group.means <- c(group.means, bar.means[2])
> group.sds <- c(group.sds, bar.sds[2])
> group.p <- c(group.p, "")
> summary.ttest <- NULL
> summary.ttest <- data.frame(mean=group.means, sd=group.sds, p.value=gro
> rownames(summary.ttest) <- group.names
> colnames(summary.ttest) <- gettext(domain="R-RcmdrPlugin.EZR",colnames(
> summary.ttest
```

```
              mean         sd p.value
Group=A 0.07040250 0.05539498   0.945
Group=B 0.06868545 0.07361109
```

```
> #####ANCOVA#####
+ TempDF <-  primary_data
+ quartz(width=7, height=7); par(lwd=1, las=1, family="sans", cex=1, mgp=
+ scatterplot(Lac.2 ~ Lac.1 | factor(Group), reg.line=lm, smooth=FALSE, s
+   by.groups=TRUE, data=TempDF)
+ cat(gettext(domain="R-RcmdrPlugin.EZR",
+   "P value for interaction between grouping variable and covariate is")
+ ")
+ ANCOVA not performed due to significant interaction between grouping va
```

```
Error: <text>:9:8: unexpected symbol
8: ")
9: ANCOVA not
          ^
```

```
> #####Two-sample t-test#####
> group.names <- NULL
> group.means <- NULL
> group.sds <- NULL
> group.p <- NULL
> res <- NULL
> (res <- t.test(Lac.2~factor(Group), alternative='two.sided', conf.level
+   var.equal=FALSE, data=primary_data))
```

```
	Welch Two Sample t-test

data:  Lac.2 by factor(Group)
t = -0.67776, df = 14.961, p-value = 0.5083
alternative hypothesis: true difference in means is not equal to 0
95 percent confidence interval:
 -0.6391164  0.3307795
sample estimates:
mean in group A mean in group B 
      0.1337660       0.2879345 
```

```
> quartz(width=7, height=7); par(lwd=1, las=1, family="sans", cex=1, mgp=
> boxplot(Lac.2~ factor(Group), ylab="Lac.2", xlab="Group", data=primary_
```

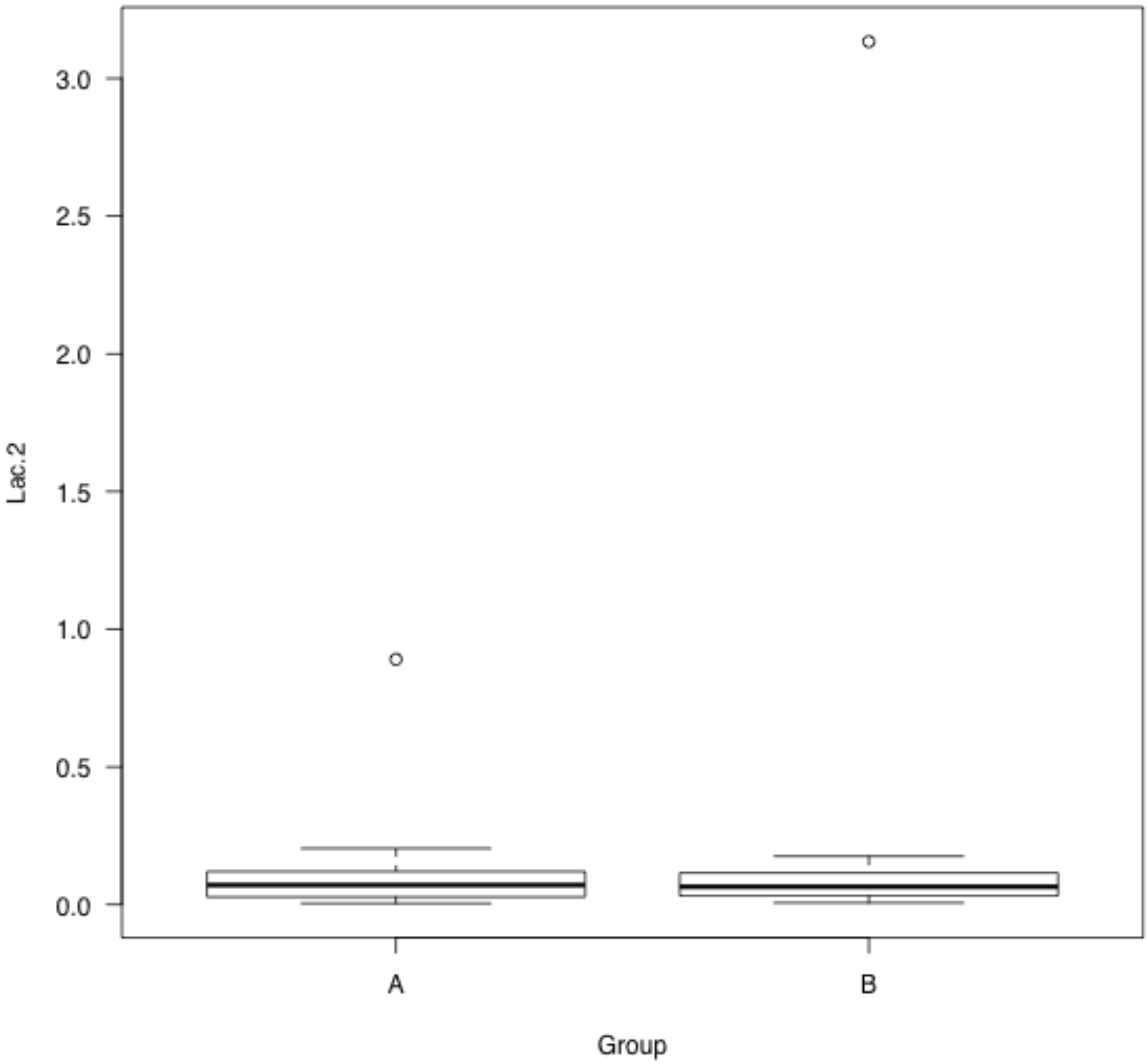
Lac.2
3.0
2.5
2.0
1.5
1.0
0.5
0.0
A
B
Group

```
> bar.means <- tapply(primary_data$Lac.2, factor(primary_data$Group), mea
> bar.sds <- tapply(primary_data$Lac.2, factor(primary_data$Group), sd, n
> group.names <- c(group.names, "Group=A")
> group.means <- c(group.means, bar.means[1])
> group.sds <- c(group.sds, bar.sds[1])
> group.p <- c(group.p, signif(res$p.value,digits=3))
> group.names <- c(group.names, "Group=B")
> group.means <- c(group.means, bar.means[2])
> group.sds <- c(group.sds, bar.sds[2])
> group.p <- c(group.p, "")
> summary.ttest <- NULL
> summary.ttest <- data.frame(mean=group.means, sd=group.sds, p.value=gro
> rownames(summary.ttest) <- group.names
> colnames(summary.ttest) <- gettext(domain="R-RcmdrPlugin.EZR",colnames(
> summary.ttest
```

```
             mean        sd p.value
Group=A 0.1337660 0.2259942   0.508
Group=B 0.2879345 0.8205568
```

```
> #####ANCOVA#####
+ TempDF <-  primary_data
+ quartz(width=7, height=7); par(lwd=1, las=1, family="sans", cex=1, mgp=
+ scatterplot(Lac.3 ~ Lac.1 | factor(Group), reg.line=lm, smooth=FALSE, s
+   by.groups=TRUE, data=TempDF)
+ cat(gettext(domain="R-RcmdrPlugin.EZR",
+   "P value for interaction between grouping variable and covariate is")
+ ")
+ ANCOVA not performed due to significant interaction between grouping va
```

```
Error: <text>:9:8: unexpected symbol
8: ")
9: ANCOVA not
          ^
```

```
> #####Two-sample t-test#####
> group.names <- NULL
> group.means <- NULL
> group.sds <- NULL
> group.p <- NULL
> res <- NULL
> (res <- t.test(Lac.3~factor(Group), alternative='two.sided', conf.level
+   var.equal=FALSE, data=primary_data))
```

```

	Welch Two Sample t-test

data:  Lac.3 by factor(Group)
t = -1.148, df = 17.098, p-value = 0.2668
alternative hypothesis: true difference in means is not equal to 0
95 percent confidence interval:
 -0.23124066  0.06822562
sample estimates:
mean in group A mean in group B 
     0.08202325      0.16353077 
```

```
> quartz(width=7, height=7); par(lwd=1, las=1, family="sans", cex=1, mgp=
> boxplot(Lac.3~ factor(Group), ylab="Lac.3", xlab="Group", data=primary_
```

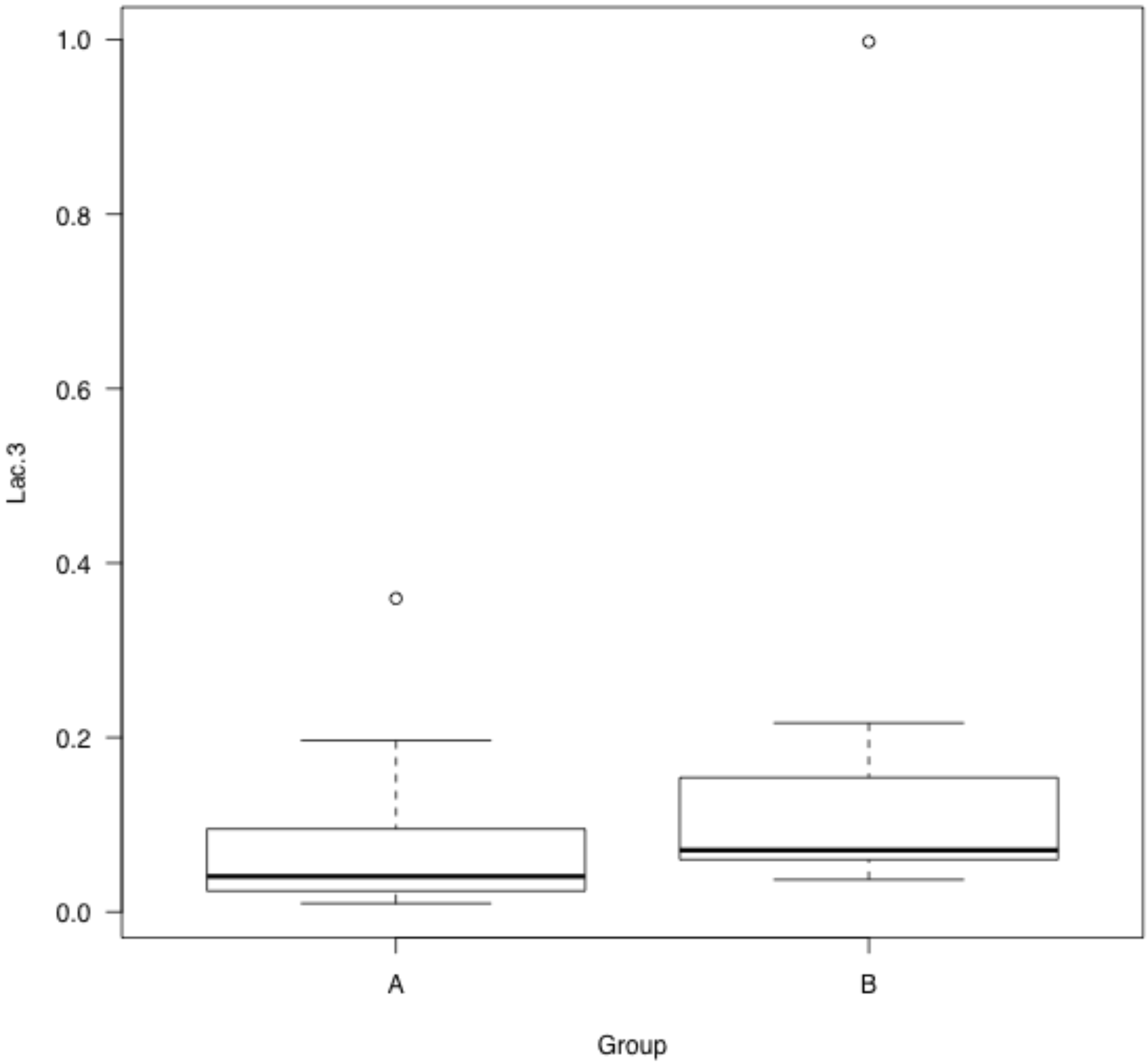

1.0
0.8
0.6
0.4
0.2
0.0
Lac.3
A
B
Group

```
> bar.means <- tapply(primary_data$Lac.3, factor(primary_data$Group), mea
> bar.sds <- tapply(primary_data$Lac.3, factor(primary_data$Group), sd, n
> group.names <- c(group.names, "Group=A")
> group.means <- c(group.means, bar.means[1])
> group.sds <- c(group.sds, bar.sds[1])
> group.p <- c(group.p, signif(res$p.value,digits=3))
> group.names <- c(group.names, "Group=B")
> group.means <- c(group.means, bar.means[2])
> group.sds <- c(group.sds, bar.sds[2])
> group.p <- c(group.p, "")
> summary.ttest <- NULL
> summary.ttest <- data.frame(mean=group.means, sd=group.sds, p.value=gro
> rownames(summary.ttest) <- group.names
> colnames(summary.ttest) <- gettext(domain="R-RcmdrPlugin.EZR",colnames(
> summary.ttest
```

```
              mean         sd p.value
Group=A 0.08202325 0.09912205   0.267
Group=B 0.16353077 0.24647560
```

```
> #####Two-sample t-test#####
> group.names <- NULL
> group.means <- NULL
> group.sds <- NULL
> group.p <- NULL
> res <- NULL
> (res <- t.test(Man.1~factor(Group), alternative='two.sided', conf.level
+   var.equal=FALSE, data=primary_data))
```

```


    Welch Two Sample t-test

data:  Man.1 by factor(Group)
t = -0.18537, df = 23.185, p-value = 0.8545
alternative hypothesis: true difference in means is not equal to 0
95 percent confidence interval:
 -6.417720  5.361695
sample estimates:
mean in group A mean in group B
       7.633565        8.161577
```

```
> quartz(width=7, height=7); par(lwd=1, las=1, family="sans", cex=1, mgp=
> boxplot(Man.1~ factor(Group), ylab="Man.1", xlab="Group", data=primary_
```

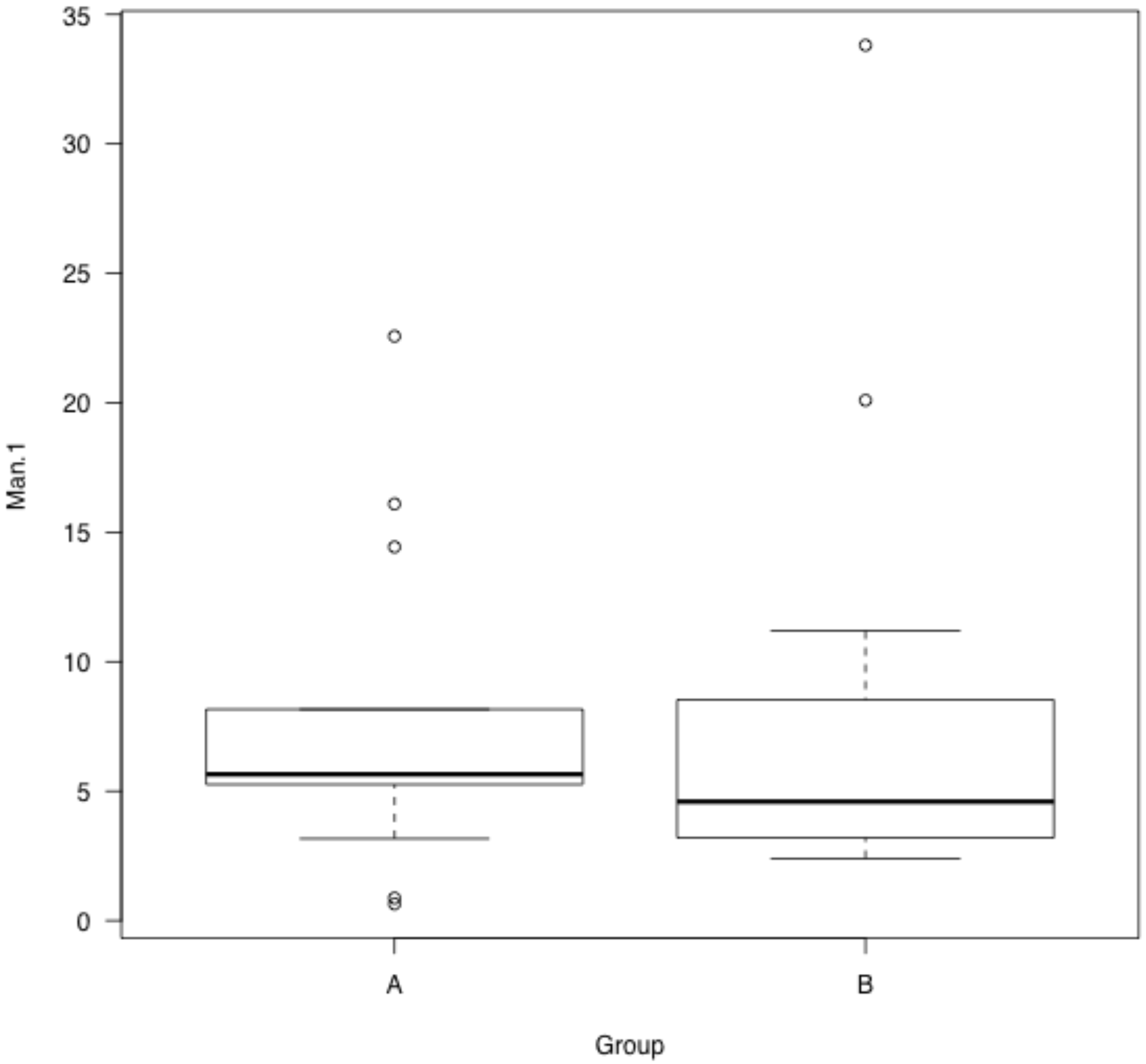

35
30
25
20
15
10
5
0
Man.1
A
B
Group

```
> bar.means <- tapply(primary_data$Man.1, factor(primary_data$Group), mea
> bar.sds <- tapply(primary_data$Man.1, factor(primary_data$Group), sd, n
> group.names <- c(group.names, "Group=A")
> group.means <- c(group.means, bar.means[1])
> group.sds <- c(group.sds, bar.sds[1])
> group.p <- c(group.p, signif(res$p.value,digits=3))
> group.names <- c(group.names, "Group=B")
> group.means <- c(group.means, bar.means[2])
> group.sds <- c(group.sds, bar.sds[2])
> group.p <- c(group.p, "")
> summary.ttest <- NULL
> summary.ttest <- data.frame(mean=group.means, sd=group.sds, p.value=gro
> rownames(summary.ttest) <- group.names
> colnames(summary.ttest) <- gettext(domain="R-RcmdrPlugin.EZR",colnames(
> summary.ttest
```

```
            mean       sd p.value
Group=A 7.633565 6.082981   0.855
Group=B 8.161577 8.751138
```

```
> #####ANCOVA#####
> TempDF <-  primary_data
> quartz(width=7, height=7); par(lwd=1, las=1, family="sans", cex=1, mgp=
> scatterplot(Man.2 ~ Man.1 | factor(Group), reg.line=lm, smooth=FALSE, s
+   by.groups=TRUE, data=TempDF)
```

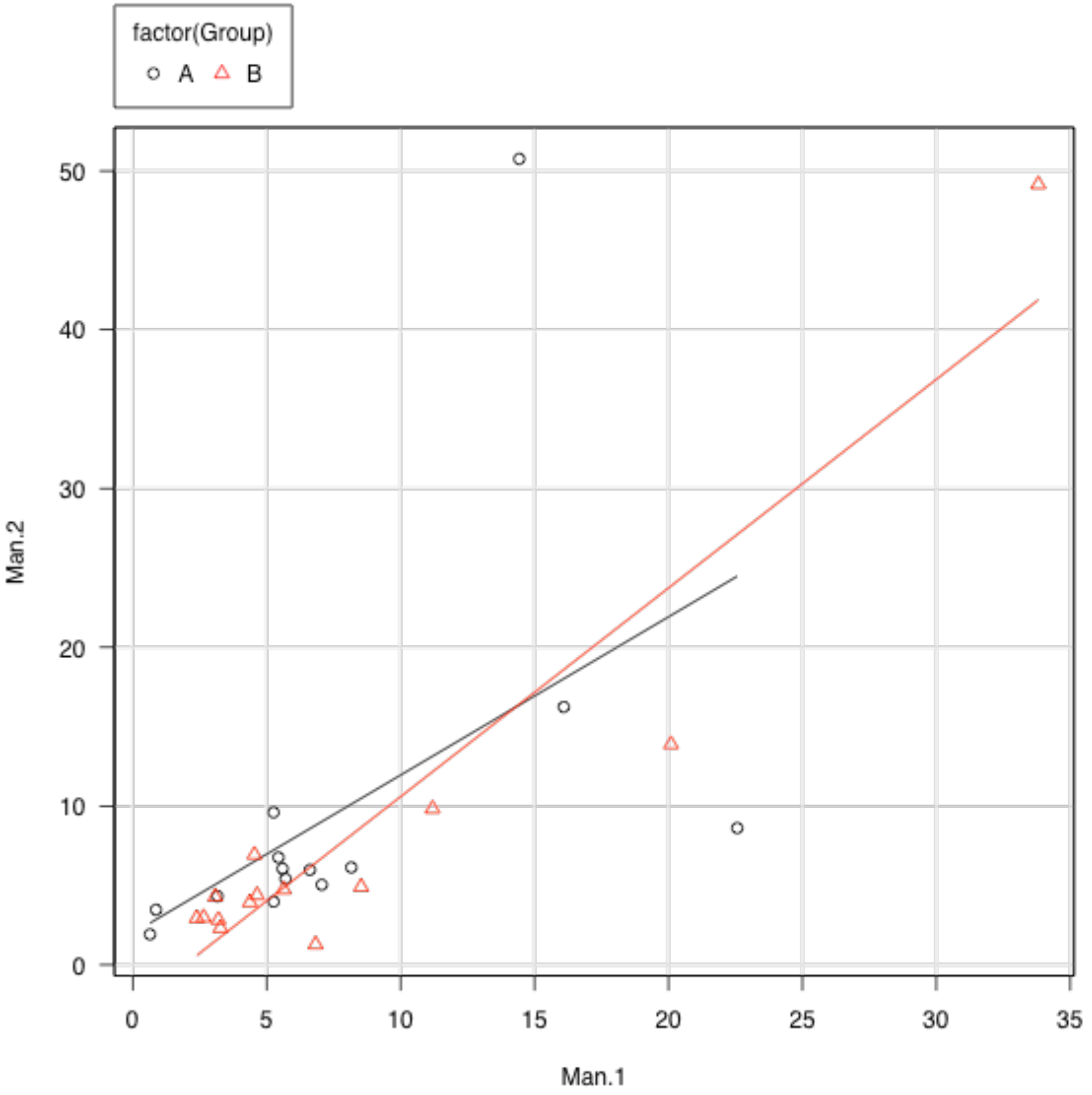


```
> cat(gettext(domain="R-RcmdrPlugin.EZR",
+   "P value for interaction between grouping variable and covariate is")
+ ")
```

```
P value for interaction between grouping variable and covariate is 0.507
```

```
> AnovaModel.5 <- AnovaModel.5 <- lm(Man.2 ~ 1 + factor(Group) + Man.1, d
+   na.action=na.omit)
> Anova(AnovaModel.5, type="III")
```

```
Anova Table (Type III tests)

Response: Man.2
               Sum Sq Df F value    Pr(>F)
(Intercept)      1.09  1  0.0155    0.9020
factor(Group)   29.62  1  0.4194    0.5231
Man.1         2164.82  1 30.6517 9.367e-06 ***
Residuals     1765.67 25
---
Signif. codes:  0 '***' 0.001 '**' 0.01 '*' 0.05 '.' 0.1 ' ' 1
```

```
> #####ANCOVA#####
+ TempDF <-  primary_data
+ quartz(width=7, height=7); par(lwd=1, las=1, family="sans", cex=1, mgp=
+ scatterplot(Man.3 ~ Man.1 | factor(Group), reg.line=lm, smooth=FALSE, s
+   by.groups=TRUE, data=TempDF)
+ cat(gettext(domain="R-RcmdrPlugin.EZR",
+   "P value for interaction between grouping variable and covariate is")
+ ")
+ ANCOVA not performed due to significant interaction between grouping va
```

```
Error: <text>:9:8: unexpected symbol
8: ")
9: ANCOVA not
          ^
```

```
> #####Two-sample t-test#####
> group.names <- NULL
> group.means <- NULL
> group.sds <- NULL
> group.p <- NULL
> res <- NULL
> (res <- t.test(Man.3~factor(Group), alternative='two.sided', conf.level
+   var.equal=FALSE, data=primary_data))
```

```
	Welch Two Sample t-test

data:  Man.3 by factor(Group)
t = 0.070068, df = 21.401, p-value = 0.9448
alternative hypothesis: true difference in means is not equal to 0
95 percent confidence interval:
 -5.558782  5.946879
sample estimates:
mean in group A mean in group B 
       8.725672        8.531623 
```

```
> quartz(width=7, height=7); par(lwd=1, las=1, family="sans", cex=1, mgp=
> boxplot(Man.3~ factor(Group), ylab="Man.3", xlab="Group", data=primary_
```

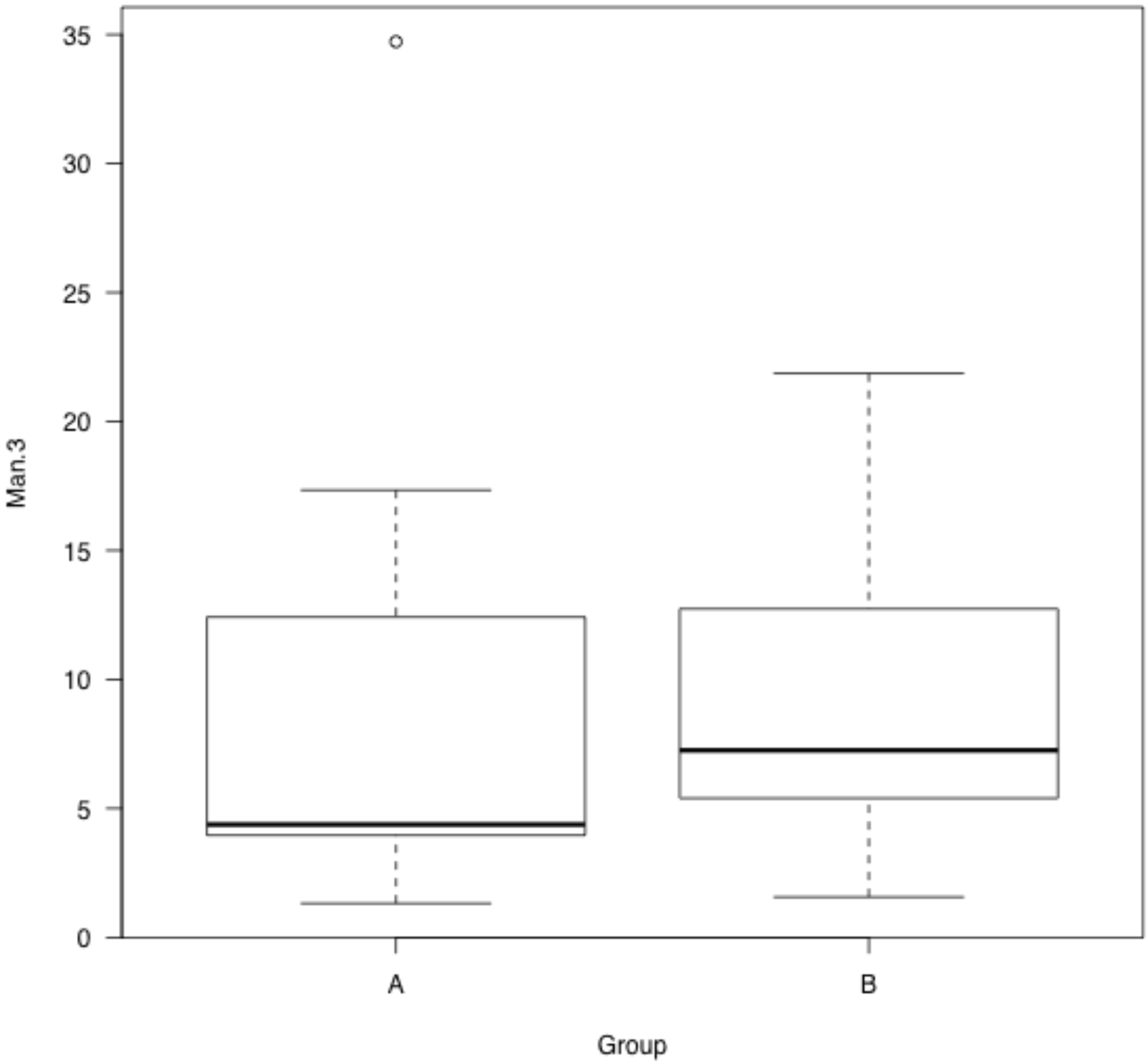
35
30
25
20
15
10
5
0
Man.3
A
B
Group

```
> bar.means <- tapply(primary_data$Man.3, factor(primary_data$Group), mea
> bar.sds <- tapply(primary_data$Man.3, factor(primary_data$Group), sd, n
> group.names <- c(group.names, "Group=A")
> group.means <- c(group.means, bar.means[1])
> group.sds <- c(group.sds, bar.sds[1])
> group.p <- c(group.p, signif(res$p.value,digits=3))
> group.names <- c(group.names, "Group=B")
> group.means <- c(group.means, bar.means[2])
> group.sds <- c(group.sds, bar.sds[2])
> group.p <- c(group.p, "")
> summary.ttest <- NULL
> summary.ttest <- data.frame(mean=group.means, sd=group.sds, p.value=gro
> rownames(summary.ttest) <- group.names
> colnames(summary.ttest) <- gettext(domain="R-RcmdrPlugin.EZR",colnames(
> summary.ttest
```

```
            mean       sd p.value
Group=A 8.725672 8.864379   0.945
Group=B 8.531623 5.366668
```

```
> #####Two-sample t-test#####
> group.names <- NULL
> group.means <- NULL
> group.sds <- NULL
> group.p <- NULL
> res <- NULL
> (res <- t.test(EAA1~factor(Group), alternative='two.sided', conf.level=
+    data=primary_data))
```

```


    Welch Two Sample t-test

data:  EAA1 by factor(Group)
t = -1.5893, df = 22.571, p-value = 0.1259
alternative hypothesis: true difference in means is not equal to 0
95 percent confidence interval:
 -0.14969488  0.01969488
sample estimates:
mean in group A mean in group B 
      0.1307143       0.1957143 
```

```
> quartz(width=7, height=7); par(lwd=1, las=1, family="sans", cex=1, mgp=
> boxplot(EAA1~ factor(Group), ylab="EAA1", xlab="Group", data=primary_da
```

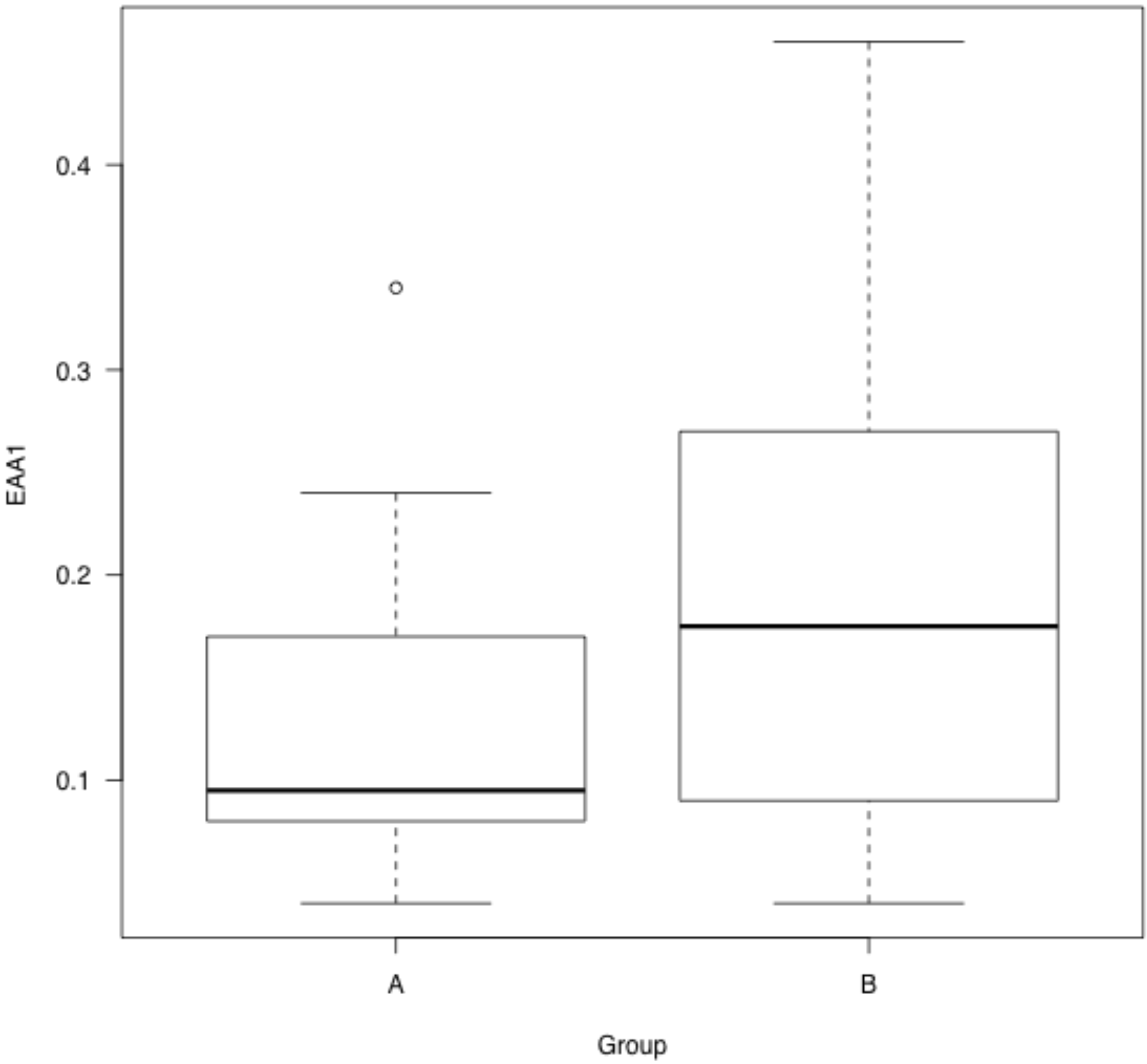
EAA1
0.1
0.2
0.3
0.4
A
B
Group

```
> bar.means <- tapply(primary_data$EAA1, factor(primary_data$Group), mean
> bar.sds <- tapply(primary_data$EAA1, factor(primary_data$Group), sd, na
> group.names <- c(group.names, "Group=A")
> group.means <- c(group.means, bar.means[1])
> group.sds <- c(group.sds, bar.sds[1])
> group.p <- c(group.p, signif(res$p.value,digits=3))
> group.names <- c(group.names, "Group=B")
> group.means <- c(group.means, bar.means[2])
> group.sds <- c(group.sds, bar.sds[2])
> group.p <- c(group.p, "")
> summary.ttest <- NULL
> summary.ttest <- data.frame(mean=group.means, sd=group.sds, p.value=gro
> rownames(summary.ttest) <- group.names
> colnames(summary.ttest) <- gettext(domain="R-RcmdrPlugin.EZR",colnames(
> summary.ttest
```

```
             mean         sd p.value
Group=A 0.1307143 0.08453168   0.126
Group=B 0.1957143 0.12756382
```

```
> #####ANCOVA#####
> TempDF <-  primary_data
> quartz(width=7, height=7); par(lwd=1, las=1, family="sans", cex=1, mgp=
> scatterplot(EAA2 ~ EAA1 | factor(Group), reg.line=lm, smooth=FALSE, spr
+   by.groups=TRUE, data=TempDF)
```

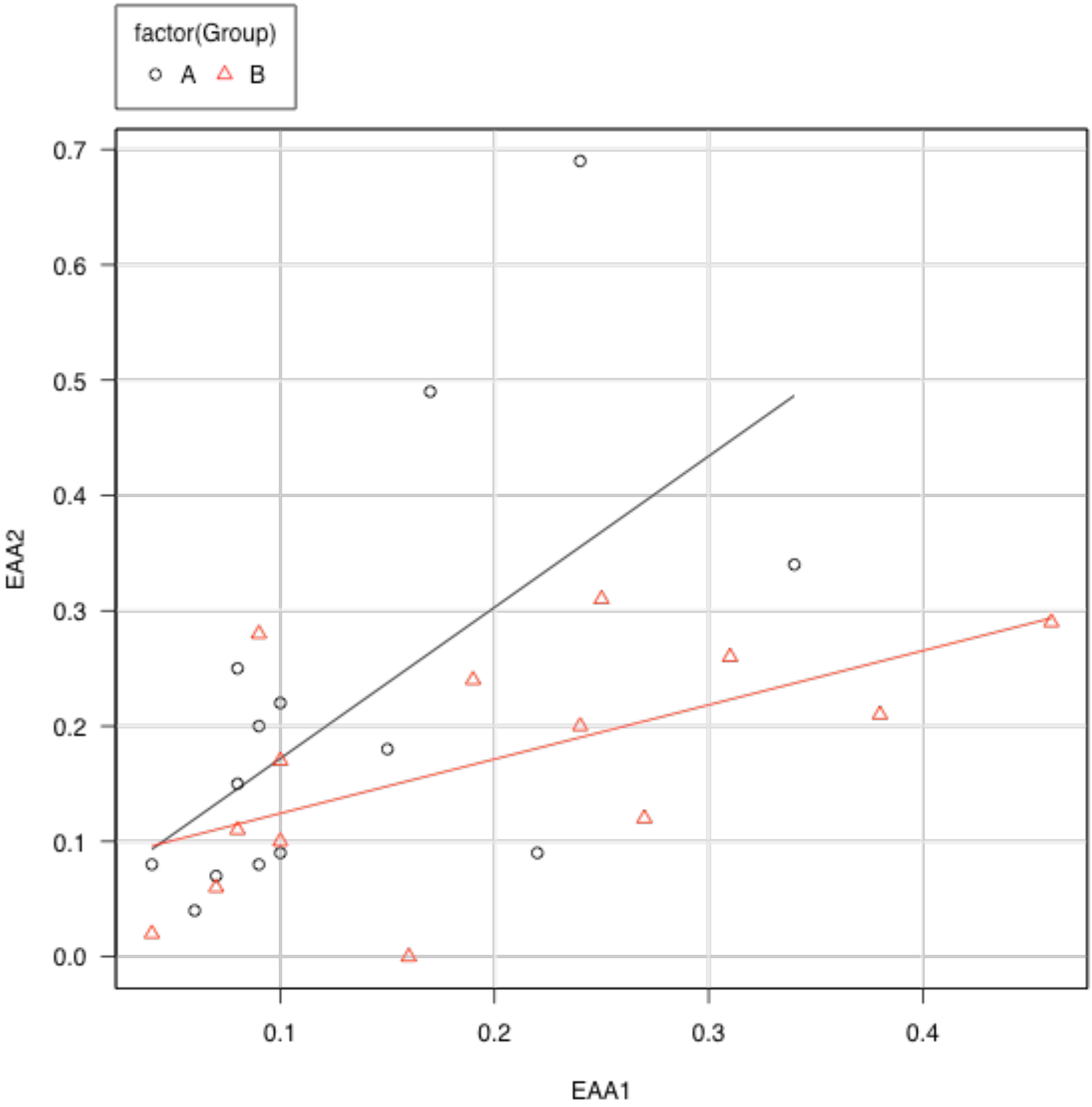


```
> cat(gettext(domain="R-RcmdrPlugin.EZR",
+   "P value for interaction between grouping variable and covariate is")
+ ")
```

```
P value for interaction between grouping variable and covariate is 0.0994
```

```
> AnovaModel.7 <- AnovaModel.7 <- lm(EAA2 ~ 1 + factor(Group) + EAA1, dat
+   na.action=na.omit)
> Anova(AnovaModel.7, type="III")
```

```
Anova Table (Type III tests)

Response: EAA2
               Sum Sq Df F value   Pr(>F)
(Intercept)   0.10760  1  6.4299 0.017848 *
factor(Group) 0.05179  1  3.0947 0.090785 .
EAA1          0.16077  1  9.6075 0.004747 **
Residuals     0.41835 25
---
Signif. codes:  0 '***' 0.001 '**' 0.01 '*' 0.05 '.' 0.1 ' ' 1
```

```
> #####ANCOVA#####
> TempDF <-  primary_data
> quartz(width=7, height=7); par(lwd=1, las=1, family="sans", cex=1, mgp=
> scatterplot(EAA3 ~ EAA1 | factor(Group), reg.line=lm, smooth=FALSE, spr
+   by.groups=TRUE, data=TempDF)
```

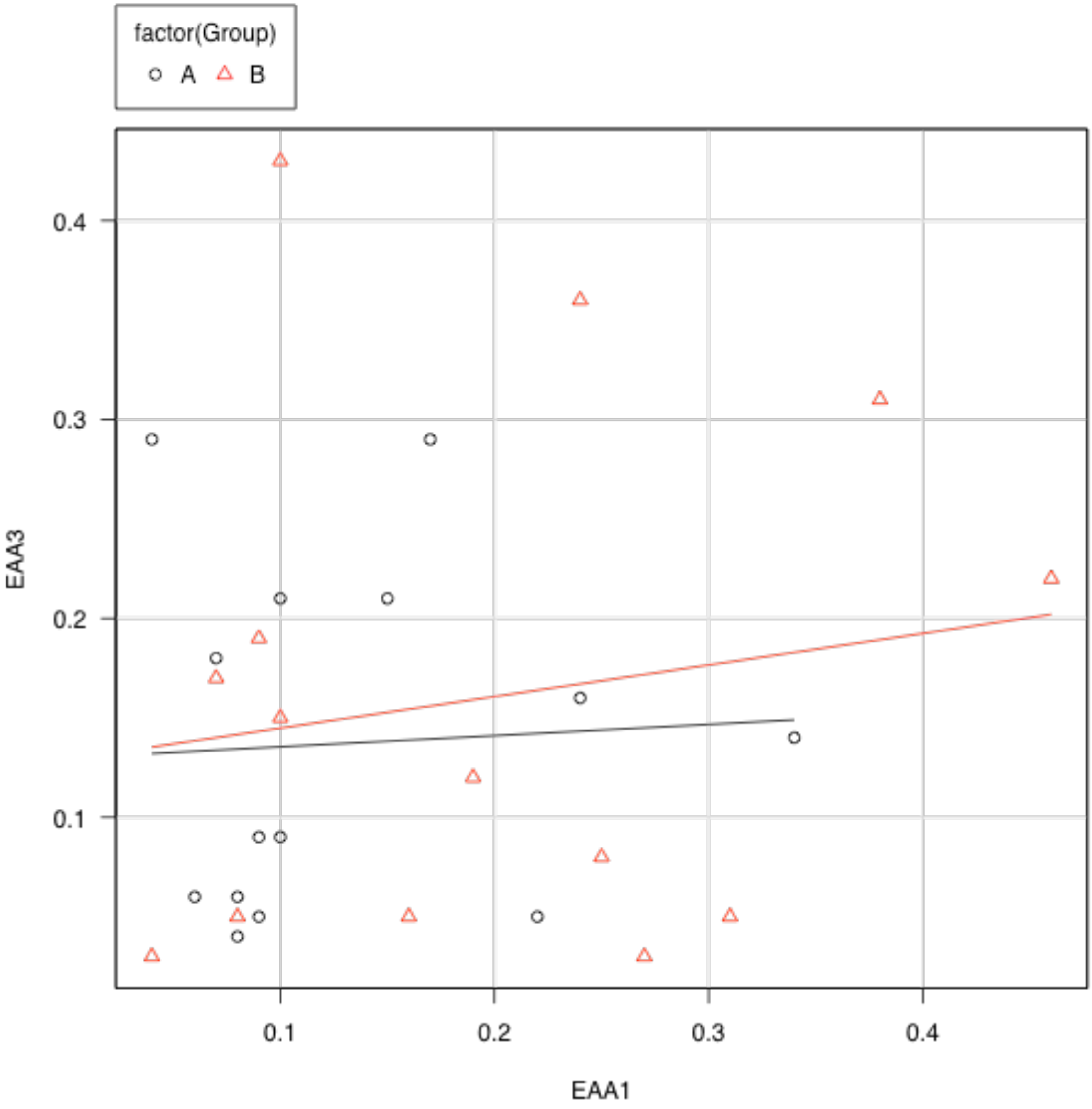


```
> cat(gettext(domain="R-RcmdrPlugin.EZR",
+   "P value for interaction between grouping variable and covariate is")
+ ")
```

```
P value for interaction between grouping variable and covariate is 0.822
```

```
> AnovaModel.8 <- AnovaModel.8 <- lm(EAA3 ~ 1 + factor(Group) + EAA1, dat
+   na.action=na.omit)
> Anova(AnovaModel.8, type="III")
```

```
Anova Table (Type III tests)

Response: EAA3
                Sum Sq Df F value   Pr(>F)
(Intercept)   0.113783  1  9.0611 0.005893 **
factor(Group) 0.001354  1  0.1078 0.745383
EAA1          0.004952  1  0.3944 0.535704
Residuals     0.313933 25
---
Signif. codes:  0 '***' 0.001 '**' 0.01 '*' 0.05 '.' 0.1 ' ' 1
```

```
> #####Read Data From Text File#####
> AE <- read.table("/Users/takakato222/Desktop/lubiprostone adverse event
+   sep=",", na.strings=c("", "NA"), dec=".", fill=TRUE, quote="\"", stri
> AE <- read.table("/Users/takakato222/Desktop/lubiprostone adverse event
+   sep=",", na.strings=c("", "NA"), dec=".", fill=TRUE, quote="\"", stri
```

```
> library(abind, pos=16)
```

```
> #####Create two-way table and compare two proportions (Fisher's exact t
> Fisher.summary.table <- NULL
> .Table <- NULL
> .Table <- xtabs(~AE+Group, data=AE)
> .Table
```

```
   Group
AE   A  B
  0 11 15
  1  4  0
```

```
> rowPercents(.Table) # Row Percentages
```

```
   Group
AE      A    B Total Count
  0  42.3 57.7   100    26
  1 100.0  0.0   100     4
```

```
> fisher.test(.Table)
```

```
	Fisher's Exact Test for Count Data

data:  .Table
p-value = 0.09962
alternative hypothesis: true odds ratio is not equal to 1
95 percent confidence interval:
 0.000000 1.379265
sample estimates:
odds ratio
         0
```

```
> res <- NULL
> res <- fisher.test(.Table)
> Fisher.summary.table <- rbind(Fisher.summary.table,
+   summary.table.twoway(table=.Table, res=res))
> colnames(Fisher.summary.table)[length(Fisher.summary.table)] <-
+   gettext(domain="R-RcmdrPlugin.EZR",
+   colnames(Fisher.summary.table)[length(Fisher.summary.table)])
> Fisher.summary.table
```

```
     Group=A Group=B Fisher.p.value
AE=0      11      15         0.0996
AE=1       4       0
```
