# Supplementary material for: Lubiprostone improves intestinal permeability in humans, a novel therapy for the leaky gut: A prospective randomized pilot study in healthy volunteers
Source: PLoS One. 2017 Apr 14;12(4):e0175626. doi: 10.1371/journal.pone.0175626 (PMC5391961; doi:10.1371/journal.pone.0175626)
Supplement: S3 File — (PDF) [file pone.0175626.s005.pdf]

Replace with Main Title

Your Name

2017-02-23

```
> primary_data <-
+   readXL("C:/Users/Takayuki Kato/iCloudDrive/アボット研究/PLOS ONE 投
+    rownames=FALSE, header=TRUE, na="", sheet="primary data.csv",
+   stringsAsFactors=TRUE)
```

```
DEFINEDNAME: 21 00 00 01 0b 00 00 00 01 00 00 00 00 00 00 0d 3b 00 00
DEFINEDNAME: 21 00 00 01 0b 00 00 00 01 00 00 00 00 00 00 0d 3b 00 00
DEFINEDNAME: 21 00 00 01 0b 00 00 00 01 00 00 00 00 00 00 0d 3b 00 00
DEFINEDNAME: 21 00 00 01 0b 00 00 00 01 00 00 00 00 00 00 0d 3b 00 00
DEFINEDNAME: 21 00 00 01 0b 00 00 00 01 00 00 00 00 00 00 0d 3b 00 00
```

```
> #####Two-sample t-test#####
> group.names <- NULL
> group.means <- NULL
> group.sds <- NULL
> group.p <- NULL
> res <- NULL
> (res <- t.test(LMR2~factor(Group), alternative='two.sided', conf.le
+   var.equal=FALSE, data=primary_data))
```

```


    Welch Two Sample t-test

data:  LMR2 by factor(Group)
t = -0.86721, df = 17.324, p-value = 0.3977
alternative hypothesis: true difference in means is not equal to 0
95 percent confidence interval:
 -0.03800393  0.01584036
sample estimates:
mean in group A mean in group B
     0.02400967      0.03509145
```

```
> windows(width=7, height=7); par(lwd=1, las=1, family="sans", cex=1,
+   0))
> boxplot(LMR2~ factor(Group), ylab="LMR2", xlab="Group", data=primar
```

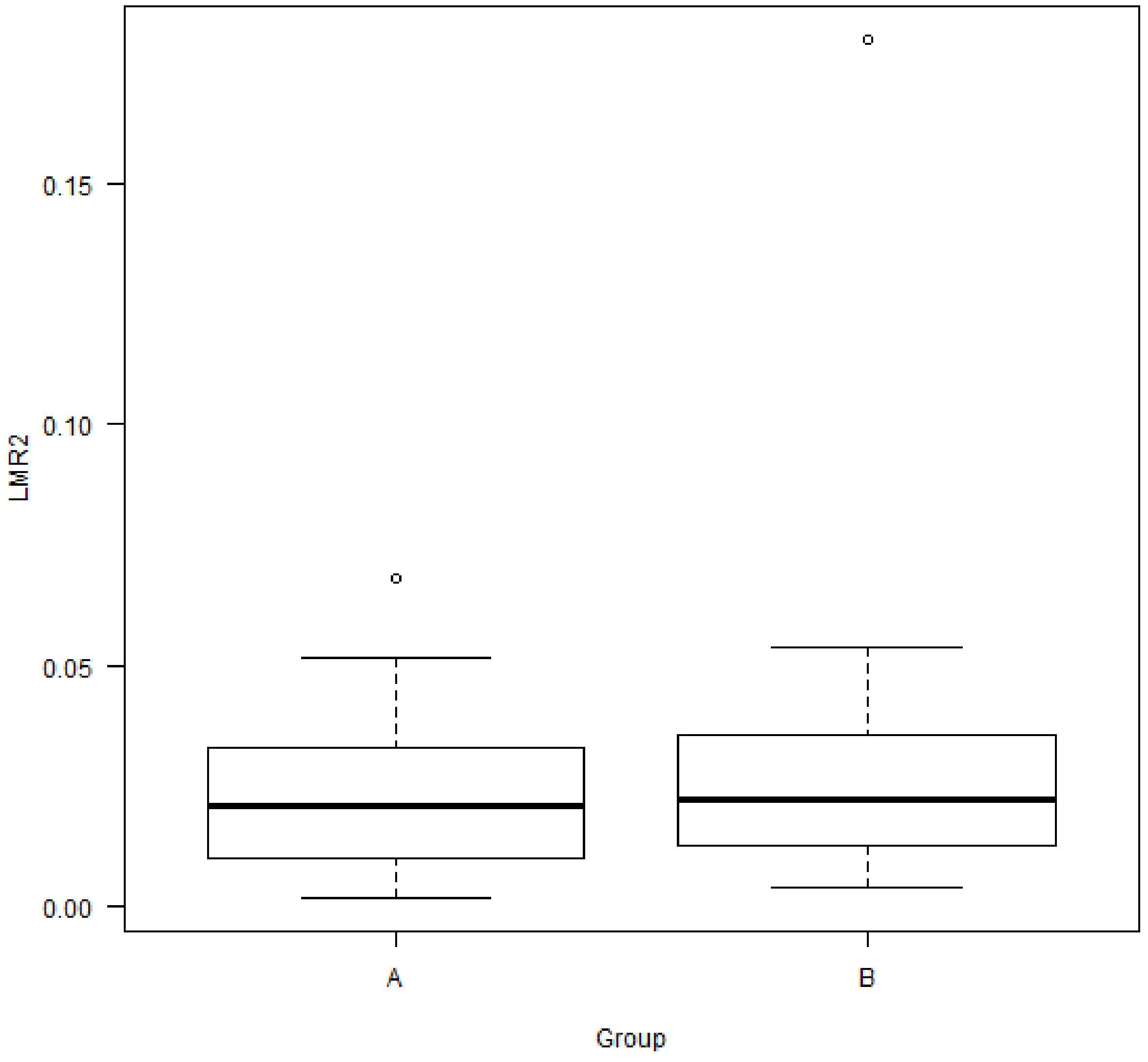

```
> bar.means <- tapply(primary_data$LMR2, factor(primary_data$Group),
+   na.rm=TRUE)
> bar.sds <- tapply(primary_data$LMR2, factor(primary_data$Group), sd
> group.names <- c(group.names, "Group=A")
> group.means <- c(group.means, bar.means[1])
> group.sds <- c(group.sds, bar.sds[1])
> group.p <- c(group.p, signif(res$p.value,digits=3))
> group.names <- c(group.names, "Group=B")
> group.means <- c(group.means, bar.means[2])
> group.sds <- c(group.sds, bar.sds[2])
> group.p <- c(group.p, "")
> summary.ttest <- NULL
> summary.ttest <- data.frame(mean=group.means, sd=group.sds, p.value
> rownames(summary.ttest) <- group.names
> colnames(summary.ttest) <- gettextRcmdr(colnames(summary.ttest))
> summary.ttest
```

```
              mean         sd p.value
Group=A 0.02400967 0.01827999   0.398
Group=B 0.03509145 0.04418084
```

```
> #####Two-sample t-test#####
> group.names <- NULL
> group.means <- NULL
> group.sds <- NULL
> group.p <- NULL
> res <- NULL
> (res <- t.test(LMR3~factor(Group), alternative='two.sided', conf.le
+   var.equal=FALSE, data=primary_data))
```

```


    Welch Two Sample t-test

data:  LMR3 by factor(Group)
t = -2.1496, df = 15.686, p-value = 0.04755
alternative hypothesis: true difference in means is not equal to 0
95 percent confidence interval:
 -0.0220759132 -0.0001358381
sample estimates:
mean in group A mean in group B
     0.01715622      0.02826209
```

```
> windows(width=7, height=7); par(lwd=1, las=1, family="sans", cex=1,
+   0))
> boxplot(LMR3~ factor(Group), ylab="LMR3", xlab="Group", data=primar
```

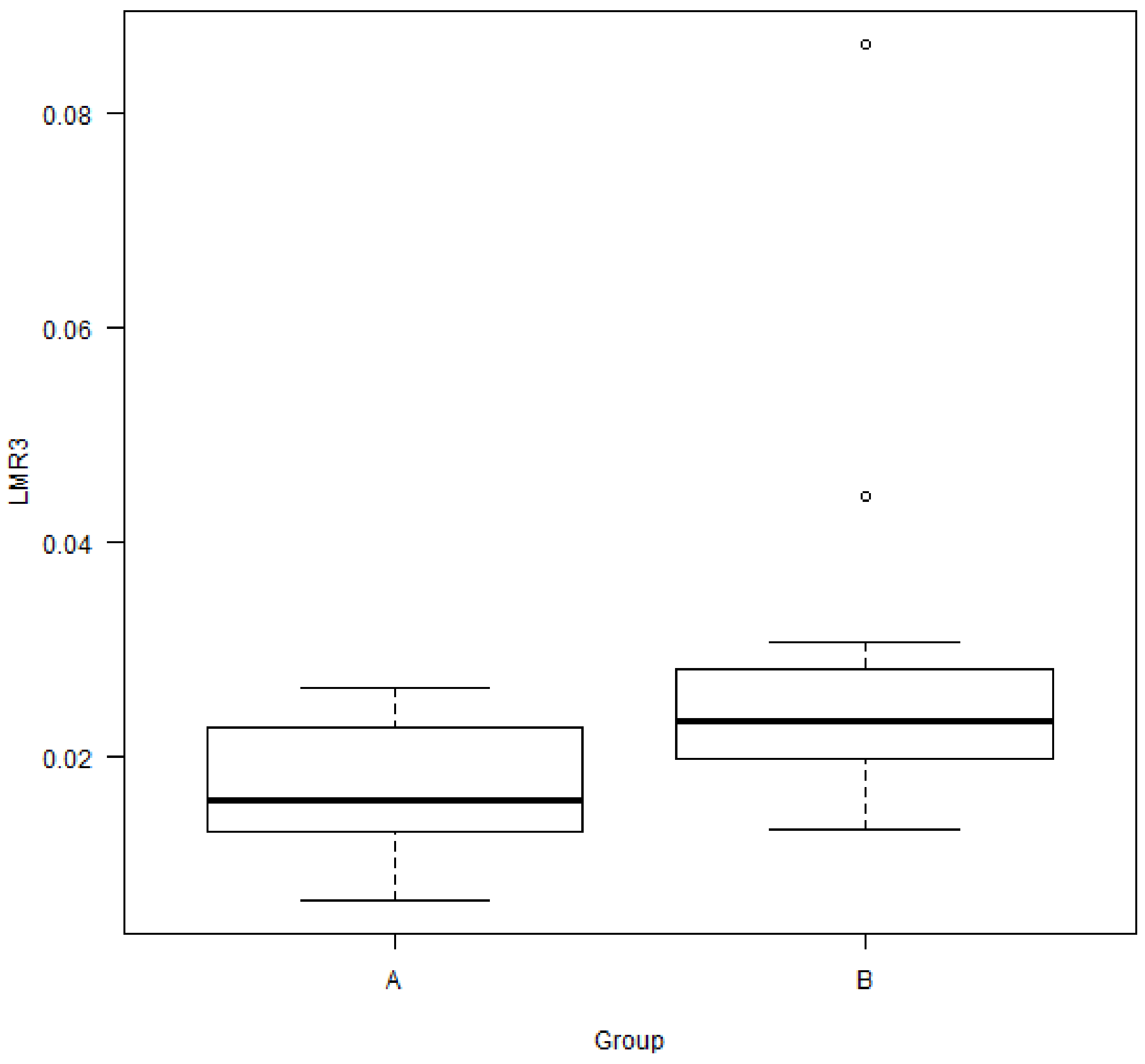

```
> bar.means <- tapply(primary_data$LMR3, factor(primary_data$Group),
+   na.rm=TRUE)
> bar.sds <- tapply(primary_data$LMR3, factor(primary_data$Group), sc
> group.names <- c(group.names, "Group=A")
> group.means <- c(group.means, bar.means[1])
> group.sds <- c(group.sds, bar.sds[1])
> group.p <- c(group.p, signif(res$p.value,digits=3))
> group.names <- c(group.names, "Group=B")
> group.means <- c(group.means, bar.means[2])
> group.sds <- c(group.sds, bar.sds[2])
> group.p <- c(group.p, "")
> summary.ttest <- NULL
> summary.ttest <- data.frame(mean=group.means, sd=group.sds, p.value
> rownames(summary.ttest) <- group.names
> colnames(summary.ttest) <- gettextRcmdr(colnames(summary.ttest))
> summary.ttest
```

```
              mean          sd p.value
Group=A 0.01715622 0.005944537  0.0475
Group=B 0.02826209 0.018394052
```

```
> #####Two-sample t-test#####
> group.names <- NULL
> group.means <- NULL
> group.sds <- NULL
> group.p <- NULL
> res <- NULL
> (res <- t.test(Man.2~factor(Group), alternative='two.sided', conf.l
+   var.equal=FALSE, data=primary_data))
```

```

    Welch Two Sample t-test

data:  Man.2 by factor(Group)
t = 0.30538, df = 25.999, p-value = 0.7625
alternative hypothesis: true difference in means is not equal to 0
95 percent confidence interval:
 -8.133243 10.971555
sample estimates:
mean in group A mean in group B
       9.591020        8.171863
```

```
> windows(width=7, height=7); par(lwd=1, las=1, family="sans", cex=1,
+   0))
> boxplot(Man.2~ factor(Group), ylab="Man.2", xlab="Group", data=prim
```

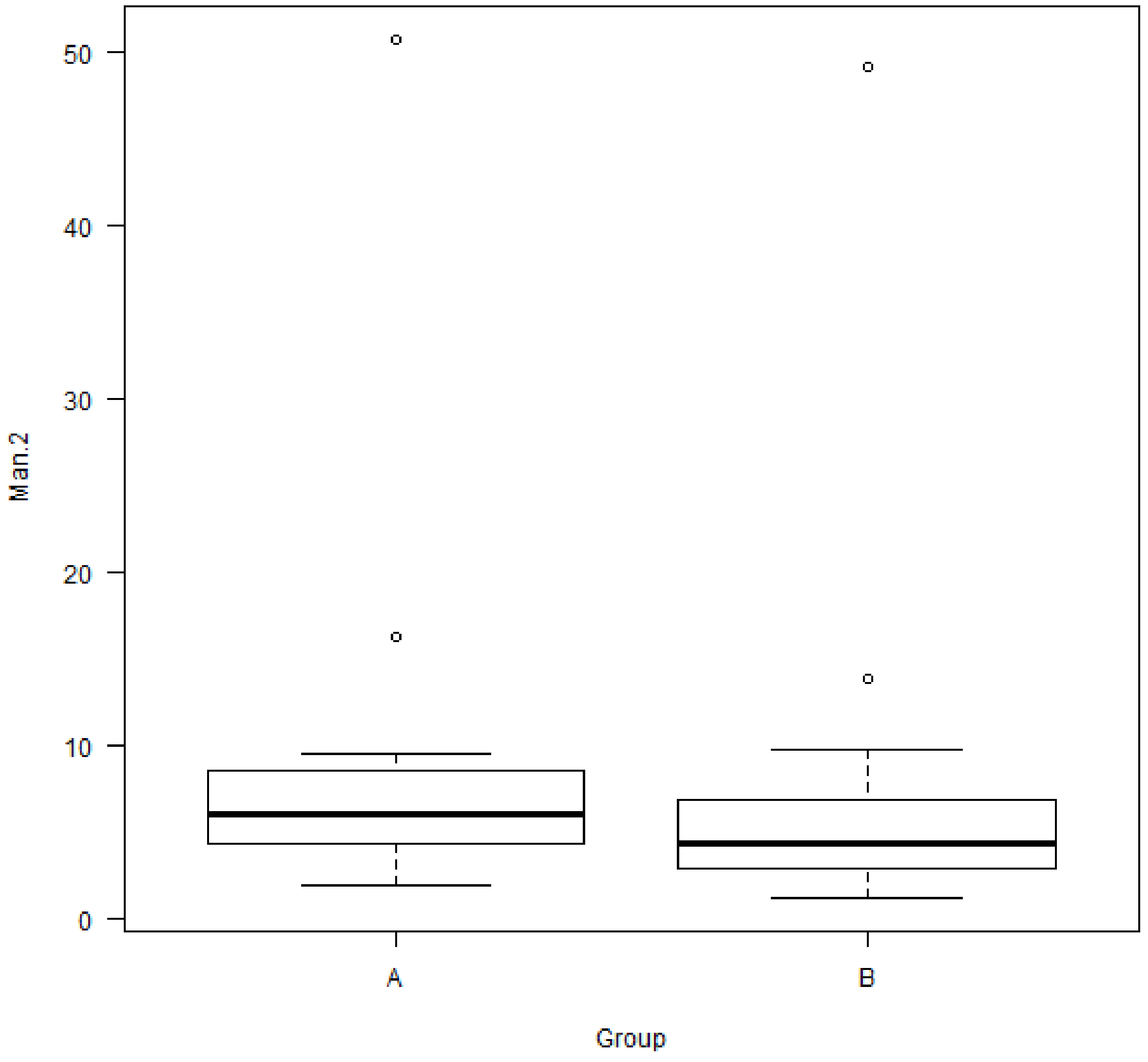

```
> bar.means <- tapply(primary_data$Man.2, factor(primary_data$Group),
+   na.rm=TRUE)
> bar.sds <- tapply(primary_data$Man.2, factor(primary_data$Group), s
> group.names <- c(group.names, "Group=A")
> group.means <- c(group.means, bar.means[1])
> group.sds <- c(group.sds, bar.sds[1])
> group.p <- c(group.p, signif(res$p.value,digits=3))
> group.names <- c(group.names, "Group=B")
> group.means <- c(group.means, bar.means[2])
> group.sds <- c(group.sds, bar.sds[2])
> group.p <- c(group.p, "")
> summary.ttest <- NULL
> summary.ttest <- data.frame(mean=group.means, sd=group.sds, p.value
> rownames(summary.ttest) <- group.names
> colnames(summary.ttest) <- gettextRcmdr(colnames(summary.ttest))
> summary.ttest
```

```
            mean       sd p.value
Group=A 9.591020 12.33969   0.763
Group=B 8.171863 12.25061
```

```
> #####Two-sample t-test#####
> group.names <- NULL
> group.means <- NULL
> group.sds <- NULL
> group.p <- NULL
> res <- NULL
> (res <- t.test(EAA2~factor(Group), alternative='two.sided', conf.le
+   var.equal=FALSE, data=primary_data))
```

```

    Welch Two Sample t-test

data:  EAA2 by factor(Group)
t = 0.75975, df = 20.298, p-value = 0.4561
alternative hypothesis: true difference in means is not equal to 0
95 percent confidence interval:
 -0.07470021  0.16041450
sample estimates:
mean in group A mean in group B 
      0.2121429       0.1692857 
```

```
> windows(width=7, height=7); par(lwd=1, las=1, family="sans", cex=1,
+   0))
> boxplot(EAA2~ factor(Group), ylab="EAA2", xlab="Group", data=primar
```

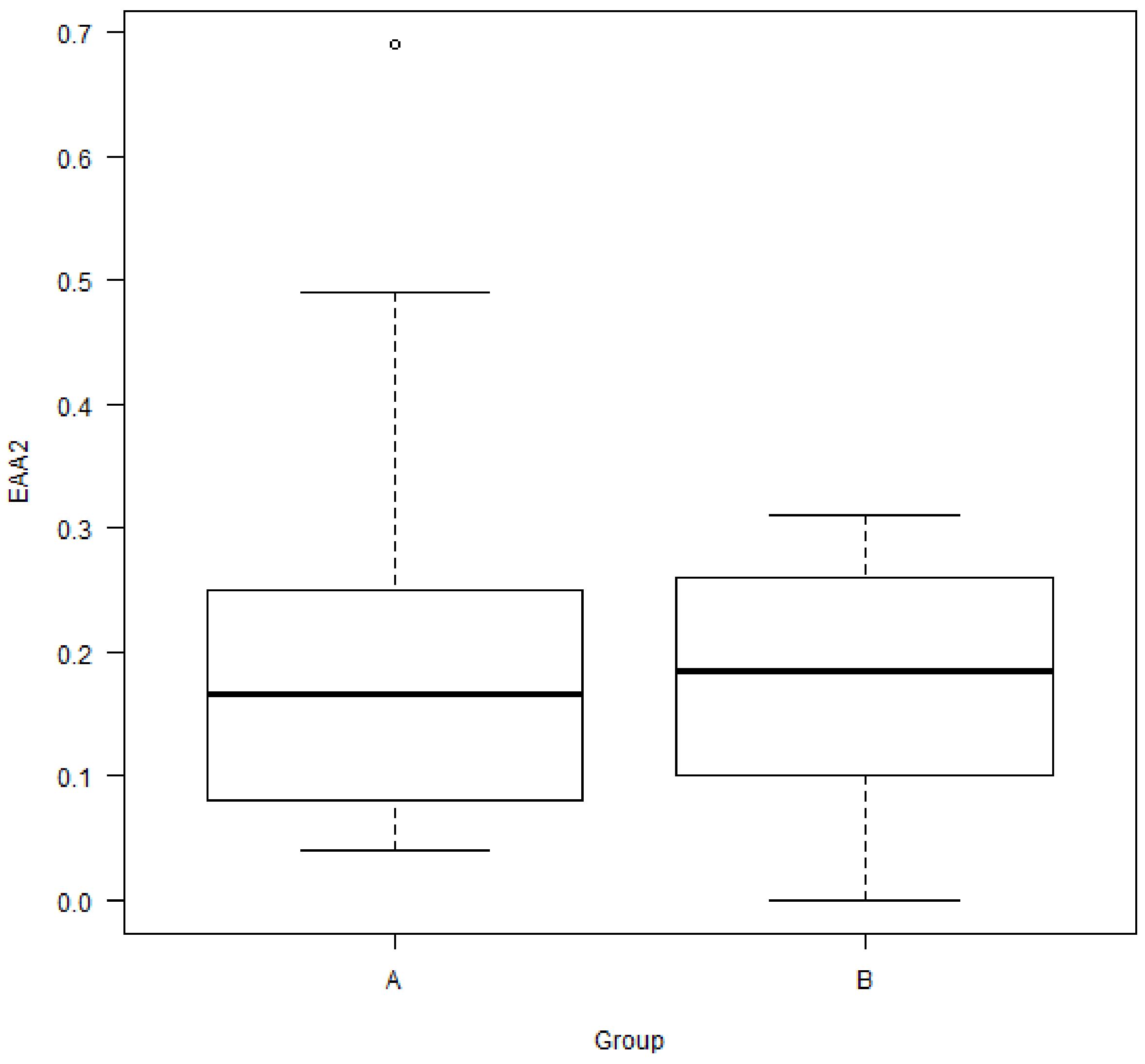

```
> bar.means <- tapply(primary_data$EAA2, factor(primary_data$Group),
+   na.rm=TRUE)
> bar.sds <- tapply(primary_data$EAA2, factor(primary_data$Group), sc
> group.names <- c(group.names, "Group=A")
> group.means <- c(group.means, bar.means[1])
> group.sds <- c(group.sds, bar.sds[1])
> group.p <- c(group.p, signif(res$p.value,digits=3))
> group.names <- c(group.names, "Group=B")
> group.means <- c(group.means, bar.means[2])
> group.sds <- c(group.sds, bar.sds[2])
> group.p <- c(group.p, "")
> summary.ttest <- NULL
> summary.ttest <- data.frame(mean=group.means, sd=group.sds, p.value
> rownames(summary.ttest) <- group.names
> colnames(summary.ttest) <- gettextRcmdr(colnames(summary.ttest))
> summary.ttest
```

```
             mean        sd p.value
Group=A 0.2121429 0.1846068   0.456
Group=B 0.1692857 0.1023166
```

```
> #####Two-sample t-test#####
> group.names <- NULL
> group.means <- NULL
> group.sds <- NULL
> group.p <- NULL
> res <- NULL
> (res <- t.test(EAA3~factor(Group), alternative='two.sided', conf.le
+   var.equal=FALSE, data=primary_data))
```

```

    Welch Two Sample t-test

data:  EAA3 by factor(Group)
t = -0.54606, df = 22.891, p-value = 0.5903
alternative hypothesis: true difference in means is not equal to 0
95 percent confidence interval:
 -0.10947045  0.06375616
sample estimates:
mean in group A mean in group B
      0.1371429       0.1600000
```

```
> windows(width=7, height=7); par(lwd=1, las=1, family="sans", cex=1,
+   0))
> boxplot(EAA3~ factor(Group), ylab="EAA3", xlab="Group", data=primar
```

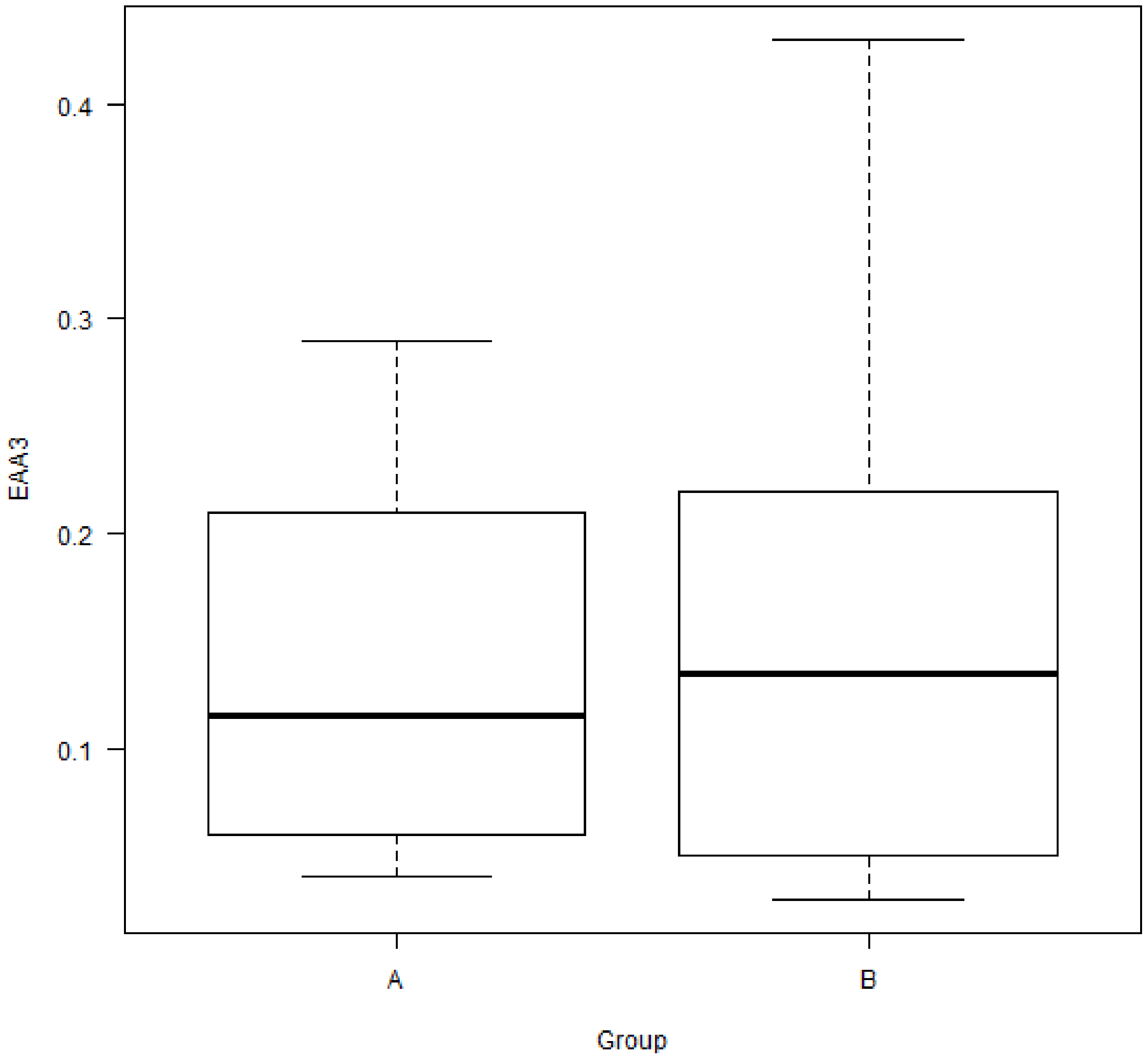

```
> bar.means <- tapply(primary_data$EAA3, factor(primary_data$Group),
+   na.rm=TRUE)
> bar.sds <- tapply(primary_data$EAA3, factor(primary_data$Group), sc
> group.names <- c(group.names, "Group=A")
> group.means <- c(group.means, bar.means[1])
> group.sds <- c(group.sds, bar.sds[1])
> group.p <- c(group.p, signif(res$p.value,digits=3))
> group.names <- c(group.names, "Group=B")
> group.means <- c(group.means, bar.means[2])
> group.sds <- c(group.sds, bar.sds[2])
> group.p <- c(group.p, "")
> summary.ttest <- NULL
> summary.ttest <- data.frame(mean=group.means, sd=group.sds, p.value
> rownames(summary.ttest) <- group.names
> colnames(summary.ttest) <- gettextRcmdr(colnames(summary.ttest))
> summary.ttest
```

```
             mean         sd p.value
Group=A 0.1371429 0.08800599    0.59
Group=B 0.1600000 0.12955545
```
